# On-site airborne pathogen detection for infection risk mitigation†

**DOI:** 10.1039/d3cs00417a

**Published:** 2023-10-26

**Authors:** Guangyu Qiu, Xiaole Zhang, Andrew J. deMello, Maosheng Yao, Junji Cao, Jing Wang

**Affiliations:** a Institute of Medical Robotics, School of Biomedical Engineering, Shanghai Jiao Tong University Shanghai 200240 China guangyuqiu@sjtu.edu.cn; b Institute of Environmental Engineering, ETH Zürich Zürich 8093 Switzerland; c Laboratory for Advanced Analytical Technologies, Empa, Swiss Federal Laboratories for Materials Science and Technology Dübendorf 8600 Switzerland; d Institute for Chemical and Bioengineering, Department of Chemistry and Applied Biosciences, ETH Zürich Vladimir-Prelog-Weg1 Zürich Switzerland; e State Key Joint Laboratory of Environmental Simulation and Pollution Control, College of Environmental Sciences and Engineering, Peking University China; f Institute of Atmospheric Physics, Chinese Academy of Science China

## Abstract

Human-infecting pathogens that transmit through the air pose a significant threat to public health. As a prominent instance, the severe acute respiratory syndrome coronavirus 2 (SARS-CoV-2) that caused the COVID-19 pandemic has affected the world in an unprecedented manner over the past few years. Despite the dissipating pandemic gloom, the lessons we have learned in dealing with pathogen-laden aerosols should be thoroughly reviewed because the airborne transmission risk may have been grossly underestimated. From a bioanalytical chemistry perspective, on-site airborne pathogen detection can be an effective non-pharmaceutic intervention (NPI) strategy, with on-site airborne pathogen detection and early-stage infection risk evaluation reducing the spread of disease and enabling life-saving decisions to be made. In light of this, we summarize the recent advances in highly efficient pathogen-laden aerosol sampling approaches, bioanalytical sensing technologies, and the prospects for airborne pathogen exposure measurement and evidence-based transmission interventions. We also discuss open challenges facing general bioaerosols detection, such as handling complex aerosol samples, improving sensitivity for airborne pathogen quantification, and establishing a risk assessment system with high spatiotemporal resolution for mitigating airborne transmission risks. This review provides a multidisciplinary outlook for future opportunities to improve the on-site airborne pathogen detection techniques, thereby enhancing the preparedness for more on-site bioaerosols measurement scenarios, such as monitoring high-risk pathogens on airplanes, weaponized pathogen aerosols, influenza variants at the workplace, and pollutant correlated with sick building syndromes.

## Abstract 2

The framework for on-site airborne pathogen detection and transmission risk mitigation.

## Graphical abstract

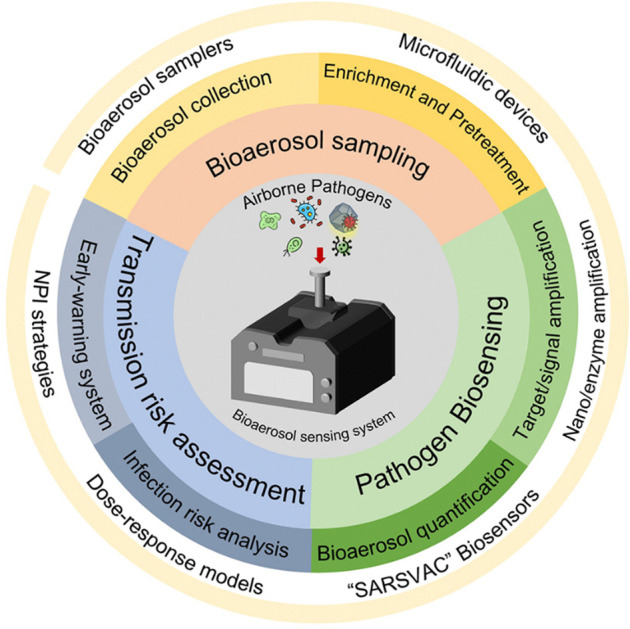

## 1 Introduction

**Fig. 1:**
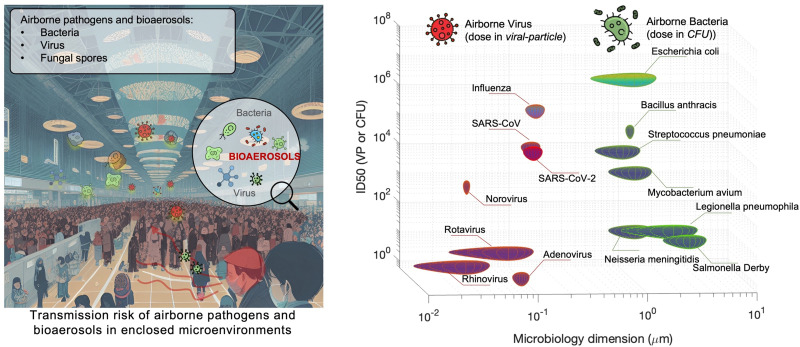
Schematic illustration of airborne pathogens emission and transmission risk in enclosed indoor environments. The figure on the right shows the typical pathogens that may be airborne transmitted, including bacteria and viruses, as well as their physical dimension and the infection dose (ID_50_). VP, virus particle; CFU, colony-forming units; ID_50_, median infective dose.

Although there are still many controversies and unknowns about how respiratory pathogens spread between hosts, we must acknowledge that the recent outbreaks of COVID-19 pandemic has revealed critical knowledge gaps in understanding the airborne pathogen transmission: bioaerosols could be much more prevalent than previously recognized and may be one of the dominant routes for infection disease spreading.^1–3^ For instance, respiratory viruses, such as the severe acute respiratory syndrome coronavirus (SARS-CoV), Middle East respiratory syndrome coronavirus (MERS-CoV), seasonal influenza virus, and respiratory syncytial virus (RSV), and bacteria, such as *Escherichia coli*, *Salmonella enterica* species, *Enterococci* species, *Cryptosporidium parvum*, and *Campylobacter*, could cause infections by inhaling pathogen-laden aerosols (Fig. 1 and Table S1, ESI†).^4–8^ There are also robust evidences supporting that the airborne transmission of SARS-CoV-2 contributed significantly to the recent COVID-19 pandemic.^9–11^ Despite the broad consensus that individual infection screening can mitigate pathogen spreading, airborne pathogens transmissions and infections cannot be fully eliminated in crowded indoor environments, such as hospitals and nursing homes, due to the large number of asymptomatic infections.^12,13^ Moreover, when airborne pathogens are weaponized, the transmission risks of disease can be manipulated to place a biosafety threat. In response, many research and practical efforts have focused on achieving on-site airborne pathogen detection and fast infection transmission risk estimation.^14–22^ Of particular interest in this context are enclosed indoor environments, such as schools, hospitals, and public transportation.^9,23–33^

There has been renewed focus and interest in creating tools for the on-site sampling and biosensing of airborne pathogens ever since the recent pandemic. To truly realize the on-site and accurate determination of trace amounts of airborne pathogen, it is critical to review and assess the advanced strategies and novel development in bioaerosol sampling, on-site airborne pathogen detection, and early infection risk assessment. Therefore, in this review, we aim to provide a comprehensive analysis of recent progress in pathogen-laden aerosols sampling and biosensing strategies, and the contributions to on-site infection risk assessment, with particular interests in bioanalytical chemistry innovations (Fig. 1). Most of the current research, especially in healthcare settings, use off-site approaches (such as reverse-transcription PCR) to evaluate airborne pathogen exposure.^11,34^ To engender improvements in this area, we emphasize that on-site measurement with biosensors could benefit transmission control and the implementation of NPI strategies. Moreover, investigating dose–response relations between the exposure level and the infection possibility is critical in determining the required detection limits and correlating measurement results with potential infection risks. Accordingly, dose–response analyses of airborne coronavirus through meta-analysis, animal-based infection experiments, and case studies of SARS-CoV-2 are reviewed and discussed as the typical representative in this work. These research results, including the exposure measurement and dose–response analysis, are the critical basis for conducting risk assessments and determining safety levels.^35,36^

**Fig. 2:**
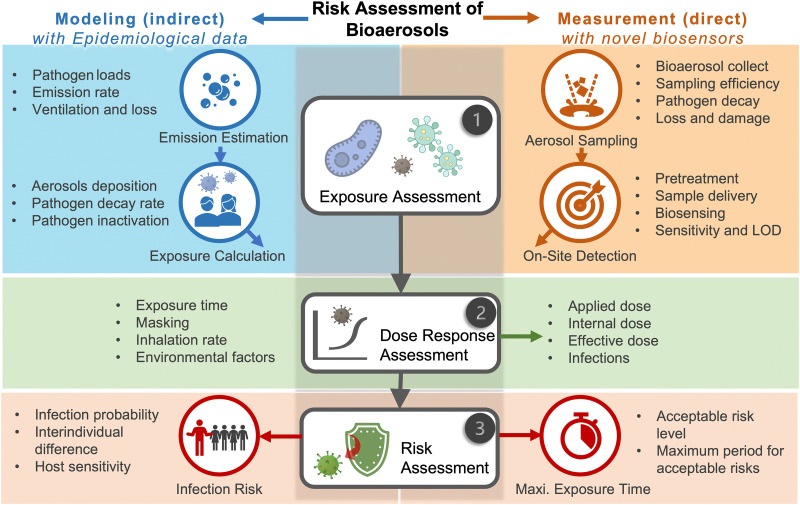
Schematic illustration of on-site risk assessment of airborne pathogens, which includes an indirect approach of using modelling-based emission and exposure estimation with the basis of epidemiological data (left-hand side and the blue panel) and a direct approach of using pathogen-laden aerosol sampling and point-of-exposure measurement (right-hand side and the orange panel). Based on further analysis of human dose–response toward a specific type of pathogens, the risk level, including the personal infection risk and maximum exposure time, can be determined.

Due to the lack of high-resolution spatiotemporal exposure information on airborne pathogens, the current risk assessment strategies for individuals and specific indoor environments are based on extrapolation modeling and theoretical predications.^37^ To this end, on-site airborne pathogen biosensing systems can in principle provide real-time pathogen exposure information with high spatial resolution, as illustrated in Fig. 2. Combined with dose–response relationships and risk level assessments, spatiotemporally accurate risk-alerting systems could be established for implementing more effective epidemic interventions. Such a goal imposes high standards for on-site viral aerosol biosensing technologies.

For on-site pathogen detection, the effective collection of bioaerosols is a critical prerequisite, *i.e.*, with maximal sampling efficiency and minimal damage to bio-functionalities or molecular structures.^38–40^ An ideal bioaerosol sampling system used for on-site airborne pathogen detection should (1) be fast and efficient to handle plenty of air, allowing real-time or quasi-real-time detection, (2) maintain integrity of the sampled pathogen particles, enabling quantitative analysis through biochemical interaction, (3) be easy-to-integrate, enabling rapid subsequent chemical processing and bioanalytical testing. Therefore, we focus our discussion on the development of rapid and bio-integrable pathogen sampling devices, especially those that meet the above requirements.^19^

Similar to bioaerosol sampling devices, many advanced on-site and point-of-care biosensing approaches have been proposed recently.^41–44^ In a manner different to summaries of existing personal diagnosis approaches, we primarily focus on reviewing novel biosensing systems that can rapidly detect trace amounts of airborne pathogen particles on-site. Ideally, on-site biosensing devices should be: (1) Sensitive, with superior limit of detection to quantify trace amount of airborne pathogens; (2) accurate and specific, to provide reliable pathogen exposure information and avoid background interferences from the complex aerosol matrix; (3) robust, so that they can operate on-site in different environments; (4) swift, to provide fast quantitative biochemical analysis results; (5) versatile, allowing the detection of multiple or different pathogen targets under various scenarios; (6) automated, enabling continuous monitoring with minimal human operations; (7) connectable, allowing access to Internet of Things (IoT) for data analysis by edge computation, remote monitoring, telecommunication, and networking. We review and summarize the bioanalytical chemistry technologies that can meet or have the potential to meet the above “*SARSVAC*” criteria. The potential interferences of typical airborne pollutants for on-site pathogen detection as well as the feasible solutions are also discussed. Furthermore, early warning *via* biosensing systems necessitates detection in public areas, where trace amounts of airborne pathogens potentially exist. The last section of this review is dedicated to inspecting novel “plug-and-play” biochemical amplification strategies that help to improve the sensitivity, accuracy, functionality, and robustness of on-site airborne pathogen detection. Last but not least, we share our insights on the open challenges in on-site airborne pathogen detection, such as handling complex aerosol samples, improving the sensitivity for detecting trace amounts of airborne pathogen, and establishing high-spatiotemporal risk assessment systems for mitigating airborne pathogen transmission risk. We hope our cross-disciplinary outlooks in this review could provide inspiration for researchers from different research communities so as to further improve the preparedness for on-site airborne pathogen quantification and risk assessment.

## 2 Airborne transmission of contagious pathogens

### 2.1 Retrospect of airborne pathogens

Dating back to the 1930s, airborne-based infections and transmissions have been classified into two distinct forms, mainly depending on the size of the airborne pathogen carriers.^45,46^ One of them was a “droplet”-based infection, which refers to large respiratory droplets that are rapidly removed from the air by gravity before drying. These droplets generally settle on the surface of objects and indirectly infect susceptible individual (infectee). The second form was introduced as “aerosol”-transmitted infection. Generally, the term “*aerosols*” represents the dried residues or droplet nuclei suspended in the air. Since the sizes of aerosols are typically small (<5 μm), their transmission depends dominantly on the buoyancy of the air, allowing them to remain transporting for extended periods of time and longer distances compared to the droplets. Airborne transmissible pathogens could be directly released from an infected person (infector) and transmitted to an infectee *via* these two airborne-based infection routes (*i.e.*, droplet and aerosol). Traditionally, it is widely believed that direct contact, large respiratory droplets, and droplet-contaminated surface (fomite) are the dominant transmission approaches for respiratory viruses, while long-distance airborne transmission (aerosol) only occurred in special circumstances and for specific pathogens. Fig. 1 summarizes the pathogens that have been found to be transmitted *via* aerosols. For example, the fecal coliform group bacteria such as *Escherichia coli*, *Salmonella enterica* species, *Enterococci* species, *Cryptosporidium parvum*, and *Campylobacter* species can transmit in air and infect others through the aerosolization of contaminated wastewater. Meanwhile, fungal bioaerosols, which consist of spores, mycelium fragments, and debris, can be inhaled and cause numerous symptoms including allergies, irritation, and opportunistic infections.^47^ There are also a number of studies demonstrating the aerosol transmission of respiratory viruses, including influenza, rhinovirus, adenovirus, measles virus, respiratory syncytial virus (RSV), and human coronavirus (*e.g.*, MERS-CoV and SARS-CoV).^5–7,48,49^ For instance, a large community outbreak of SARS-CoV in Hong Kong was investigated by applying airflow dynamic modelling, in which the infection data and the predicted 3D spread results were well correlated.^7^ This study highlighted the importance of considering the aerosol transmission route in infection prevention and control. In addition to respiratory infections and epidemic diseases, bioaerosols can impact the quality of the air we breathe, thereby affecting our daily well-being. The inhalation of bioaerosols could cause irritation and inflammation in the respiratory system. Consequently, sick building syndrome (SBS), characterized by coughing, wheezing, shortness of breath, headaches, fatigue, and decreased cognitive function, may occur in enclosed indoor environments.^50^

In the past few years, the characteristics of bioaerosol transmission have been extensively studied, and many characteristics of the airborne pathogen transmission have been clearly elucidated. Pathogens such as SARS-CoV-2 can be transmitted in aerosols in different environmental scenarios such as health-care settings, private cars, public transportation, schools, bars, and gymnasiums.^11,34,51,52^ Although long-distance transmission and potential high infectivity have been recognized, there are still some controversies about the airborne pathogen transmission, largely because of an incomprehensive understanding of the pathogen-laden aerosols and insufficient reliable analytical chemistry techniques in the toolbox for on-site bioaerosol detection.^28,53–55^ Currently, the accumulated research results not only illuminate the limitation of conventional point-of-views about airborne virus transmission but also bridge the cognitive gap and serve as important knowledge supports for the emerging research regarding on-site bioaerosol sampling, transduction, and risk level assessment. By systematically sorting out the characteristics of aerosol transmission in the following sections, we are aiming to identify the internal physiochemical properties of pathogenetic bioaerosols and the external potential environmental factors that may impact airborne transmission. More importantly, these characteristics can facilitate the development of on-site bioaerosol testing systems for reliable risk assessment and further deepen the epidemiological knowledge about pathogenetic bioaerosol transmission.

### 2.2 Physiochemical properties of pathogen-laden aerosols

Particle size is the most critical factor in determining aerosol behavior. Under typical indoor or enclosed airborne conditions, particles less than 5 microns in size can remain in the air for prolonged time periods.^56^ Airborne bacteria can vary in size depending on the species and typically falls between 0.3 and 60 μm in diameter (Fig. 1). Similarly, airborne fungi generally range from 1 to 100 μm in diameter. Additionally, fungal spores can vary greatly in size depending on the environmental conditions. The size of single viral particles was significantly smaller, with a prevalent diameter of 0.02–0.4 μm.

**Fig. 3:**
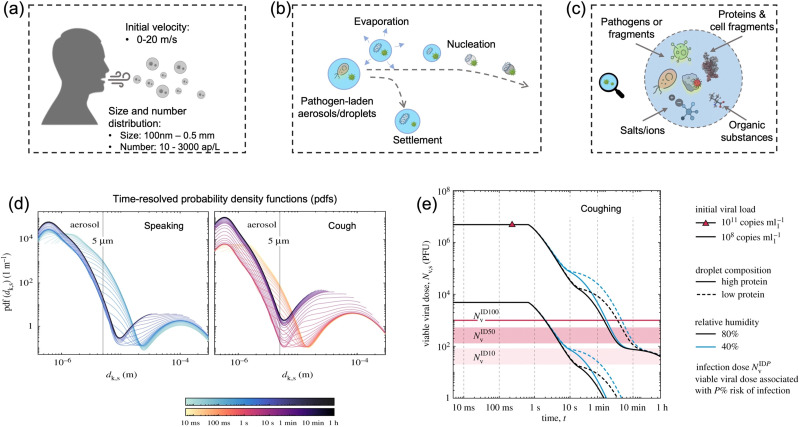
(a) Pathogen-laden aerosols/droplets (100 nm to 0.5 mm in diameter) generated by an infected individuals through respiratory activities such as coughing, speaking, and sneezing. The number concentration of the exhaled pathogen-laden particles is about 10–3000 aerosol particles per liter in air (ap per L). Additionally, the initial velocity could reach up to 20 m s^−1^ and transport to a long distance within a short period. (b) The produced bioaerosols could evaporate to form small aerosols that can persist in the air for a prolonged time. Nucleation refers to the process of forming smaller aerosol nucleus. (c) The chemical compositions of bioaerosols: non-volatile ions, proteins, surfactants, organic substances, cell-fragments, as well as pathogen targets such as bacteria and viruses (or its fragments). (d) Evolution of the probability density function (pdf) of bioaerosols and airborne pathogens from speaking and coughing. These two figures compared the detailed time-resolved pdfs in the range of 0–1 h under a given environmental condition: *T* = 20 °C, RH = 60%. The time scales were colored in a log scale. (e) Evolution of the suspended airborne SARS-CoV-2 doses after a single coughing at 0 s. The impacts of initial viral load, chemical (protein) compositions, and external environments (relative humidity) were compared. Reprinted with permission from ref. 67. Copyright (2021) the Royal Society.

For transmittable pathogenic bioaerosols that cause infections in the respiratory tract, expiratory activities such as breathing, speaking, coughing, sneezing, and singing could be one of the initial sources (Fig. 3a).^10,57,58^ The respiratory airflow and induced shear forces are among the main drivers of respiratory aerosols and droplet formation.^59,60^ For instance, pathogen-laden aerosols can be generated through fluid film rupture in respiratory bronchioles and the resulting aerosols being drawn into the alveoli and discharged during exhalation.^61^ Compared with aerosols generated from the lower respiratory tract, laryngeal and oral aerosols have a broader particle size distribution and primarily contain larger-sized droplets. In case of speech, the exhalation aerosol size from human bronchioles, larynx, and mouth were identified with count-median diameters at 1.6, 2.5, and 145 μm, respectively, while in the case of coughing, the median diameters were located at 1.6, 1.7, and 123 μm.^59^ Nonetheless, the pathogen load is not proportional to bioaerosol size. Recent studies have shown that smaller aerosols (≤5 μm) generated in the lower respiratory tract were likely to contain more respiratory viruses than larger aerosols (>5 μm) generated in the upper respiratory tract.^62,63^

The number concentration of airborne pathogen is another key physiochemical characteristic that should be considered for disease transmission control. However, the number concentration of exhaled pathogen-laden aerosols have large interindividual variability by almost three orders of magnitude and heavily depend on the disease stage, age, and preexisting health conditions.^64^ One recent work suggested that post-symptomatic individuals with coronavirus infection produced higher number of exhaled aerosols at about 1300–2700 aerosol particles per liter in air (ap per L), while the noninfected control and recovered individuals expelled aerosols with much less number concentrations at 7–198 ap per L.^64^ Other expiratory activities such as speaking, coughing, and sneezing can produce even higher number of airborne particles. For instance, loud speech can emit more than 1000 oral airborne particles per s, which refers to a number concentration greater than 2600 ap per L (under an exhalation rate of 1.38 m^3^ h^−1^).^10^

In case of the bioaerosol dimension and number concentration, it is important to consider that the size distribution of pathogen-laden aerosols could evolve over time due to the in-air evaporation, fusion, condensation, and deposition.^65^ Evaporation can decrease the diameter of airborne particles, as shown in Fig. 3b. Additionally, smaller bioaerosols could reach an extremely low settling velocity by reaching a balance between the gravitational force and drag force (Stokes’ Law) and therefore remain in the air for prolonged time periods.^66^ For instance, a 100 μm bioaerosol at a height of 1.5 m can remain in the air for just 5 s, while a smaller bioaerosol with 1 μm diameter can be suspended for more than 12 h.^66^ One recent time-resolved modelling study demonstrated that, as shown in Fig. 3d, all large respiratory droplets (100 μm–1 mm) reach the ground within 1 min, while small aerosols <5 μm could remain suspended after 1 h under a typical ambient condition (temperature *T* = 20 °C and RH = 60%).^67^ Other intrinsic physical properties, such as the initial velocity and the aerosol morphology, also directly determine the transport distance and behaviors in air, as shown in Fig. 3e. For instance, the initial velocity of virus-laden aerosols produced in sneezing can reach 10–20 m s^−1^, thereby spreading the virus over a significant distance.^68,69^

The alterations of physiochemical properties during the generation and transportation of bioaerosols can also impact the viability and infectivity of airborne pathogens.^70^ The initial airborne droplets or aerosols could have similar chemical compositions as the respiratory fluid, which includes non-volatile ions, proteins, surfactants, and organic substances, as shown in Fig. 3c. In-air evaporation and nucleation can elevate salt concentration within the bioaerosols. This concentration-difference between the pathogen interior and the external medium can induce osmotic pressure, leading to changes in the viability of the virus, bacteria, and spores including damage to structural protein, destruction of the overall structure, and degradation of internal nucleic acids.^71^ Therefore, the physicochemical impacts on the viability of airborne pathogens should be considered when using the on-site measurement results to assess the transmission risk. Section 2.4 will discuss the external environmental factors that could potentially impact the viability of airborne pathogen and the quantitative on-site detection results.

### 2.3 Airborne transmission route of contagious pathogens

Airborne pathogen can originate from a variety of sources, including infected individuals, environmental sources, and heating ventilation and air conditioning (HVAC) systems. Among these, the inhalation of respiratory bioaerosols containing the pathogen is the most prevalent and dominant transmission route. Previous studies have demonstrated that airborne pathogens such as tuberculosis, measles, chickenpox, and influenza can be transmitted *via* air through inhalation. Typical scenarios and case studies of airborne pathogen transmission, including virus, bacteria, and fungi, are summarized in Table S2 (ESI†).

Ventilation is an effective means of addressing and reducing the indoor airborne transmission of infectious pathogens. Increased airflow can effectively reduce the concentration of bioaerosols in enclosed spaces. However, external airflow such as HVAC systems may facilitate the diffuse and disperse of airborne particles in the environment, particularly fine pathogen-containing aerosols. For example, a cluster of SARS-CoV-2 infections was reported among three families seated at adjacent restaurant tables.^72^ Reportedly, the HVAC system also contributed to the outbreak of Legionnaires' disease in different indoor environments.^73,74^ In these cases, the air-conditioner was suspected to be the main driving factor to promote the aerosol transmission and potentially caused the sick-building syndrome.

Additionally, alternative bioaerosol transmission routes based on environmental sources may also widely exist. Pathogens may also be present in the soil, water, or animal waste. These pathogens may become airborne through natural processes such as wind or human activities, such as construction or farming.^75^ Moreover, animal farms have been considered as the critical reservoir of antibiotic resistant bacteria (ARB) and potentially spreading antibiotic resistant gene (ARG) that threaten human and animal health worldwide.^76^

The human digestive system can also excrete infectious pathogens such as *E. coli*, *Clostridium difficile*, influenza, SARS-CoV-2, *Mycobacterium tuberculosis*, and *Streptococcus pneumoniae*. Recent studies have revealed that the amount of SARS-CoV-2 viral genetic materials were found to be 10^2^–10^5^ copies per mL in urine and 10^2^–10^7^ copies per mL in stool samples.^77–79^ One investigation also suggested that a large number of aerosols in the size range 0.3–3 μm were generated during toilet biomatter flushing, and these fine aerosols could remain suspended in air for long periods of time and reach heights of >1.52 m.^77^ A fluidic dynamic simulation also demonstrated that up to 60% of pathogen-laden aerosols can transport be upward above the toilet seat during flushing.^79^ These discoveries also indicated the potential airborne transmission route from sewage networks and wastewater treatment.^80^

Another important aspect that needs to be considered in the airborne transmission route is the lifetime of airborne pathogens, which is dominantly determined by two factors: (1) the airborne lifetime that the bioaerosols remain suspended in air and (2) the inactivation lifetime (pathogen half-life) determined by external environment factors. The airborne lifetime is related to the stabilized aerodynamic size. As mentioned earlier, the suspension duration can be predicted theoretically by Stokes' law, where the terminal velocity of a falling aerosols is approximately proportional to the square of its diameter. In a recent study, the authors utilized a stagnant-air environment to investigate the air-suspension lifetime of aerosols produced by speech. Apart from the time-resolved simulation showed in Fig. 3d, there are a number of direct experimental works to investigate the aerodynamic lifetime of virus-laden aerosols. For instance, one study indicated that the droplet nuclei of 4 μm diameter (corresponding to 12–21 μm droplet prior to dehydration) remained in air for 8–14 min.^10^ Similarly, another recent study also suggested that 10 μm aerosols would take 9 min before reaching the ground when produced at a height of 160 cm.^81^

Regarding the inactivation lifetime of airborne pathogens, the Goldberg-drum approach is typically used to investigate the viability and persistence of the suspended pathogens.^82^ It has been revealed that the median half-life of SARS-CoV-2 was estimated to be about 1.1 h at 65% relative humidity (RH) and 21–23 °C.^83^ In another experimental study, airborne SARS-CoV-2 virus particles retained infectivity and virion integrity for up to 16 h at 53% RH and 23 °C.^84^ The half-life and infectivity of airborne pathogens are predominantly affected by ambient environmental conditions such as RH, temperature, and irradiation conditions.^85^ These key environmental and external factors affecting the airborne transmission of pathogens are discussed in detail in the following section.

### 2.4 Key environmental factors affecting the transmission of airborne pathogens

#### 2.4.1 Temperature

**Fig. 4:**
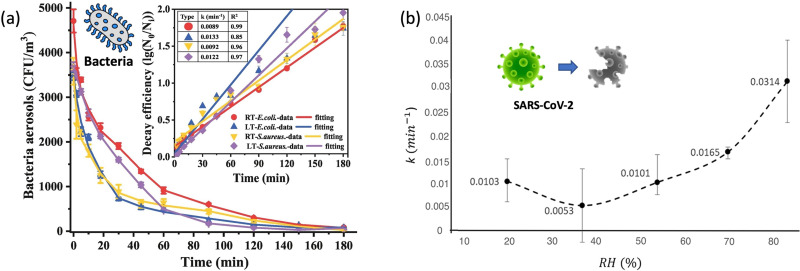
(a). Self-decay kinetics laws and efficiency fitting model of airborne *E. coli* and *S. aureus* under room temperature (18 ± 2 °C, RT) and low temperature (3 ± 2 °C, LT) conditions. Reprinted with permission from ref. 87. Copyright (2022) Elsevier B.V. (b). The inactivation rates or the biological decay constant (k) for enveloped coronavirus (*e.g.*, SARS-CoV-2) at different RHs from 20% to 90%. Reprinted with permission from ref. 93. Copyright (2021) Elsevier B.V.

Temperature is significant in maintaining the viability of the airborne pathogens in terms of biomolecular morphology, structure, and function.^86^ Therefore, airborne transmissibility can be affected by ambient temperature in many different aspects such as temperature-dependent infectivity and airborne viability. Bacteria have a range of optimal temperatures for growth, and any deviation from that range can negatively affect their viability. One recent study investigated self-decay laws and efficiencies of airborne bacteria under different temperatures.^87^ As illustrated in Fig. 4a, the Gram-negative bacteria were found to be more sensitive to temperature change compared with Gram-positive bacteria, where the self-decay efficiency of Gram-negative under low temperature (3 ± 2 °C) was 49% higher than that under room temperature (18 ± 2 °C), and the value of Gram-positive was 32% at the same condition. Regarding the airborne virus, recent study revealed that enveloped viruses such as SARS-CoV-2 decayed more rapidly when either humidity or temperature was increased.^88^ At a typical room temperature of 24 °C, the half-life of the virus ranged from 6.3 to 18.6 h but significantly decreased to 1.0 to 8.9 h when the temperature increased to 35 °C. The thermodynamic nature of biomolecules may decay faster at elevated temperatures, thereby making pathogens lose their viability and infectibility. Therefore, airborne pathogens such as coronavirus and influenza virus are more persistent at lower temperatures.

The second aspect affecting airborne pathogen transmission is the infectivity under different ambient temperatures. During expiration, the temperature along the respiratory tract progressively decreases with airflow from the lower to the upper part.^89^ Specifically, the average ambient temperature of the lower respiratory tract is approximately 35–37 °C under normal quiet breathing conditions and slightly decreases to about 32–33 °C in the upper trachea and mouth. By an increase in the breathing rate, the temperature along the airway may slightly fell to 33.9 °C and 29.2 °C, respectively. A recent study has shown that the infectious titer and replication kinetics were higher at lower temperature of 33 °C than that at 37 °C, which indicated that respiratory viruses like SARS-CoV-2 had a better replication capacity in the upper respiratory tract.^90^ Therefore, colder incubation temperature, such as the elevated breathing rate and lower environmental temperature, allowed the virus to replicate faster, therefore facilitating the transmission of the airborne viruses.

#### 2.4.2 Humidity

Typically, pathogen-laden aerosols expelled from respiratory tract lose both heat and moisture, thereby quickly forming smaller equilibrium particles within seconds (Fig. 4b). The evaporation and the induced osmotic pressure may significantly impact the viability of the pathogen by disrupting the biomolecular structures. By comparing the infectivity at different RHs, it was found that the osmotic stress and salts crystallization at low RH conditions led to an instant loss of infectivity of more than half of the airborne virus.^91^ In contrast, at a high RHs of 80% and above, the airborne virus might be far more stable, with infectivity rarely falling below 80% after 2 min. Regarding the long-term infectivity, a decrease to ∼10% of the starting value was observable for both RH = 40% and 90% over 20 min. In addition, different chemical components in the original aerosols or droplets may also affect the stability of airborne pathogens when changing the RH. An acidic environment may decrease the pathogenic viability, while organic macromolecule contents (protein, surfactants) could enhance the longevity of airborne viruses.^91,92^ Based on the experiments results from the literature, Aganovic and co-workers summarized the biological decay constant (*k*) at different RHs from 20% to 90% at a constant temperature of 20 °C, as shown in Fig. 4b.^93^ The airborne enveloped viruses demonstrated a high viability and a low decay rate at RH = 37%. To date, the mechanism and impact of RH-induced inactivation are still not fully understood. The efflorescence-deliquescence divergent infectivity (EDDI) hypothesis was recently used to predict the RH-dependent survival of airborne virus, which suggested that the surviving fraction in low RH was higher than that in higher RH aerosols.^94^ This trend was also consistent with the collective experimental results shown in Fig. 3e and 4b.

Notably, enveloped airborne viruses such as influenza and coronavirus follow this trend and survive longer at lower RHs, whereas non-enveloped airborne viruses have a tendency to survive longer at higher RHs. Similarly, both Gram-positive and Gram-negative bacteria have an optimal RH range between 40% and 60% for their survival. High RH levels can cause the cell wall to rupture, leading to bacterial death. On the other hand, low RH levels can cause the cell wall to dehydrate, leading to a decrease in bacterial growth and reproduction. Therefore, it is important to consider both the pathogen species and its behaviors under different RHs when assessing the transmission risk of airborne pathogens.

#### 2.4.3 Radiation conditions

Radiations including the UV light can rapidly inactivate pathogens in aerosols.^85,95^ In airborne transmission, bioaerosols are inevitably exposed to different irradiations such as natural sunlight, especially in outdoor environments. Using the solar simulator with spectra designed to represent the UV range (280–400 nm) of natural sunlight, the decay rate and lifetime of airborne bacteria and viruses were investigated in a Goldberg rotating drum aerosol chamber.^95^ For enveloped viruses such as influenza and SARS-CoV-2, the mean decay rate in simulated saliva were found to be 0.121 ± 0.017 min^−1^ (90% loss, 19 min) under winter sunlight condition and 0.306 ± 0.097 min^−1^ (90% loss, 8 min) under summer sunlight condition. The mean decay rate without simulated sunlight was much lower at 0.008 ± 0.011 min^−1^ (90% loss, 286 min). In addition, prolonged exposure to visible light of short wavelengths (400–420 nm) may also reduce the viability of airborne pathogens.^96^ Additionally, when applying an average UV intensity of 10 W cm^−2^, 63 percent of airborne tuberculosis bacteria could be inactivated in 24 s, and 99 percent were eliminated in 2 min. Another study demonstrated that the median natural inactivation ratio of airborne *Mycobacterium abscessus* was 62.2% after 30 min and 75.5% after 40 min.^97^ An increased UV-c radiation dose by 83.1 μW s cm^−2^ can elevate the ratio to 83.1%.

Therefore, the further evaluation of the surrounding UV (radiation) exposure levels is recommended in order to accurately calculate the viability and actual transmission risk of pathogens. Although ordinary indoor lighting systems, such as white LED and Halogen lamp, barely contain radiation in the UV band, the decay rate of the airborne pathogens during on-site risk assessment should also be further investigated and considered.

#### 2.4.4 Ozone and airborne oxidizing agents

The inactivation of airborne pathogens by ozone and other oxidizing agents is another significant factor that needs to be considered in evaluating the infection and transmission risk.^98,99^ The general inactivation mechanism is based on chemical reactions between the oxidants and the biomolecules constituting the essential structures or functions of pathogens.^100^ For instance, ozone or other oxidant species, *e.g.*, hydroxyl radical, singlet oxygen can react with the nucleic acids of the airborne pathogens and potentially impact the accuracy of biomolecular sensing results. The recent experimental results demonstrated that the longevity of the enveloped coronavirus was negatively impacted by ozone.^98^ The spread of airborne viruses was obviously reduced by three order of magnitudes by increasing the ambient ozone concentration level from 48.83 to 94.67 μg m^−3^. Similar to effective UV treatment, high oxidant concentrations (129 000 μg m^−3^ min) may inactivate most of the airborne viruses within minutes.^99,101^

The key environmental factors mentioned above can individually impact the transmissibility and infectivity of airborne pathogens. However, the overall impact on the airborne transmission should be carefully considered based on the specific on-site environments. For instance, although both higher RHs and UV radiation can inactivate airborne enveloped virus, the elevated RH may also reduce the UV damage and thereby increase their persistence.^102,103^ Therefore, in the on-site bioaerosol sampling and sensing process, it is necessary to fully consider the inactivation and decay rate caused by different environmental factors so as to accurately calculate the concentration and quantitative infection risk level of viable pathogens.

### 2.5 Growing concerns over airborne pathogens

Over the past few decades, airborne pathogens and bioaerosols have been a growing public health concern due to their potential to cause widespread disease outbreaks, their use as biological weapons, and daily health impact such as sick-building syndrome.

**Fig. 5:**
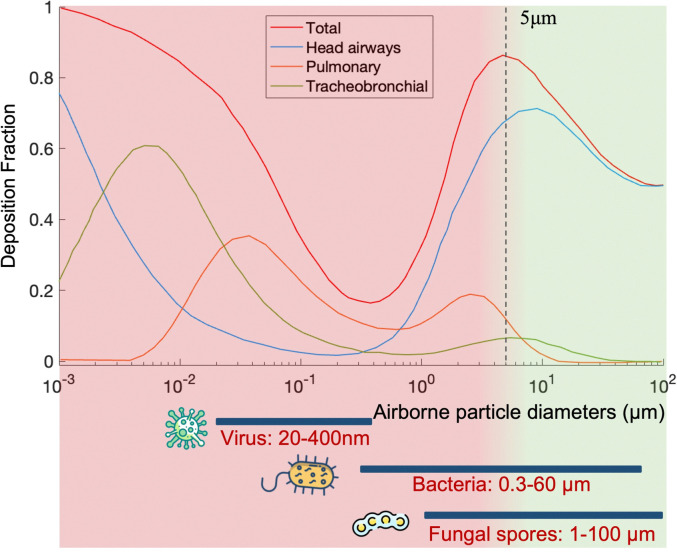
Demonstration of the typical dimensions of different airborne pathogens and their potential deposition locations *via* breathing, including the whole respiratory tract (total), nose-nasopharynx-larynx (head airways), tracheobronchial airways, and alveolar region (pulmonary). The depositions were predicted based on the mathematical model (multiple-path particle dosimetry model, applied research associates).

One of the main concerns is the airborne transmission of contagious pathogens. Based on the physiochemical properties of bioaerosols introduced in Section 2.3, airborne pathogens transport and deposition in the respiratory airways can be determined using several established models such as the multiple-path particle dosimetry (MPPD) model, as shown in Fig. 5.

Regarding normal respiratory activities, large droplets mainly transport for a short distance in the air (usually less than 1 m), but they deposit on the lip/eye/nostril mucosa of another person at close contact and cause inoculation. In contrast, pathogen bioaerosols smaller than 2.5 μm can remain suspended in the air for a long time and penetrate deeply into the lower respiratory tract to cause infections. One example is *Streptococcus pneumoniae*, a 0.5–1.2 μm bacterium that can penetrate into the trachea, bronchi, and cause pneumonia. Another example is *Mycobacterium tuberculosis*, the bacterium that causes tuberculosis. Viruses such as influenza and SARS-CoV-2 can also cause lower respiratory tract infections. Most seasonal Influenza viruses infect the cells lining in the larger airways, trachea, and nasopharynx, while the pneumotropic influenza A virus strains such as H5N1 target the lower respiratory tract like the lung airways and alveoli.^104^ Additionally, SARS-CoV, MERS-CoV, and SARS-CoV-2 also replicate in the lower respiratory tract in humans. By considering the dimension of airborne pathogens, fine (<2.5 μm) and ultrafine (<0.1 μm) bioaerosols have become a common and growing concern due to their large penetration depth and lower inoculum in causing respiratory tract infections. However, current bioanalytical systems for detecting bioaerosols are still not well-established. This has also led to a current technology shortage in the toolbox for epidemic prevention and public health intervention.

Additionally, bioaerosols and airborne pathogens may influence our daily lives in a pervasive but covert manner. The accumulation of airborne microorganisms may result in sick building syndrome (SBS), characterized by asthma symptoms, mucous membrane irritation, gastrointestinal disturbances, and neurotoxic effect. Severe toxicosis and cancer could also result from a long-term and continuous exposure to airborne mycotoxin. At present, the specific conditions that lead to SBS are not fully understood. All of these concerns about airborne pathogens motivate ongoing research into the detection and risk evaluation of bioaerosols.

## 3 Risk level determination of airborne transmission

Based on the progressively improved understanding of airborne pathogen transmission, the importance of the fast and quantitative risk assessment of airborne pathogen transmission for a wide variety of public and indoor environments has been recognized.^36,105,106^ To achieve reliable risk level determination of airborne pathogen transmission, many key factors and aspects need to be considered, such as the airborne pathogen emission, airborne pathway by considering the decay and removal rate, human exposure, and dose–response relationships.^105,107,108^ Using respiratory viruses as examples, this section will review the recent advances and challenges of risk assessment of airborne pathogens based on the extensive and systematic studies conducted in recent years.

### 3.1. Exposure to airborne pathogens

Airborne pathogen exposure is defined as a contact over time and space between a person and bioaerosols. Exposure assessment is to identify and define the exposures that occur or are anticipated to occur within a specific microenvironment. This can be a complex endeavor requiring analysis of many different aspects of the contact such as bioaerosol emission, removal rate, exposure pathway and route, and exposure concentration and duration.

As the central feature of airborne transmission risk assessment, quantitative exposure assessment can be approached in two general ways: direct measurement (*e.g.*, on-site and point-of-contact sensing) and indirect measurement (*e.g.*, modelling). Direct measurements are an unequivocal way to establish individual exposure assessment to a specific airborne pathogen like coronavirus, influenza, or *Bacillus anthracis*. However, the direct measurement of airborne virus exposure in this ongoing COVID-19 remains technically impracticable and unaffordable for broad applications. Thus, using mathematical models and information abstracted from physical realities can be a cost-effective way for the urgent need of extensive exposure assessment and risk level determination. Some representative measurement results (data collected from different epidemiological studies) and preliminary characteristics can be used in the models for exposure estimations. Therefore, the main challenge is to develop appropriate and robust mathematical models so as to extrapolate the exposures and doses of individuals and communities in more different environmental scenarios. In this section, recent advances in bioaerosol exposure and transmission risk assessment will be analyzed using SARS-CoV-2 as a typical case study.

#### 3.1.1 Respiratory pathogen emission

Bioaerosol emission is the fundamental data for the evaluation of the transmission risk. Respiratory pathogens shedding such as coronavirus (NL63, OC43, HKU1, and 229E) and influenza viruses were directly measured in the exhaled breath and coughs of patients with acute respiratory illness.^109^ Results indicated that 10^2^–10^5^ virus particles were emitted and carried by aerosol particles less than 5 μm for 30 min, whereas 10^4^ virus particles were loaded onto particles larger than 5 μm in the same conditions.

Recently, Buonanno and co-workers proposed a forward emission approach to estimate the airborne virus emission based on the viral load in the sputum.^105^ The virus (quanta) emission rate is evaluated asER_q_ = *C*_v_·*C*_i_·IR·*V*_d_ = *C*_v_·(*C*_RNA_·*C*_PFU_)^−1^·IR·*V*_d_in which *C*_v_ is the viral load in the sputum (*i.e.*, 10^7^ RNA copies mL^−1^), *C*_i_ (quanta RNA copies^−1^) is a conversion factor defined as the ratio between one infectious quantum and the infectious dose expressed in viral RNA copies, IR is the inhalation rate (m^3^ h^−1^), and *V*_d_ is the droplet volume concentration expelled by the infectious person (mL m^−3^). In this work, a quantum is defined as a dose of airborne droplet nuclei required to cause infection in 63% of susceptible persons. Based on the retrospective results on human coronavirus, the authors adopted an average *C*_PFU_ value (quanta-to-plaque forming unit conversion factor) of 2.1 × 10^2^ PFU quanta^−1^ and an average C_RNA_ (RNA copies-to-PFU conversion factor) of 1.3 × 10^2^ RNA copies PFU^−1^ to quantify the emission rate.^110,111^

Emission estimation is a convenient but indirect method for predicting the airborne pathogen concentrations. Although some different factors have been considered, the amount of pathogen shedding in patients can fluctuate greatly depending on the personal conditions such as the vaccination status, stage of infection, personal health conditions, and the infected coronavirus strain.^112,113^ Additionally, the majority of respiratory pathogens lack systematic research efforts comparable to those of the novel SARS-CoV-2 virus. Consequently, there are no reliable data to evaluate the exhalation discharge of pathogens at the individual level.

#### 3.1.2 Evaluation of the exposure to airborne pathogens

**Fig. 6:**
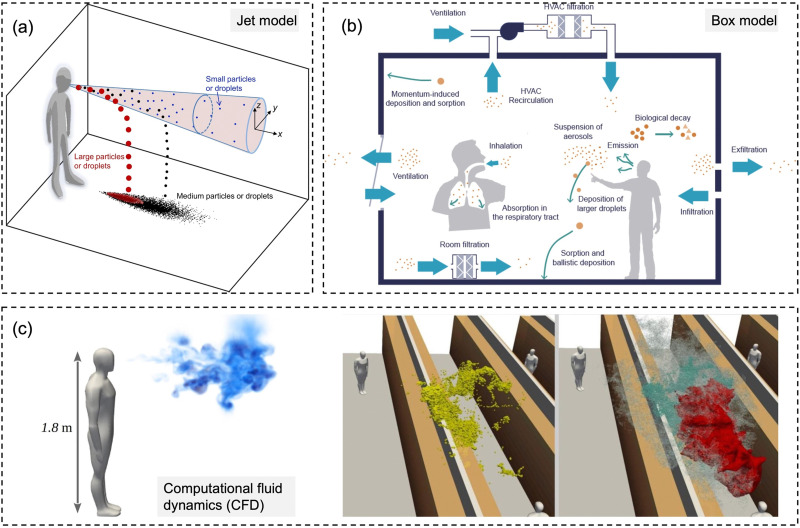
Schematic diagrams for three types of numerical models for modeling-based respiratory bioaerosols exposure assessments. (a) Jet models were utilized to evaluate the exposure of “close contact” by considering both the large droplets and small airborne droplet nuclei in the exhaled plume. Reprinted with permission from ref. 115. Copyright (2015) Elsevier B.V. (b) Box models were generally utilized for estimating the airborne pathogen exposure by assuming well-mixed contaminants and homogeneous concentrations in the enclosure space. Reprinted with permission from ref. 107. Copyright (2021) Elsevier B.V. (c) Computational fluid dynamics (CFD) represents the 3D scale-resolving models, which can calculate the airflow field with high temporal and spatial resolutions. The dispersion of pathogen-laden aerosols can be estimated based on the detailed flow field. Reprinted with permission from ref. 114. Copyright (2020) Elsevier B.V.

There are mainly three types of numerical models to evaluate the exposure to bioaerosols, *i.e.*, jet models, box models, and computational fluid dynamics (CFD) models, as shown in Fig. 6.^107,114^ Jet models are utilized for exposure at a short distance, while the box models are usually for exposure assessment in the enclosure space with the assumption of a well-mixed condition with the homogenous distribution of particles. CFD models represent the detailed physical models for both short- and long-range dispersion.^107^

For the exposure due to close contact, mathematical models for the dynamics of expired jets are normally utilized to evaluate the exposure to large droplets (>5 μm) and small airborne droplet nuclei (<5 μm) in the exhaled plume (Fig. 6a).^115,116^ Turbulent round jet models are usually adopted to describe the velocity profiles in the expired jets, which normally follow Gaussian profiles.^117^ Lagrangian methods, *e.g.*, discrete random walk model, are adopted to estimate the motion of the particles in the jets.^115^ The simplified jets and dispersion model could be utilized to evaluate the exposure over short distances, *e.g.*, several meters, due to the combined impacts of large droplets and small airborne droplet nuclei.

Box models, as shown in Fig. 6b, are adopted as a simplified method to evaluate the concentrations of pathogens in indoor environments, *e.g.*, rooms, cars, and airplanes.^107^ Box models assume well-mixed contaminants and homogeneous concentrations in the enclosure space; thus, the variation of the airborne concentration of active pathogens can be described by an ordinary differential equation based on mass balance, which is solved analytically.^35,107,118,119^ Box modes are dependent on the important parameters, including pathogen shedding rate (emissions), the inner volume of enclosure space, ventilation rate, deposition of particles on the surfaces, and biological decay.^119^ Parameterization models are normally required for the estimation of deposition rates.^120^ Box models could quantify the average exposure in the enclosure space, which is a reasonable assumption when people tend to move around, *e.g.*, in supermarkets and shopping malls.^121^ However, it might have biases when people are relatively static, *e.g.*, in classrooms and libraries, where the spatial distribution of bioaerosols like coronavirus becomes more important, and detailed methods are needed.^122^

Computational fluid dynamics (CFD) models, as shown in Fig. 6c, are detailed physical models with high fidelity and also with high computational demands. The 3D scale-resolving models are able to calculate the airflow field with high spatiotemporal resolutions, and then the dispersion of pathogen aerosols can be estimated based on the detailed flow field. Four different open source CFD softwares, PALM, OpenFOAM, NS3dLab, and fire dynamics simulator (FDS), have been applied to investigate the SARS-CoV-2 exposure levels in generic public places, enclosed/semi-enclosed restaurants, airplanes, negative-pressure wards, as well as outdoor spaces.^114,123–129^ CFD models are also used to investigate the transport of pathogen bioaerosol in the respiratory airways, which could contribute to better exposure assessment.^130^ In the CFD models, the Eulerian–Lagrangian-coupled framework has been normally adopted respectively for flow field (Eulerian method) and particle motion (Lagrangian method). The effects of drag force, gravity, and lift force on particle motion are considered in the Lagrangian method to simulate discrete aerosol particles.

### 3.2 Dose–response analysis of airborne pathogens

The dose–response relationship depicts the possibility of infection or other outcomes of disease given the exposure to a certain number of pathogens including virus and bacteria. It is the indispensable component in risk assessment to link the exposure level and infection risk. The ID_50_, which refer to the estimated number of pathogens required to produce infection in 50% of normal adult humans, is given in Fig. 1. However, there are still significant uncertainties in the dose–response relationship for most airborne pathogens.^131^

#### 3.2.1 Experimental studies based on animal models

Ethical concerns limit the investigation of infection by challenging healthy volunteers, especially for pathogens with high mortality. As a result, animal studies (*e.g.*, mice, ferrets, and monkeys) are usually conducted to investigate the infectious dose. Animal models are developed by transgenic methods to express the human receptor for pathogens and to simulate the infection of humans.

Recently, twelve 10-month-old ferrets and fifty hamsters were inoculated by the intranasal route with a low viral dose of SARS-CoV-2 (2 × 10^3^ PFU for ferrets and 1.8 × 10^3^ PFU for hamsters), which was considered close to the common infection conditions in humans.^132^ Three ferrets had lethargy on days 7 and 8 post-infection. On days 2 and 4 post-infection, viral RNA was found in the trachea from 2 out of 3 ferrets, but no viral RNA was detected in the lung. Viral RNA was observed in the trachea and lung from 3 out of 3 hamsters. The low challenge dose induced consistent lung infection for hamsters but not for ferrets. In another study, six ferrets were intranasally inoculated with a high viral dose of 10^5.5^ TCID_50_ (*i.e.*, the 50% tissue culture infectious dose).^133^ Increased body temperature was observed in all of the ferrets on day 4 after the inoculation, and viral RNA was found in the lungs of all the six ferrets. A high intranasal infection dose might be required to induce lung infection for ferrets. Intratracheal inoculation with 10^6^ TCID_50_ of SARS-CoV-2 (strain BavPat1/2020) caused 100% lung infection of nine ferrets. Similar results were obtained in a study intranasally challenging ferrets with different doses (500, 5 × 10^4^, and 5 × 10^6^ PFU), where viral RNA shedding in the upper respiratory tract was observed in 6/6 ferrets for medium and high doses, but only 1/6 ferrets showed similar signs with low dose (500 PFU).^134^

Most of the available animal experiments were conducted with high infection doses to induce severe clinical infections and nearly 100% infection of all the involved animals. More studies with low doses should be performed to explore the possibility of infection using a similar exposure condition for humans.

#### 3.2.2 Statistical investigation using meta-analysis

In addition to animal studies, the epidemiological investigations, systematic reviews, and meta-analysis shed light on the dose–response relation for humans.^135^ The systematic review provided a summary of medical reports on a specific clinical question, *i.e.*, the infection risk of SARS-CoV-2, using explicit methods to systematically search, critically appraise, and synthesize in the literature.^135^ After the pandemic outbreak, WHO COVID-19 systematic urgent review group effort (SURGE) conducted a systematic review and meta-analysis to evaluate the transmission risk of SARS-CoV-2, and the results indicated that the anticipated probability of viral infection is about 12.8% within 1 m and about 2.6% at a further distance based on the studies of the beta-coronaviruses, which include SARS-CoV and MERS-CoV.^136^ Using the results from meta-analysis, a simple framework was developed to integrate the *a priori* dose–response relation for SARS-CoV based on mice experiments and respiratory virus shedding in exhaled breath to estimate the dose–response relation for humans.^137^

#### 3.2.3 Case-based inference

The dose–response relation could also be estimated based on the case studies. Case studies were utilized to derive the dose–response probability of infection curves for foot-and-mouth disease virus (FMDV) using the infected cases and the estimated exposure rates for the farms from 1967 to 1968 at Oswestry, Shropshire, UK.^138^ The infection risk of the emitted virus-laden particles was inversely estimated based on the data from the Skagit Valley Chorale superspreading event and the Well-Riley risk equation.^33^

### 3.3 Quantitative risk assessment for airborne transmission

#### 3.3.1 Individual risk assessment of airborne pathogens

Adequate exposure models and dose–response models can be used to estimate individual exposures (*e.g.*, the distribution of exposures among members of a population) and group exposures (a population mean). The individual infection risk due to the exposure to airborne pathogens can be evaluated by the combination of exposure assessment and dose–response analysis, which is referred to as risk characterization. We tabulated the quantitative infection risks of airborne SARS-CoV-2 virus for various indoor environments in light of the research works that emerged during the pandemic (Table 1).^105,119,137,139–141^ The quantitative infection risk levels after 1 h exposure in different environments were determined using the exposure model and dose–response model. These infection risks were in the range from 10^−5^ to 10^−2^. Various input parameters, such as the airborne pathogen emission rate, ventilation, room volume, and infection doses, are required for risk characterization. However, there might be significant uncertainties and debatable assumptions in the input data, especially for emerging pathogens.

**Table 1:** Quantitative risk assessment of airborne pathogen (*i.e.*, SARS-CoV-2) with modeling approaches

Scenario	Viral shedding^b^	Exhalation rate	Ventilation rate (ACH)	Room volume (m^3^)	Reported pathogen concentration^a^	Inhalation rate (m^3^ h^−1^)	Infection risk (1 h exposure)	Ref.
Seafood market	1 to 10^3^ PFU per h (3 × 10^2^ to 3 × 10^5^ copies per h)	Resting, oral breathing	0.1	3150	0.05 PFU per m^3^ (15 copies per m^3^)	0.6	2.23 × 10^−5^	126
Hospital room	78 PFU per h, 90% CI: 5 to 1200 PFU per h (10^4^ copies per h, 90% CI: 650 to 1.6 × 10^5^ copies per h)	Resting, oral breathing	0.5	100	2.5 PFU per m^3^ (325 copies per m^3^)	0.49	6 × 10^−3^	110
Gym	525 PFU per h, 90% CI: 34 to 8 × 10^3^ PFU per h (6.8 × 10^4^ copies per h, 90% CI: 4.4 × 10^3^ to 10^6^ copies per h)	Heavy exercise, oral breathing	0.5	300	0.9 PFU per m^3^ (117 copies per m^3^)	3.3	1.4 × 10^−2^	110
Public indoor environments (*e.g.*, restaurant, shop)	1050 PFU per h, 90% CI: 67 to 1.6 × 10^4^ PFU per h (1.37 × 10^5^ copies per h, 90% CI: 8.7 × 10^3^ to 2 × 10^6^ copies per h)	Light exercise (light exercise in restaurant or shop?)	0.5	300	1.9 PFU per m^3^ (247 copies per m^3^)	1.38	1.25 × 10^−2^	110
Conference room	6.7 × 10^3^ PFU per h, 90% CI: 441 to 10^5^ PFU per h (8.7 × 10^5^ copies per h, 90% CI: 5.7 × 10^4^ to 1.3 × 10^7^ copies per h)	Light exercise	0.5	800	4.6 PFU per m^3^ (600 copies per m^3^)	0.54	1.18 × 10^−2^	110
Office room	10^4^ copies per h	Resting, oral breathing	1	300	6 copies per m^3^	0.6	2.3 × 10^−5^	144
Classroom	2000 PFU per h^1^ (2.6 × 10^5^ copies per h)	Light exercise	1.5	900	0.6 PFU per m^3^ (80 copies per m^3^)	0.3	9 × 10^−4^	146
Flight cabin	3000 PFU per h	Resting, oral breathing	25	300	8.4 PFU per m^3^ (1092 copies per m^3^)	0.5	2 × 10^−2^	147
Train^b^	2.8 × 10^5^ copies per h	Resting, oral breathing	7.1	57	644 copies per m^3^	0.5	1 × 10^−2^	148
Trolleybus^b^	7.8 × 10^5^ copies per h	20% speaking	10	100	732 copies per m^3^	0.5	1 × 10^−2^	148

a The conversions factors of 130 copies per PFU and 210 PFU per quanta were utilized if they are not provided in the individual papers.

b The viral shedding was estimated based on a super-emitter at resting with respiratory volume of 7500 cm^3^ min^−1^ and a viral concentration of 0.63 copies per cm^3^.

Probabilistic risk assessment is usually adopted to quantify the uncertainties in the risk assessment and to investigate the sensitivities of the results to the input parameters. One case study for the Wuhan South China Seafood Market integrated the key processes, *e.g.*, pathogen shedding, dispersion, deposition in air, biological decay, lung deposition, and dose–response relation, to assess the infection risk *via* the aerosol route.^119^ Monte Carlo simulations were performed to consider the uncertainties in viral shedding, biological decay, and the dose–response parameters. Viral shedding was assumed to be between 1 and 10^3^ PFU per h, and 4.1 × 10^2^ PFU was adopted as the dose (one quantum) to cause infection in 63% of susceptible people based on the data for the infection of transgenic mice susceptible to SARS-CoV.^110^ Median viral shedding (10^1.5^ PFU per h) was equivalent to about 8 × 10^−2^ quanta per h. A box model was utilized to evaluate the exposure. The results indicated that the inflection risk after 1 h of exposure with one infected person in the market was about 2.23 × 10^−5^ (95% confidence interval: 1.90 × 10^−6^ to 2.34 × 10^−4^). The difference between the upper and lower bounds of the 95% confidence interval of the estimated risk was about 2 orders of magnitude, indicating significant uncertainties in the assessment due to the limited and uncertain information on SARS-CoV-2. Monte Carlo uncertainty analysis suggested that dose–response model, viral shedding, and biological decay contributed about 56.8%, 34.5%, and 8.7%, respectively, to the final uncertainty.

The infection risks in four different typical scenarios, *i.e.*, hospital room, gym, public indoor environments (*e.g.* restaurant and bank), and conference room or auditorium, were investigated.^105^ The median viral shedding rates were assumed to range from the lowest level of 3.7 × 10^−1^ quanta per h from a patient at rest in a hospital room to the highest level of 32 quanta per h from a speaking or singing person in a conference room or auditorium. One quantum was assumed to be 2.1 × 10^2^ PFU. The exposure was estimated by a box model. The median infection risk induced by aerosol transmission with 1 h-exposure was estimated to be from 10^−3^ to 10^−2^. In another study, the magnitude of the estimated median infection risks due to aerosol transmission of SARS-CoV-2 ranged from 10^−5^ to 10^−4^ depending on room sizes and ventilation rates.^137^ A detailed theoretical model estimated the risk for indoor airborne transmission as about 10^−3^ to 10^−2^ with 1 h exposure and without a mask.^142^

Quantitative infection risk assessment based on detailed exposure evaluation using computational fluid dynamics methods was also conducted. It was estimated that the infection risk was from 10^−2^ to 10^−1^ for 1 h exposure in typical indoor scenarios with superspreading events, including a call center in Korea and buses in Ningbo and Hunan, China.^125^ Wells–Riley model is another widely used method for the risk assessment of airborne pathogens. The Wells–Riley equation was originally developed for the epidemiological study on a measles outbreak.^143^ The Wells–Riley model directly combines the stable state from the box model and the one-hit exponential dose–response model, *e.g.*, the dose–response model developed for SARS-CoV.^110^ The comparison between Wells–Riley model and the CFD risk assessment has been conducted for indoor spaces.^144^

The various risk levels obtained in different studies were mainly caused by the diverse adopted viral shedding rates and dose–response relations, which suggests that these two factors are the dominant contributors to the uncertainties of modelling-based risk assessment.

#### 3.3.2 Community risk assessment of airborne pathogens

The susceptible-exposed-infectious-recovered (SEIR) model has been widely utilized to investigate the community infection risk.^145^ In the SEIR model, the total (initial) population is categorized into four classes, *i.e.*, susceptible, exposed, infected-infectious, and recovered, and the number of population in each class evolves with time. The SEIR models have been adopted to evaluate the effectiveness of different intervention measures.^146,147^ The probability of disease transmission per contact is a key parameter in the SEIR model. The exposure assessment and dose–response relations could be applied to better quantify the probability and improve the performance of SEIR models for community risk assessment.

Modeling-based indirect risk assessment of airborne pathogen transmission has some apparent disadvantages that need to be considered. One drawback is that it relies heavily on assumptions, which may not always accurately reflect the real-world conditions. The models are based on limited data and may not capture all the complexities of the transmission dynamics. Another disadvantage is that the models may not account for individual variability in susceptibility and behavior, which can have a significant impact on the transmission risk.

The direct risk exposure quantification approaches by leveraging on-site biosensing device is an essential and promising tool for infection risk assessment because it allows for a more accurate and precise estimation of the likelihood of pathogen transmission and infection. This method involves quantifying the number of contagious pathogens present in a given ambient environment as well as the duration and intensity of exposure. By taking into account these factors, on-site airborne pathogen detection and direct bioaerosol exposure quantification method can provide a more realistic assessment for airborne pathogen transmission than other methods that rely solely on qualitative observations or assumptions.

In the following three sections, we will focus on introducing the emerging on-site bioaerosols sensing technologies, which will allow for targeted and more effective interventions to reduce the risk of airborne pathogen transmission.

## 4 On-site airborne pathogens sampling strategies

On-site bioaerosols detection with high spatiotemporal resolution can be a more direct approach to characterize the transmission of airborne pathogens. Notably, an efficient and fast airborne pathogen sampling strategy is the first and crucial step. The bioaerosols should be collected at the interface between the exposure medium (indoor air) and the individual (the breathing zone). The aerosol properties, such as the aerodynamic size and pathogen decay, as well as the impact of specific sampling approaches including loss and damages should be fully considered. For instance, a very wide size range (0–100 μm) of pathogen-laden aerosols can be inhaled and deposit onto different sites of the respiratory tract, as shown in Fig. 5.^148^ Bioaerosols larger than 5 μm are mainly deposited in the nasopharyngeal region, while smaller aerosols could penetrate deeper into the lungs and deposit in the bronchiolar and alveolar regions.^149^ Moreover, Brownian motion further facilitates the diffusion of nanoscale aerosols or single virus particles (*ca.* 100 nm) and leads to high infectivity in the lower respiratory tract. Therefore, the aerosol sampling devices should have high collection efficiency in a wide size range to avoid the underestimation of airborne infection risk. In addition, there may be structural and biomolecular damages of airborne pathogens during the sampling process. This may lead to inappropriate results in subsequent quantitative analysis. In this section, we will fully evaluate these aspects in rapid airborne pathogen sampling and demonstrate recent advances that benefit effective on-site airborne pathogen quantification.

Ideally, the criteria for selecting an optimal aerosol sampling system for on-site bioaerosol quantification depends on following factors: (1) be fast and highly efficient, (2) cause less damage to the recognition biomolecules (proteins or nucleic acids), and (3) be easy-to-integrate for rapid subsequent bioanalytical testing. We summarize the existing mainstream strategies of pathogen-laden aerosols sampling and provide insights into the advantages, potentials, and critical issues of different technical routines. Regarding the three main technical requirements of the “ideal bioaerosol-sampler”, we also review some emerging technologies proposed in recent years. Meanwhile, the potential strategies to eliminate the pathogen loss and damage during the sampling process are also discussed to further benefit the future development.

### 4.1 Solid-phase bioaerosol sampling approaches

#### 4.1.1 Filter-based sampling approaches

**Fig. 7:**
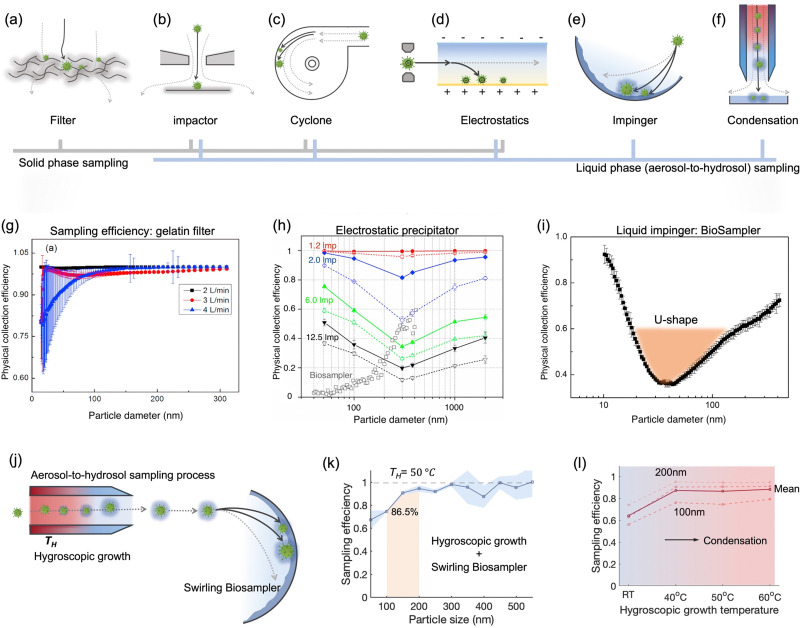
Schematic illustration of common bioaerosols sampling strategies employing (a) filter, (b) impactor, (c) cyclone, (d) electrostatic precipitator, and (e) impinger. (f) Condensation is considered as an effective strategy to enlarge aerosols and improve the sampling efficiency for nanoscale aerosols. The physical collection efficiency of (g) the gelatin filter (with airflow rate varied form 2 L min^−1^ to 4 L min^−1^), (h) electrostatic precipitator with airflow rate changed from 1.3 L min^−1^ to 12.5 L min^−1^, and (i) liquid impinger (BioSampler, commercially available product at SKC, Inc.) with constant airflow rate at 12.5 L min^−1^. (j) An integrated aerosol-to-hydrosol sampling approach with hygroscopic growth-assisted impinger for achieving high sampling efficiency for submicron-scale bioaerosols. (k) Improved aerosol sampling efficiency for nanoscale aerosol particles of 50–550 nm at elevated hygroscopic growth temperature of 50 °C. (l) Sampling efficiency at different hygroscopic growth heating temperatures, *i.e.*, 40, 50, and 60 °C. The solid curve indicated the mean sampling efficiency in the range of 100–200 nm; the dash-curves represent the specific efficiencies for 100, 150, and 200 nm particles. (g) and (i): reprinted with permission from ref. 155. Copyright (2018) Elsevier B.V. Figure (h): reprinted with permission from ref. 171. Copyright (2016) American Chemical Society. (j)–(l): reprinted with permission from ref. 20. Copyright (2022) Wiley.

Filtration is one of the most effective, robust, and commonly used strategies for airborne pathogens collection (Fig. 7a). Through the rational design of the filtration strategies, high sampling efficiency (>99%) within a wide aerodynamic size range (0.1–100 μm) can be achieved. Filter-based methods using PTFE filter and gelatin filter have been widely used in collecting airborne pathogens including virus and bacteria in different environmental settings.^11,150–152^ Particularly, the airflow rate is one of the important characteristics for on-site pathogen-laden aerosol collection. High airflow rate combined with superior sampling efficiency could load more airborne viruses onto the filter, therefore facilitating swift biosensing tests to provide detection results with high spatiotemporal resolution for risk assessment and determine the potential airborne pathogen transmission route.^20,150^ By elevating the sampling flow to 150 L min^−1^, the positive detection rate of airborne SARS-CoV-2 in the inpatient ward environment was significantly improved (72%), while the lower sampling flow rate (50 L min^−1^) resulted in more negative detection results in similar hospital air settings. As a robust filtration matrix, PTFE filter is resistant to longer sampling periods and higher flow rate compared to fragile filters.^153^ However, the use of filtration methods for bioaerosols collection could lead to dehydration, which deactivates the collected pathogens and potentially damages the functional biomolecular structure. Boosting the flow rate could also seriously exacerbate this disruption and significantly affect pathogen viability and integrity. Therefore, the losses need to be considered when analyzing the bioaerosol content on the filter. For instance, pathogen integrity-based assays, which rely on the structure or functionality of an intact virus or bacteria, are not suitable for analyzing filter-collected samples. In contrast, integrity does not have to be a predominant focus for biomolecular-based approaches, such as viral RNA or bacteria DNA quantification, as these analytes can still be detected even after the loss of their viability.

The elution of airborne virus from the filter is another key factor that may impact the accuracy of subsequent quantitative detection. Significant losses in bioaerosol elution and extraction from filter substrates may result in a false negative detection result.^34^ The water-dissolvable filter material can mitigate this inconvenience and achieve rapid liquid sample preparation for subsequent biochemical analysis. As a prominent candidate, dissolvable gelatin filter can significantly reduce bioaerosol damage and losses by exempting the sample elution and extraction processes. The physical collection efficiency of a gelatin filter for submicron bioaerosols is given in Fig. 7g. Similarly, a water-soluble and sponge-like chitosan hydrochloride (CSH) membrane with a heterogeneous micro-nano porous structure (0.12 to 3 μm) was developed for sampling airborne SARS-CoV-2 recently. This CSH membrane is more cost-effective and could dissolve readily in water quickly (*ca.* within 2 min) after bioaerosol sampling.^40^

A miniaturized filtration sampler equipped with a water-dissolvable filter could be a straightforward strategy to collect airborne pathogens on-site. Currently, the filtration-based sampling approaches were commonly incorporated with a nucleic acid detection technique, and its preservation performance for other functional biomolecules (antigenic epitopes) still needs further validation. In addition, the durability and robustness of water-soluble filters for airborne pathogen collection is a concern as environmental factors can directly impact the filtration efficiency.^154^ For instance, high RH can damage the water-dissolvable filters, while high temperatures can accelerate the melting of the filter structure. One study demonstrated that a stable high collection efficiency of gelatin filter was only sustained for 9 min at 2 L min^−1^ flow rate before filter degradation.^155^

#### 4.1.2 Impactor-based sampling approaches

Based on the inertia effect, pathogen-laden aerosols can also be collected by an impactor. When the carrier airflow is sharply curved, the aerosols cannot follow the air streamline but travel continuously in the original direction (Fig. 7b). The impaction surface, which may contain a trapping medium, could bind or capture these inertial aerosols and achieve surface enrichment in the sampling medium.^156^ The impaction efficiency for aerosols heavily depends on the physical properties of airborne particles (*e.g.*, diameter and density) as well as the velocity of driving airflow. One of the main advantages of using impactor-based sampler is its capability to separately collect bioaerosols with different size fractions.^38^ By tuning the inlet nozzle diameter, a series of sampling stages in a cascade impactor could be connected in sequence, and each plate has a gradually decreased nozzle dimension. Airflow passing through a smaller nozzle has a higher velocity, therefore allowing smaller aerosol particles to be collected. Recently, the cascade impactor was utilized to investigate the SARS-CoV-2 virus concentrations and distributions in different size-groups, *e.g.*, large (>10 μm), coarse (2.5–10 μm), and fine (<2.5 μm) aerosols.^11,38,157^ One comparative study also demonstrated a low SARS-CoV-2 infectivity loss over half-hour sampling using a cascade impactor, which indicated the potential for effectively collecting intact and viable pathogens.^38^ Due to the nature of inertial impaction, the sampling efficiency is typically high for large and dense bioaerosols but insufficient for nanoscale bioaerosols. Therefore, impactor-based sampling approaches are mainly used for collecting aerosols larger than 0.5 μm.

As mentioned in Section 2, nanoscale bioaerosols have a long suspension lifetime. Due to its stronger Brownian motion and potential to penetrate into the lower respiratory tract, smaller aerosols are a non-negligible part of on-site airborne pathogen detection and risk assessment. Although the collection efficiency toward small-sized bioaerosols can be theoretically improved by reducing the nozzle inlet diameter, this approach also brings associated problems.^158^ For instance, extremely small nozzle and high flow velocity could induce sonic airflow and shear stress, thereby destroying the pathogens. When utilizing a cascade impactor, there are also considerable target losses in the bioaerosol elution and transfer at each sample stage.

#### 4.1.3 Cyclone-based sampling approaches

Centrifugal force is utilized in cyclone-based sample techniques to deflect inertial aerosols from the airflow and achieve effective airborne pathogens collection (Fig. 7c). Ambient air is typically pumped into a conical-shaped sampler through a tangential inlet. Due to the centrifugal effect and particle inertia, the aerosols rotating through a passage could deposit on the inner surface of the conical-shape sampler. The sampling efficiency can be optimized by changing the physical characteristics such as the cyclone height, body diameter, inlet/outlet size, and flow velocity.^159–162^ For instance, an increased cyclone body height could elevate the aerosols residence time and improve the collection efficiency.^159^ Since this method also relies on the inertia of target aerosols, cyclone is also more efficient to collect larger particles with higher densities.^160^ Though raising the flow velocity can increase the overall performance, the collection efficiency of submicron bioaerosols is still poor.^162^ Cyclone-based samplers have been widely employed in the collection of airborne pathogens due to their portability and simplicity of liquid elution and transfer. A wearable multi-stage cyclone bioaerosol sampler has been used for the collection of airborne viruses in the patient wards.^34^ Bioaerosols with diameter of 1–4 μm and >4 μm can also be simultaneously collected using a commercially available cyclone sampler (*i.e.*, TE-BC251, Tisch Environmental Inc.), and the pathogen sequences have been successfully collected and detected in both size-groups.^163^ A high volume (400 L min^−1^) cyclone sampler was also integrated with a medical robot for the smart sampling of airborne pathogens.^164^ This cyclone-based sampler demonstrated a physical collection efficiency of >90% for bioaerosols larger than 1 μm, and the cutoff size of sampling *d*_*5*0_ was determined to be 0.58 μm.

Despite the advantages of miniaturization and ease-of-integration, the low collection efficiency for submicron bioaerosols may lead to a significant underestimation of the quantification of airborne pathogens, especially the viral particles and submicron-scale bacteria. A previous study has indicated that more than half of the viruses cannot be efficiently collected by cyclone-based samplers.^163^ Therefore, in addition to the impact of external environmental factors, negative test results may potentially be caused by the low sampling efficiency of airborne pathogens.^165^

#### 4.1.4 Electrostatic-based sampling approaches

Electrostatic sampling techniques, as shown in Fig. 7d, rely on the mutual attraction between highly charged airborne particles and a collecting electrode of opposite polarity.^166,167^ The incoming aerosols are commonly charged by a corona discharge in case they carry insufficient electric charge for efficient sampling. The advantages of the electrostatic sampling approaches for airborne pathogen collection include easy-to-miniaturize for portable applications (<1.0 kg and battery powered), high collection efficiencies (>90% for aerosols larger than 1 μm), wide volumetric flowrates (1 to 1000 L min^−1^), wide sampling size range (0.5–200 μm), and low pressure drops during operation.^166,168–170^ Ultra-high collection efficiencies (99.3–99.8%) can be achieved when using a low flow rate (1.2 L min^−1^) at −10 kV applied voltage (Fig. 7h), while it could dramatically reduce to <50% for submicron bioaerosols when elevating the flow rate to 12.5 L min^−1^.^171^ As a ‘gentle’ sampling approach, the maximum bioaerosol traveling velocity is several orders of magnitude lower than that in the impaction- or filtration-based approaches.^171^ Additionally, electrostatic-based samplers allow for point-of-care implementation to capture aerosol from litres of air directly onto a lab-on-chip microfluidic device for subsequent analysis.^167^ Recently, electrostatic-based samplers have also been deployed to collect airborne SARS-CoV-2 in different indoor environments such as food court, train station, and office room. Downstream molecule biology tests reported airborne virus concentrations ranging from 60 copies per m^3^ to 7.8 × 10^2^ copies per m^3^.^172^

**Fig. 8:**
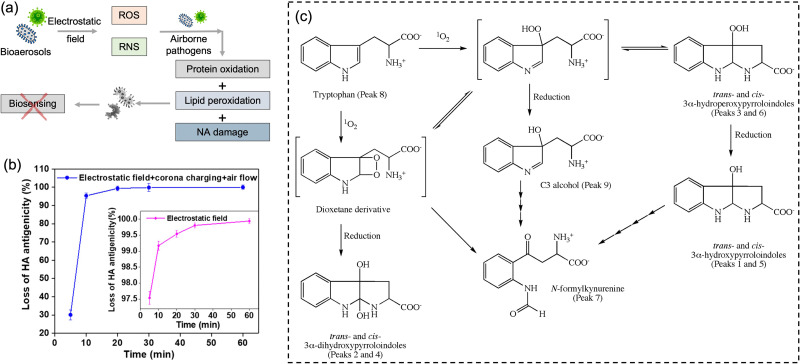
(a) Potential chemical damages to the electrostatic collected airborne pathogens. The reactive oxygen species (ROS) and reactive nitrogen species (RNS) generated by the electrostatic filed may cause protein oxidation, lipid peroxidation, and nucleic acids (NA) oxidation, thereby impacting the subsequent quantitative chemical analysis. (b) The antigenicity loss of pathogen membrane proteins when employing electrostatic fields to collect bioaerosols. More than 90% membrane proteins lost the antigenicity after 10 min of electrostatic air sampling, while >99% with application time of 60 min. The generation of ^1^O_2_ could be the major cause of this high antigenicity loss. Reprinted with permission from ref. 17. Copyright (2020) American Chemical Society. (c) Potential oxidation mechanism of an alpha-amino acid, Tryptophan with ^1^O_2_. Reprinted with permission from ref. 175. Copyright (2009) Elsevier B.V.

It is worth noting that airborne pathogens may suffer a considerable loss in viability after the electrostatic-based collection, as illustrated in Fig. 8a, dominantly due to the biomolecular charging during the sampling process involving high voltage.^173^ The recent studies indicated that the antigenicity losses of pathogen surface protein can be caused by reactive oxygen species (ROS) and reactive nitrogen species (RNS) upon exposure to a strong electrostatic field, such as H_2_O_2_*via* lipid oxidation-derived radicals and ^1^O_2_*via* direct protein peroxidation.^17,174^ It was found that more than 90% membrane proteins of airborne virus lose antigenicity after 10 min of electrostatic sampling when using an applied voltage of −5 kV and a 1.2 L min^−1^ airflow rate (Fig. 8b).^17^ The oxidation mechanism of an alpha-amino acid, Tryptophan with ^1^O_2_, was illustrated in Fig. 8c.^175^ Additionally, the low sampling efficiency for the submicron scale aerosols should also be considered, especially for high volumetric flow rate. For instance, a filter-based sampling device proved more successful in sampling detectable pathogen-laden aerosols than an electrostatic-based sampling system for airborne human coronavirus (CoHKU1) from patient exhalation.^176^

### 4.2 Liquid phase sampling approaches

Biomolecule recognition, such as protein-epitope interaction and nucleic acid hybridization, are crucial for airborne pathogen detection. However, these recognitions can be affected by changes in the environment, such as temperature, pH, and humidity. Dry conditions, in particular, can exert a significant impact on biomolecules, leading to alterations to their structural conformation and functional properties. Therefore, liquid-phase sampling approaches, which directly achieve aerosol-to-hydrosol collection, are often favored for the on-site detection of airborne pathogens.

#### 4.2.1 Virus-into-liquid modification of solid-phase samplers

**Fig. 9:**
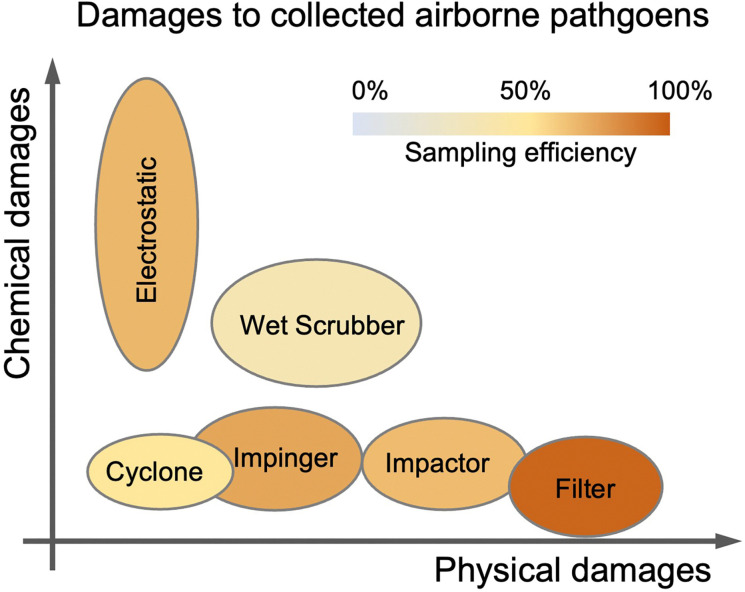
Typical bioaerosols sampling techniques used in transmission risk assessments are arranged according to their physical/chemical damages and sampling efficiencies to airborne pathogens.

There are two obvious problems with solid-phase sampling methods for on-site airborne pathogen detection (Fig. 9). Firstly, bioaerosols elution and liquid sample transfer after collection may lead to the loss of the target pathogens, thereby affecting the accuracy of downstream analysis. The second one is about the biological damage and inactivation during the dry deposition process, particularly for collecting viable contagious pathogens. Desiccation and dehydration may reduce the pathogen viability and the biological integrity, which could hinder downstream detection and risk assessment.

To address these shortcomings, a convenient approach is to carry out aerosol-to-hydrosol modification of the conventional solid phase sampling techniques including impactor, cyclone, and electrostatics samplers. The liquid collection medium, instead of a solid deposition interface, helps maintain the viability and integrity of the virus. Direct collection and enrichment from aerosols to hydrosols for on-site airborne pathogen detection might enhance the preservation and make it easier for the following downstream bioanalytical system to accomplish near-real-time detection. This direct aerosol-to-hydrosol sampling method not only eliminates the time consumption for sample preparation such as bioaerosol elution and transfer but also avoids target losses within this pre-treatment process.

The aerosol-to-hydrosol sampling approaches such as the virtual impactor with a liquid transfer stage, wetted-wall cyclone concentrator, and liquid-phase electrostatic sampler have been adapted to collect airborne pathogens in recent years.^177^ For instance, a liquid transfer stage has been utilized to replace the impaction receiver in the aerosol-to-hydrosol impactor, allowing bioaerosols to be directly collected from the airflow into the liquid flow.^178^ This virtual impactor was used to sample airborne pathogens in the hospital environments, and the bioaerosols can be directly collected into the sterile PBS buffer solution for downstream analysis.^179^

Wetted-wall cyclone concentrators utilize water spray to wet the internal surface of the conical-shaped cyclone body and directly collect aerosols into a small amount of aspirated water buffer.^164^ In one pioneering study, a wetted-wall cyclone was deployed to collect SARS-CoV-2 aerosols in hospital wards and successfully facilitated the downstream tests to estimate the aerodynamic distribution and transmission distance (∼4 m) of virus-laden aerosols.^180^

Additionally, the aerosol-to-hydrosol concept can also be adapted to electrostatic-based sampling approaches by replacing the solid-state deposition surface with a continuously flowing liquid. For instance, a high-flowrate electrostatic sampler (HAFES) was proposed to collect various types of airborne pathogens including human coronavirus 229E, influenza A virus subtypes H1N1 and H3N2, and bacteriophage MS2.^168^ Apart from the discharge electrode, a thin layer of pathogen lysis buffer was utilized as a collection electrode within the system. The collection efficiency can be optimized to reach about 88% for human HCoV-229E coronaviruses. In another liquid electrostatic sampling system, approximately 80% of SAR-CoV-2 virus-laden aerosols ranging from 100 nm to 5.0 μm can be collected with an airflow rate of 40 L min^−1^.^172^ To further improve the stability and integrity of the collected airborne pathogens, the chemical compositions of the sampling liquid could be further optimized. For instance, protective agents like ascorbic acid (AA), sodium azide, and uric acid can be added to the PBS sampling liquid reservoir to mitigate the inevitably damage caused by the generated ROS and RNS from electrostatic precipitation (Fig. 8).^181^ These protective agents can reduce the biomolecular damage by preventing the formation of toxic compounds such as H_2_O_2_, ^1^O_2_, and ONOOH produced by corona discharging process.^173^

The conventional approach for aerosol-to-hydrosol sampling involves the utilization of water or other liquids with low viscosity as the medium for collection. Hence, the evaporation issue at the “dry-to-wet” interface can also have an impact on the performance of sampling. This issue not only restricts the overall sampling duration to shorter time periods but also impacts the efficiency of sampling and the viability of pathogens. The non-evaporating and higher viscosity liquids, such as glycerol and heavy white mineral oil, have been proposed to sample airborne bacteria and fungi for several hours.^182^ For downstream biosensing, a recent study has demonstrated that additive glycerol promoted the detection of African swine fever virus (ASFV) and SARS-CoV-2 with one-pot recombinase polymerase amplification (RPA)-CRISPR/Cas12a method and a smartphone-equipped device.^183^ During the initial phase of the RPA reaction, the viscosity of glycerol led to the phase separation of the RPA and CRISPR/Cas12a system, which increased the detection efficiency. Nevertheless, the detection effectiveness in different liquid environments containing glycerol may require further validation for specific biosensing technologies, including the selective recognition between different biomolecules.

#### 4.2.2 Liquid impinger-based sampler

As one of the representative aerosol-to-hydrosol collection approaches (Fig. 7e), liquid impinger-based samplers, such as the all-glass impingers (AGIs), midget impingers, and SKC BioSampler, have been widely used for collecting airborne pathogens including bacteria and viruses.^184,185^ In an liquid impingement system, the airborne particles are transported and accelerated to high velocity through a circular or rectangular nozzle and then expected to impact into a collection liquid through inertia. By leveraging the aerosol-to-hydrosol approach, liquid impinger-based samplers could improve the viability and retention of the airborne pathogens. Meanwhile, the high-speed airstream creates a high degree of turbulence and stimulates the formation of bubbles inside the sampling liquid, which could increase the collection efficiency for tiny bioaerosols (Fig. 9). However, the formation of air bubbles may also exacerbate re-aerosolization, thereby losing the retained bioaerosols.

As a representative of the swirling aerosol collector, the commercial SKC BioSampler provides an improved performance on reducing the reaerosolization.^186^ It works by projecting airborne particles into the sampling liquid in a swirling motion through three micrometer-scale tangential nozzles. This sampling process combines impaction and centrifugation and therefore provides an improved sampling efficiency and minimizes re-aerosolization compared to conventional liquid impaction approaches.^186,187^ The sonic airstream velocity enables this aerosol-to-hydrosol method to have improved collection efficiency for submicron bioaerosols with a cutoff size of about 300 nm.^186^ A recent research has directly compared the BioSampler performance to those of the PTFE and gelatin filter sampler, midget sampler, cyclone sampler, and Sioutas impactor.^38^ Particularly noteworthy was that the swirling aerosol samplers demonstrated the U-shape sampling effectiveness curve in collecting nanoscale aerosols, as shown in Fig. 7i.^155^ The lowest sampling efficiency was found to be located at the diameter range of 30–100 nm (penetrating window), while a high efficiency of >90% was observed at 10 nm due to the enhanced Brownian diffusion inside the swirling sampler.^155,188,189^ Additionally, the inlet design of all-glass impinger (AGI) and BioSampler, which mimic the human upper respiratory tract, could lead to a low inlet efficiency of about 80% for aerosols larger than 5 μm.^190^ Therefore, these effects, including the size-dependent inlet efficiency and sampling efficiency, must be taken into account when conducting the on-site sampling-to-biosensing–based infection risk assessment. A more precise estimate of the airborne pathogen concentration can be obtained by considering the size-fractionated airborne pathogen distribution.

#### 4.2.3 Miniaturized wet scrubber-based methods

An alternative aerosol-to-hydrosol approach to collect airborne pathogens is based on the liquid droplet sprays and wet scrubbers.^191^ The wet-scrubber-based sampling approach collects aerosols *via* direct scavenging with liquid droplets. This approach combines multiple sampling mechanisms including the turbulent diffusion, inertial impaction, interception, and gravitation to achieve the effective collection of airborne particles.^192,193^ During the sampling, bioaerosols collide and merge with liquid droplets through coalescence. In conventional industrial wet-scrubbers, liquid droplets generated by pressurized nozzles cannot efficiently collect airborne particles with diameter less than several micrometers. Additionally, the collection liquid volume is generally large, and the airborne virus particle cannot be beneficially concentrated for downstream analysis. To tackle these issues, many novel strategies have been applied to modify the wet scrubbing-based collector, including twin fluid atomization, droplet charging, high-pressure spray, and ultrasonic microdroplet generation.^194–196^ Recently, a portable and battery powered wet-scrubber device was developed for collecting airborne pathogens.^191^ This device generated a mist of 4 μm liquid droplets in a non-linear flowing path. By combining the impaction, diffusion, interception, centrifugation, and liquid scavenging effect, this miniaturized aerosol-to-hydrosol scrubber device demonstrated an improved sampling efficiency and viable pathogens preservation for rapid downstream analysis.

Despite an improved bioaerosol sampling performance, the wet scrubber still demonstrated a lower collection efficiency for nanoscale bioaerosols compared with other high-efficiency sampling approaches. To prevent false-negative results and underestimation of the transmission risk of airborne pathogens, practical on-site pathogen-laden aerosol quantification still needs a lengthy sampling period and improved efficiency for small bioaerosols.

### 4.3 Microfluidic-based miniaturized sampling system

**Fig. 10:**
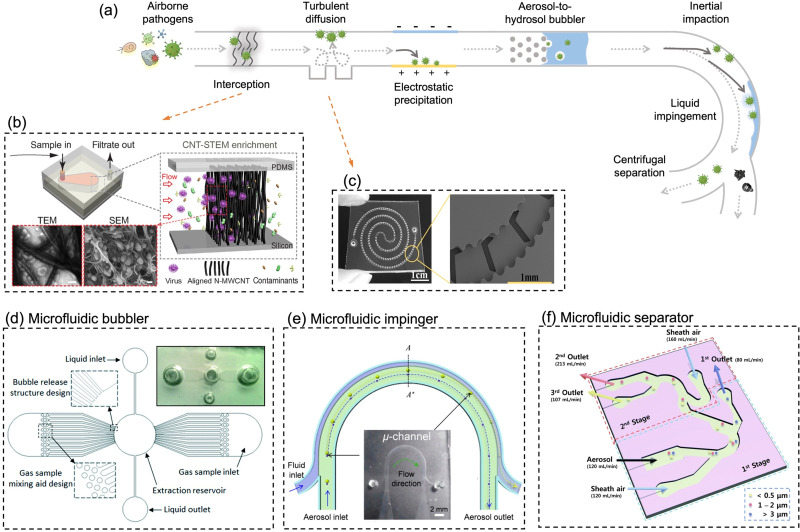
(a) Schematic illustration of miniaturized physical structural design for pathogen-laden aerosol collection. The main collection principles include interception, turbulent diffusion, inertial impaction, centrifugation, liquid impingement, and electrostatic precipitation. (b) *In situ* growing carbon nanotube array in a microfluidic system for efficient bacteria and virus collection and enrichment. Reprinted with permission from ref. 218. Copyright (2016) American Association for the Advancement of Science. (c) Herringbone structure design in a double spiral microfluidic channel to enhance flow turbulence and bioaerosols collection efficiency. Reprinted with permission from ref. 197. Copyright (2016) American Chemical Society. (d) A microfluidic-based and on-chip aerosol-to-hydrosol bubbler for airborne pathogens collection. Reprinted with permission from ref. 199. Copyright (2016) the Royal Society of Chemistry. (e) Microfluidic-based swirling impinger for continuous bioaerosol sampling. Reprinted with permission from ref. 200. Copyright (2017) American Chemical Society. (f) Continuous microfluidic separator using inertial effect to concentrate airborne virus and bacteria. Reprinted with permission from ref. 205. Copyright (2015) the Royal Society of Chemistry.

With the rapid developments and advancements in microfabrication technologies, microfluidic-based bioaerosol samplers have offered a promising avenue for swift airborne pathogens collection and rapid subsequent detection.^17,177,197–202^ Compared to the conventional sampling approaches, microfluidic chips with miniaturized physical structural design have shown the advantages of low cost, fast response, automation, and compatibility for fast on-site bioanalysis.^203^ As shown in Fig. 10a, interception, turbulent diffusion, inertial impaction, centrifugation, liquid impingement, and electrostatic precipitations are still the leading principles used by the microfluidic platforms for pathogen-laden aerosol sampling.^197–200,202,204^

For instance, based on the principle of Greenburg–Smith impingement sampler, a miniaturized micro-bubbler has been developed for gas-particle mixing, bubble release, and aerosol-to-hydrosol sampling (Fig. 10d).^199^ In order to ensure stable micro-bubble generation and high collection efficiency, the inner surface of microchannel and micro-pillar array was coated with hydrophobic Teflon layer. The proposed micro-impinger was able to collect and concentrate the aerosol particles into a much smaller solution volume (250 μL) with a gentle airflow rate (10–20 mL min^−1^) when compared to the conventional impingement collector. For bioaerosols larger than 0.5 μm, the micro-impinger achieved a satisfactory collection efficiency of >95%. However, further investigation is still needed to fully understand the sampling performance toward nanoscale bioaerosols, such as a single airborne virus particle, as shown in Fig. 1.

In addition, airborne particles can also be collected in microfluidics by leveraging the technique of centrifugation.^200,204^ Instead of direct solid-phase impaction on the channel, a two-phase microfluidic system, which combined the sampling airflow and sheath fluid, was developed to directly transfer the airborne pathogens into liquid in a curved microchannel (Fig. 10e).^200^ This micro-sampler was characterized using standard polystyrene latex particles and bacterial aerosols ranging in size from 0.6 to 2.1 μm. Under the optimal volumetric airflow rate of 0.6 L min^−1^, more than 80% of 0.65 μm bioaerosols and 98% of 1 μm bioaerosols could be efficiently collected into the constant sheath fluid flow (0.3 mL min^−1^). Moreover, the inertial and centrifugal forces can be alternatively used for continuous aerosol size separation, as shown in Fig. 10f. An inertial microfluidic aerosol sizer was also proposed recently to separate target bioaerosols into three size groups (*e.g.*, <0.5 μm, 1–2 μm, and >3 μm).^205^ Despite this approach, it does not achieve a high enrichment efficiency for bioaerosols by itself, it enables downstream sampling and bioanalytical devices to acquire the size distribution of virus-laden aerosols, much like the traditional multi-stage impactors. Apart from inertia-based sampling, external deflection forces such as electrostatic trapping forces have be utilized to precipitate or manipulate airborne particles in a microfluidic system.^170,202^

A unique feature of the microfluidic-based bioaerosol samplers is that the trajectory of the aerosol can be regulated by changing the internal geometry and shape of microfluidic channel so that the airborne pathogens can be manipulated and collected in a predetermined area or medium.^206^ For instance, the herringbone grooves located on the inner channel surface can induce the micro-vortex and enhance the turbulent deposition of bioaerosols at the inner surface of the microfluidic channel (Fig. 10c).^197^ One recent study proposed a 600 μm-wide flow channel with 130 μm-deep herringbone grooves, which achieved 100% efficiency at airflow rates of 2–4 mL min^−1^ and 98% at 6 mL min^−1^ for collecting bacterial aerosols.

Compared with conventional pathogen-laden aerosol sampling approaches, microfluidic-based samplers provide a number of exceptional advantages. Miniaturized microfluidic samplers obviously outperformed conventional samplers in terms of integration level, low power consumption, and post-processing simplicity for on-site airborne pathogens quantification and real-time risk assessment. However, there are also obvious issues and challenges that require immediate attention. For instance, the blockage of microfluidic channels can be a potential issue that affects the performance and reliability of airborne pathogen and bioaerosols sampling. Additionally, there is still room for improvement in the effectiveness of collecting nanoscale bioaerosols, particularly for the majority of airborne viruses with diameters as small as 100 nm. Although the microfluidic-based bioaerosol sample may enrich the bioaerosols into a smaller volume of solution, the airflow rate is also noticeably decreased compared to traditional approaches. Therefore, it can be beneficial to use the effective sampling enrichment capacity (ESEC) to assess the overall effectiveness of the sampling system, which is given by
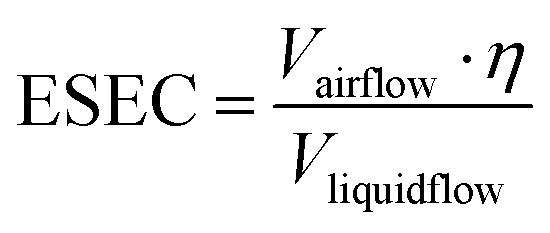
where *η* is the sampling efficiency, *V*_airflow_ is the volumetric airflow rate (in L min^−1^) during sampling, and *V*_liquidflow_ is the volumetric liquid flow rate (in L min^−1^) of the collection medium, or instead refers to the volume of static collection medium per unit time (*V*/*t*, where *t* is the sampling duration). The ESEC values for some of the microfluidic-based and conventional aerosol-to-hydrosol sampling approaches are given in Table 2.

**Table 2:** Effective sampling enrichment coefficient (ESEC) of conventional and microfluidic based aerosol-to-hydrosol sampling systems. Glossary: C.A., commercially available devices. *μ*, microfluidic based sampling systems

Type	Sampling system	Airflow rate	Liquid flowrate or volume	ESEC	Ref.
C.A.	BioSampler (SKC Inc.)	12.5 L min^−1^	20 mL or 5 mL	125 (20 mL) or 2500 (5 mL)/min	a
C.A.	Wet cyclone (NIOSH-BC251)	2–10 L min^−1^	15 mL and 1.5 mL in two stages respectively	133–6667/min	a
*μ*	Turbulent diffusion system	2–4 mL min^−1^ with 100% efficiency	Eluted with 100 μL TSB medium	20–60/min	198
		6 mL min^−1^ with 98.2%			
*μ*	Interception	71 mL min^−1^, (∼100% efficiency)	5 μL lysis buffer	4733–6000/min	199
*μ*	Impingement	10–20 mL min^−1^ (94–100%)	(into ∼250 μL)	40–80/min	200
*μ*	Centrifugation	0.6 L min^−1^ (>80% for 650 nm; >98% for >1 μm)	0.3 mL min^−1^	2000	201
*μ*	Electrostatic system 1	4–10 L min^−1^	into 50 μL min^−1^	80 000	202
*μ*	Electrostatic system 2	> 10 L min^−1^, 40% (−80%) efficiency with −1.8 kV charging and −7 kV collecting voltage	0.48 mL min^−1^	20 833 (×40%)	203
*μ*	Electrostatic system 3	1.2 L min^−1^	0.5 mL	2400/min	25
*μ*	Wet cyclone	960 L h^−1^, (with 99.5% efficiency for 300 nm aerosols)	0.4 mL h^−1^	2.4 × 10^6^	181

a Indicated data from the manufacturer for a commercially available aerosol-to-hydrosol sampling device.

The concentrations of fully diluted airborne pathogens are typically at an extremely low level compared to patient specimens. For microfluidic-based sampling devices, which can handle only a limited volume of air, it is difficult to collect sufficient number of pathogens for downstream analysis even if a high ESEC value is achieved. For instance, a microfluidic-based sampling system with a limited air flowrate of 2 mL min^−1^ may not be practical for real-time and rapid pathogen quantification. Hence, it is crucial to conduct further validation studies to assess the applicability of a microfluidic-based sampling system regarding various detection scenarios and types of airborne pathogens.

### 4.4 Practical strategies to improve the sampling efficiency

Based on the physical properties of the pathogen-laden aerosol (see Section 2.3), a number of airborne pathogens, especially virus and small size bacteria, can be carried by submicron aerosols. Despite having a low mass content, nanoscale bioaerosols may be more prevalent and cause a non-negligible risk of infection because of their capacity to traverse large distances and deposit themselves directly on the low respiratory tract. Consequently, it is essential to increase the sampling effectiveness of collecting submicron-scale bioaerosols. This is particularly true with microfluidic-based samplers, which have a poor collection efficiency for bioaerosols smaller than 0.5 μm. Condensation growth-based approach, also known as the hygroscopic growth, and mixing-type bioaerosols amplification unit (mBAU), is one of the most common and effective strategies to improve the physical collection efficiency of submicron-scale aerosols.^39,207–209^ Condensation growth approach, as shown in Fig. 7f and j, is able to enlarge the aerosols to larger sizes through hygroscopy so as to improve the sampling efficiency. Generally, when solid bioaerosols pass through a supersaturated steam chamber or laminar airflow tube, the water vaper condenses into a liquid state and adheres to the aerosols. Aerosol size enlargement can improve its inertia, allowing for efficient collection by a following bioaerosol sampler. At present, condensation growth approaches have been integrated with many aerosols-to-hydrosol sampling methods, such as liquid impingement and impaction (Fig. 7j–l).^209,210^ For instance, the condensation growth approach can directly improve the physical collection efficiency of swirling bioaerosol sampler from 10% to 99% for 100 nm bioaerosols.^209^ The viability preservation of airborne MS2 bacteriophage was also improved by 22 times compared to the bare commercialized BioSampler. The condensation-based bioaerosols sampler have also been deployed for collecting airborne virus in different patient environments.^211^ Taking advantage of the gentle aerosol-to-hydrosol sampling process and condensation-induced high sampling efficiency, viable viruses in concentrations ranging from 6 to 74 TCID_50_ units per L have been successfully identified in patient wards.^211^

In addition, the condensation growth device has also been miniaturized into a portable microfluidic system by harnessing 3D printing, photolithography, and passive cooling techniques.^212,213^ Compared to the conventional bench-top devices, hygroscopic systems were not only far more compact, affordable, and power-efficient but also demonstrated remarkable performance in facilitating the optical detection of bioaerosols down to 10 nm. By integrating with microfluidic-based samplers, as reviewed in Section 4.3, these micro-condensation aerosol sampling systems may be able to provide excellent sampling efficiencies over a broad aerodynamic size range from 10 nm to 100 μm, thereby offering solid support for on-site airborne pathogens detection and accurate risk assessment.

### 4.5 Fast bioaerosol pre-treatment strategies

The obtained aerosol samples may include a wide range of complicated chemical and biological components dispersed in the air. During the bioaerosols treatment, these compounds may impact the integrity of the collected airborne pathogens and make it challenging to identify them later using bioanalytical methods. For instance, after being enriched in the sampler, metal ions and ROS in the air may have an impact on the biomolecular structure and hasten the breakdown of whole pathogens or its particular recognition sites (such as proteins and nucleic acids). This might lead to inaccurately low airborne pathogen concentration readings or false negative findings.^214,215^ Additionally, in some public areas, the concentrations of a particular type of pathogen floating in the air may be quite low. However, prolonged exposure in such environments may still result in a cumulative intake of pathogens or bioaerosols at a minimum infectious dose. Since the majority of biosensing systems for pathogens or bioaerosols detection possess a ‘non-zero’ detection threshold (*i.e.*, the limit of detection, LoD), negative results might not completely rule out the potential of infection and health impact. Target selective-enrichment and preconcentration prior to quantitative bioanalysis may greatly benefit on-site biosensing detections and boost the accuracy of on-site risk assessment. In this section, pre-treatment strategies including impurity removal, selective target enrichment, and effective to-be-tested sample delivery for downstream biosensing are reviewed and discussed.

Based on the physicochemical characteristics of airborne pathogens, various technologies that leverage physical separation and affinity-based extraction methods can be applied to purify the target bioaerosols.^198,216,217^ Considering the necessity of fast processing for on-site bioaerosols detection, we place an emphasis on rapid enrichment systems that can be miniaturized rather than large and resource-intensive bench-top equipment. Recently, a 3D porous microfluidic system with a robust array of aligned carbon nanotubes (CNTs) was proposed for virus purification in field samples (Fig. 10b).^218^ This CNT array growing on the side wall of a microfluidic device can be engineered with a tunable inter-tubular distance ranging from 17 to 25 nm.^218^ Based on the principle of physical separation, this method could not only enrich viruses by at least 100-fold but also eliminate environmental contaminants to facilitate direct virus detection in a subsequent step. Using a stamping technique to pattern Fe catalytic particles (CNT grow-seeds), the nitrogen-doped CNT array can be further patterned into a herringbone structure to enhance the mixing of the sample inside the microfluidic channel through the chaotic flow.^219^ In addition, the stamping method was able to pattern Fe catalytic particles with a density gradient, resulting in the formation of multiple herringbone CNT zones with different physical inter-tubular distances ranging from 22 to 720 nm. The integration of CNT array zones with varying inter-tubular distances can be used not only to enrich a broader spectrum of airborne particles but also to purify bioaerosols of different sizes on the same device and facilitate size-resolved pathogen biosensing.

In contrast to physical separation, affinity-based enrichment techniques (*e.g.*, solid-phase extraction and hydrogel protein purification) target specific pathogen recognition sites, such as nucleic acids or proteins.^220–222^ Recently, an affinity-based microfluidic centrifugal device was developed for enriching viral RNA into a detection-ready sample.^220^ This centrifugal microfluidic device leveraged the magnetic virus-affinity particles to isolate intact pathogen sequences through multiple lab-on-chip processes including magnetic manipulation, affinity binding, flushing, and RNA elution. This integrated microdevice can rapidly remove complex matrices from the field aerosol-to-hydrosol samples within 15 min. Moreover, chemically modified polymers, such as hydrogels with affinity baits, can be used to purify and enrich pathogens *via* interactions with target proteins.^221,223^ Recently, affinity-based hydrogel nanoparticles (Nanotrap) have been used to swiftly enrich SARS-CoV-2 by binding with viral spike proteins.^224^ The entire preparatory procedure, including virus enrichment, enzymatic RNA extraction, and elution, required approximately 10 min.

For target pathogen purification and extraction, different microfluidic systems depending on solid-phase extraction and structure-based physical separation have received a lot of attention to date. These practical, compatible, affordable, and microfluidic-based virus pretreatment strategies may also have the potential to offer strong technical support for achieving the near-real-time and accurate airborne pathogen monitoring and on-site bioaerosols risk assessment.

## 5 On-site airborne pathogens detection

**Fig. 11:**
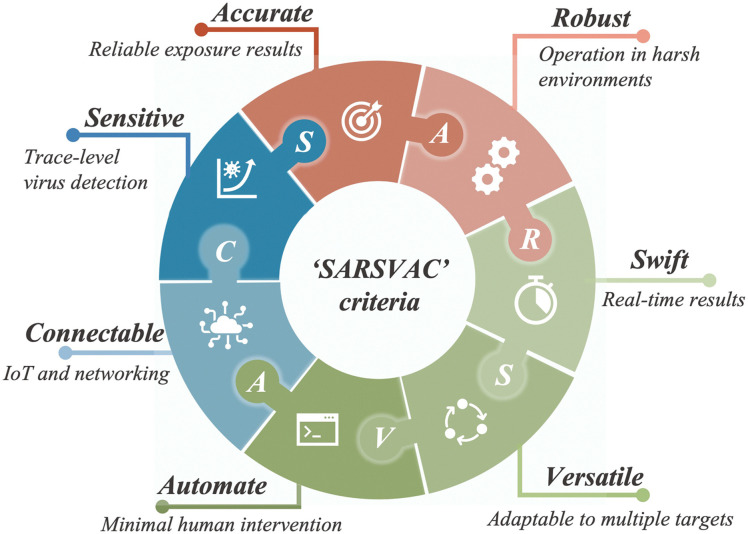
Proposed “*SARSVAC*” criteria for evaluating biosensing system in the field of on-site airborne pathogens detection and real-time transmission risk assessment. In particular, the ideal on-site airborne pathogen sensing system should match the proposed SARSRVAC criteria and be Sensitive, Accurate, Robust, and Swift for Versatile, Automated, and Connectable sensing applications.

In a manner different from existing clinical diagnosis approaches, on-site bioaerosol sensors need to be more sensitive, accurate, robust, and swift for versatile, automated, and connectable sensing applications. To further clarify the research priorities for the on-site airborne pathogen biosensor development, we propose a set of “*SARSVAC*” criteria, as shown in Fig. 11. Under this scheme, we evaluate and summarize the novel bioanalytical chemistry technologies developed in the recent years. In detail, high sensitivity and low LoD should be the first priority for developing on-site pathogen-laden aerosol sensors. Due to the possible low pathogen concentrations in the air, superior sensitivity is critical to eliminate false negative results, to reduce the overall time consumption of aerosol sampling-to-biosensing turnaround, and to provide more accurate real-time information on airborne transmission. Strategies such as employing nanomaterials, labelling, signal amplification, target replication, and novel biosensing concepts have been utilized to improve the sensitivity.^44,225–227^ Another essential sensor trait is the sensor accuracy and precision, which is decisive for a reliable biosensing readout for predicting on-site bioaerosol exposure and determining a precise transmission risk level. Aerosol samples that have been concentrated in a liquid solution may release numerous unexpected chemicals and particles, which may hinder specific molecular interactions (false negative) or trigger unexpected binding to biosensors (false positive). Various approaches have been proposed recently to improve the biosensing accuracy, including the use of blocking agents, sample pretreatment, and novel intrinsic biosensor design (*e.g.*, self-referencing channel).^18,228–230^ Meanwhile, a novel biosensor design could also enable on-site pathogen sensing system to work in different harsh environments.

Swiftness is another important trait to allow exposure measurement with high spatiotemporal resolution and early-stage warnings. Currently, numerous biosensors can detect trace quantities of pathogens within 10 min.^18,19,231,232^ In addition, biosensors should be versatile and readily adaptable to detect various types of airborne pathogens and variants, given the rapid mutation and emergence of novel pathogens. Last but not least, automated and connectable sensor network could allow for measurement of multilevel (personal–private–public, 3P) exposures, *i.e.*, with a high degree of spatiotemporal resolution. On-site airborne pathogens sensing services can be connected to the central network and provide real-time accessible data to facilitate evidence-based interventions of airborne pathogen transmission.

The proposed “*SARSVAC*” criteria could further clarify the development and research priorities of airborne pathogen biosensing technologies. In the sections that follow, we will review various on-site airborne pathogen detection strategies, highlighting the recent advancements and prospective challenges.

### 5.1 Pathogen recognition strategies

**Fig. 12:**
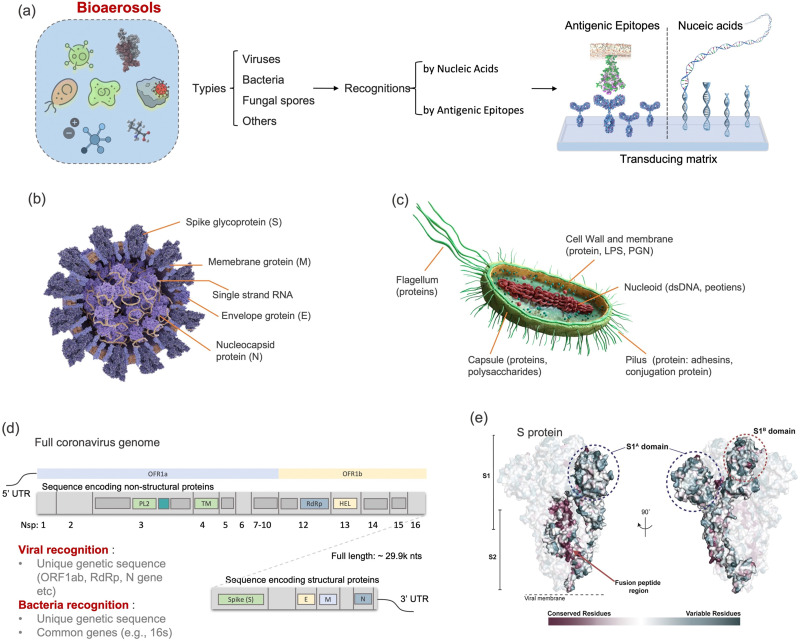
(a) Schematic illustration of biosensing recognition sites for detecting airborne pathogens like viruses, bacteria, and fungal spores. (b) Cross section of coronavirus, *i.e.*, SARS-CoV-2, which demonstrated the typical recognition sites, including a variety of structural and non-structural proteins, a lipid bilayer envelope, and encapsulated RNA sequence. (c) Cross section of a Gram-negative bacteria with different recognition sites. (d) Full SARS-CoV-2 genome and its two sub-genomic regions, which encoded the non-structural proteins (nsp, *e.g.*, nsp13 HEL, nsp12 RdRp) and structural proteins (*e.g.*, S-, N-, E-, and M-proteins). The potential RNA recognition sites widely used for biosensing and nucleic acid detection involve ORF1ab, RdRp, S, N, E, and nsp13. (e) Common epitopes recognition sites in spike protein of coronavirus. The two functional subunits, S1 and S2, which are responsible for mediating attachment to host cells and membrane fusion can be recognized by angiotensin-converting enzyme 2 (ACE2), monoclonal antibodies, and aptamers. Reprinted with permission from ref. 235. Copyright (2020) Elsevier B.V.

Airborne pathogens may consist of bacteria, viruses, fungi, or other microorganisms, as shown in Fig. 12a. As one of the most prevalent airborne pathogens, bacteria are unicellular microorganisms that can cause a variety of diseases, including tuberculosis, *Streptococcus*, and Legionnaire's disease. Viruses are even smaller infectious agents that can cause a range of ailments, from the common cold to more severe diseases such as COVID-19, chickenpox, monkeypox, and measles.^233^ For instance, coronaviruses are enveloped single-strand positive-sense RNA viruses with an average diameter of about 100 nm. As shown in Fig. 12b, human coronaviruses are mainly constructed by a variety of structural and non-structural proteins, a lipid bilayer envelope, and encapsulated nucleic acid materials.^234,235^ Fungal aerosols, which include yeasts and spores, are the third group of airborne pathogens.^50^ Some types of fungi can also cause respiratory infections such as aspergillosis and histoplasmosis. For specific pathogens or general bioaerosols, there are mainly two groups of specific recognition targets for on-site and rapid detection: nucleic acids (DNA or RNA sequences) and antigenic epitopes (antigen proteins).

#### 5.1.1 Recognition by nucleic acids

Pathogens contain either DNA or RNA as their genetic material. DNA is the genetic material found in most microorganisms, including bacteria and viruses like adenovirus, whereas RNA is found in certain viruses such as influenza virus, SARS-CoV, and SARS-CoV-2. From a bioanalytical point of view, these unique sequences, as shown in Fig. 12d, can be exploited as efficient recognition sites for developing highly specific biosensors.

16s rDNA is a conserved gene sequence found in all bacteria and extensively used for the identification and classification of bacteria. The general 16s rDNA sensing techniques involves the amplification of the 16s rDNA gene from the bacterial sequence, followed by the sequencing of the amplified product to identify the bacterial species present in the bioaerosols sample. Because of the universality of this method for bacterial detection, the recognition site of 16s rDNA has been very versatile in both clinical and environmental sensing applications.^236^ Similarly, the gp43, p27, and ITS genes are all genetic biomarkers for fungal identification and classification. ITS sequence located between the 18S and 28S region is highly variable and evolves rapidly, making it useful for identifying and differentiating between fungal species.^237^

Due to the diversity of viral families, it is difficult to find a universal genetic marker for detecting all types of viruses. For DNA viruses such as adenoviruses and smallpox virus, one conserved genetic region is known as the DNA polymerase gene, which encodes for the enzyme responsible for DNA replication. Similarly, the RdRp (RNA-dependent RNA polymerase) gene, which may be utilized as a target for identifying airborne viruses, is present in a variety of RNA viruses, including coronaviruses and influenza viruses. Coronaviruses such as SAR-CoV and SARS-CoV-2 possess a positive-sense RNA sequence with 26 to 32 kilo-bases in length.^238^ In clinical diagnosis, nucleic acid detection, *e.g.*, polymerize chain reaction (PCR), is currently considered as the gold standard thanks to its well-characterized sensitivity and accuracy. For instance, genomic probing sites located in N gene (encoding nucleocapsid protein), Orf1ab gene (encoding open read frame 1ab protein), E gene (encoding envelope protein), RdRp gene (encoding RNA dependent RNA polymerase), and S gene (encoding spike protein) shown in Fig. 12d are commonly used for recognizing SARS-CoV-2.^34,239^ Integrated bioaerosol sampling strategies, such as the inertial impactor embedded in a micromachined silicon chip, have been recently proposed for facilitating point-of-care RT-qPCR detection of the airborne SARS-CoV-2 viruses.^240^ However, several characteristics of PCR tests including being resource-intensive, cumbersome, and time-consuming hampers their applications in real-time airborne virus detection and on-site virus transmission monitoring. Numerous high-performance, quick, and practical nucleic acid detection technologies have been suggested and developed recently.^241,242^ As an prominent gene-editing technology, clustered regularly interspaced short palindromic repeats and associated proteins (CRISPR/Cas)-based assays have been proposed for the sensitive and swift recognition and detection of pathogen genes.^225,243–245^ In the CRISPR/Cas-based biosensors, the cleavage activity of the nuclease-inactive ribonucleoprotein complex, which contains Cas-protein and crRNA, can be selectively activated by binding to the complementary sequence of the pathogen target.^246^ The activated complex can be used to indiscriminately cleave any surrounding sequence and generate a sensible signal. For instance, the CRISPR-Cas13a assay was developed for the direct detection of SARS-CoV-2 RNA with a superb sensitivity by 100 copies per μL and a swift turnaround of 30 min.^225^ Although this sensing approach was initially designed for clinical diagnosis, its swift and sensitive characteristics meet the criteria for on-site pathogen detection. Additionally, this CRISPR-based biosensing assay can be integrated with a mobile phone-based readout device to fulfill the criteria of prospective sensor networking and connectable IoT. To date, the CRISPR/Cas approaches have not been directly applied to detect on-site collected aerosol samples. The sensing performances such as recognition accuracy and stability need to be further verified when the assay reagents are directly exposed to diverse aerosol compounds from the environment.

A major disadvantage of nucleic acid detection approaches for usage in on-site airborne virus detection is the demand for additional sample pretreatment procedures, including pathogen lysis, release of inner nucleic acids, and removal or inactivation of NA-degrading enzymes. An integrated microfluidic system, as explained in Section 4.5, is more appealing for the sampling, pretreatment, and delivery of pathogen aerosol samples for biosensing.

#### 5.1.2 Recognition by antigenic epitopes

The antigenic epitopes of airborne pathogens represent another group of recognition sites for quantitative biochemical analysis. Fig. 12e indicated the epitopes sites for recognizing the S protein of SARS-CoV-2. As part of pathogen proteins, epitopes facilitated the specific molecular binding of antibody-antigen pairs through epitopes-paratopes interactions. For instance, targeting the viral spike and nucleocapsid proteins, many SARS-CoV-2 antigen biosensors have been developed for both clinical diagnosis and environmental surveillance applications.^247–249^ A fast bacteriological immunoassay and an integrated microfluidic device capable of capturing and enriching airborne microorganisms like *Mycobacterium tuberculosis* were also developed. In addition, aptamer or synthetic polymer receptors (*e.g.*, molecular imprinted polymers) can also be utilized to detect antigen.^248–250^

The epitopes recognition strategy is mainly based on the binding affinity (*K*_D_) between the receptor and target proteins. Taking the SARS-CoV-2 spike protein as an example, the binding affinity with human angiotensin-converting enzyme 2 (ACE2) receptor is about 14.7 nM.^251^ In contrast, two optimized oligonucleotide aptamers selected through systematic evolution of ligands by exponential enrichment (SELEX) possess affinities of 5.8 nM and 19.5 nM.^249^

**Fig. 13:**
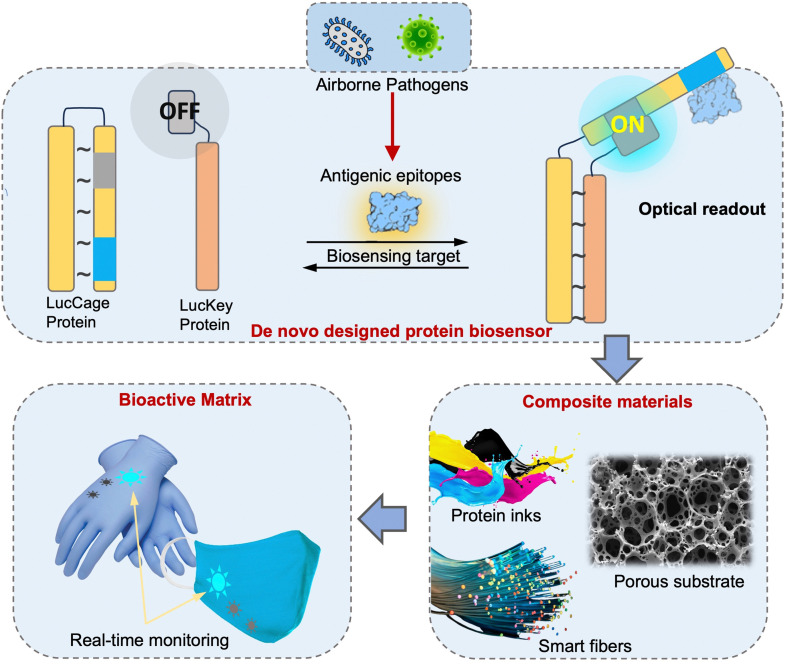
Schematic illustration of the biosensing mechanism of using *de novo* designed functional proteins ‘lucCage’ for universal biomarkers detection. Protein switches (lucKey-lucCage) that enable the detection of antigenic epitopes from various pathogens can be combined with composite substrates such as regenerated silk fibroin (RSF) to fabricate bioactive matrices for on-site and rapid pathogen detection. Schematic ideas were followed as represented in previously published article ref. 253.

Recently, a novel class of epitope-based biosensor was proposed by de-novo designing protein switches and modulating inter-/intra-molecular interactions.^252,253^ As shown in Fig. 13a, the modular caged target-binding-motif is designed to selectively interact with the target proteins or epitopes. The specific binding also drives the conformational states change and leads to switch from a dark state to an “open” luminescent state. This novel epitope-based biosensing approach has also been exploited to detect the SARS-CoV-2 S protein, with a detection limit of 15 pM (Fig. 13b–d).^253^ Combining with a suitable bioaerosol sampling system, the *de novo* designed protein biosensor could be a promising candidate for swift and sensitive airborne pathogen detection.^252–254^ Designing a suitable material host for *de novo* designed protein switches without altering their intrinsic thermodynamics and stability would enable the development of smart sensing formats for monitoring exposure to pathogens and toxins for risk assessment. Notably, a similar technology using a B-cell-based luminescent biosensor has been developed into a commercially available device for monitoring different airborne pathogens.^255^

The most outstanding advantage of epitope-based recognition strategies is their swiftness and flexibility. Generally, most of the recognition approaches by antigenic epitopes take less than 30 min, which may benefit the on-site and real-time airborne pathogen quantification. Ideally, the epitopes receptor of biosensors should be selective and specific to the designated target protein. However, the majority of protein receptors, including pathogen antibodies, are promiscuous and may bind with a variety of non-specific biomolecule.^256^ Egelhofer *et al.* have tested the specificity of about 200 antibodies and found that more than 25% have substantial problems of specificity.^257^ One factor that contributes to promiscuity is the presence of conserved epitopes among different bacterial or virus species. For example, certain surface proteins or lipopolysaccharides are shared among different strains of bacteria, allowing for recognition by a single antibody. Regarding SARS-CoV-2, ten antibodies against the SARS-CoV structural proteins including S, N, M, and E proteins demonstrated non-negligible cross-reactivity to SARS-CoV-2.^258^ In detail, the binding affinities between SARS-CoV-2 S protein and three monoclonal SARS-CoV antibodies ranged from 0.76 nM to 481 nM. Therefore, the cross-reactivity and promiscuity of proteins receptors may lead to critical challenges in sensing the specificity for on-site airborne pathogen detection. To improve the specificity and selectivity of epitope-based biosensors, strategies such as antifouling layers and smart assay design can be generally applied as discussed in previous works.^228^ Furthermore, rapid pretreatment following bioaerosol collection is required to remove potential interfering substances in practical on-site airborne pathogens detection. These strategies will be discussed in detail later (Sections 5.3 and 5.4).

### 5.2 On-site bioaerosol sensing strategies

Selective biosensing receptors targeting pathogen recognition sites require sensitive transducing elements to transform the molecular interactions into quantifiable analytical signals. These transducing elements can be a photo-responsive matrix, a nanomaterial-based electrode, or an interface of a piezoelectric microbalance. Therefore, based on the different physiochemical transducing principles, biosensors can be readily classified into several different groups, namely, electronic/electrochemical, optical, and mechanical/acoustic biosensors. Hereby, we review the novel progresses of high-performance biosensing systems for on-site airborne pathogen detection and further provide insights following the “*SARSVAC*” criteria. The potential strategies and novel designs for improving the bioaerosol sensing performance are summarized in Table 3.

**Table 3:** Potential strategies to improve the biosensing performance under the scheme of “*SARSVAC*” criteria for on-site airborne pathogen detection and risk assessment

		“*SARSVAC*” criteria for on-site airborne virus detection						
		*S*	*A*	*R*	*S*	*V*	*A*	*C*
		Sensitivity	Accuracy	Robustness	Swiftness	Versatile	Automation	Connectivity
Electrochemical devices		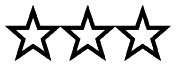	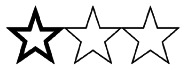	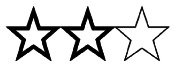	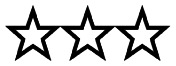	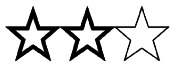	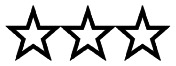	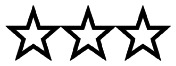
	Strategies	• Labelling	• Antifouling	• Microelectronic circuit	• Using high affinity receptors	• CMOS sensor array for multiplexing detection	• Electronic controlling system for sampling, delivery, and biosensing	• Interfacing with established electronic read-outs
		• Chemical amplification	• Highly stable electronic unit	• Robust semiconductor manufacturing techniques (SMT)	• Improving mass transfer by microfluidic design	• Multichannel microfluidics	• Digital microfluidic system	• Wireless communication unit
		• 2D materials	• Lock-in amplifier	• Functional surface	• Conductive nanofibers for *in situ* detection	• Multi-sensors integration		• Smartphone based readout
			• Hydrosol sample pretreatment					

Optical devices		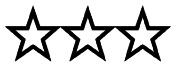	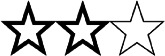	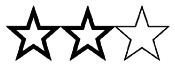	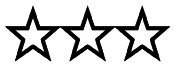	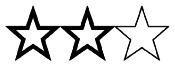	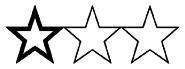	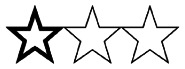
	Strategies	• Enhanced light-matter interactions	• Self-reference design with interferometry	• Optofluidic system	• Thermoplasmonic enhanced mass transfer	• Multiplexing using imaging sensors	• Integrated optics	• Optoelectronic unit for fast interfacing
		• Labelling	• Unique fingerprints with Raman spectroscopy	• Integrated optics	• Improving microfluidic design	• Multichannel with different surface functionalization	• Optofluidic units for manipulation	• Distributed sensors networks
		• Novel optical modulation		• Robust optical sensing materials		• Multiplexing with wavelength	• Autonomous microfluidic system	• Hybrid radio/optical sensors
		• Sensitive optical materials						• Smartphone-based detection

Mechanical devices		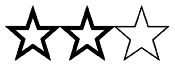	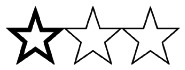	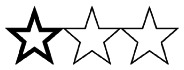	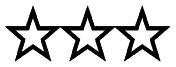	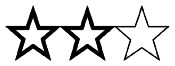	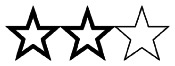	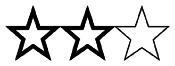
	Strategies	• Labelling	• Antifouling	• Preanalytical sampler preparation	• Nanoelectromechanical units	• Mass multiplexing of the bio-MEMs functionalization	• Automated microfluidic unit	• Wireless communication unit
		• Sensitive readout approach (EC)	• Multichannel detection	• Robust mechanical materials	• Improving microfluidic design	• Multichannel detection	• Microelectromechanical controlling system	• Interfacing with established electronic read-outs
		• MOFET-embedded microcantilever	• Self-reference design					

#### 5.2.1 Electronic and electrochemical pathogen sensing methods

**Fig. 14:**
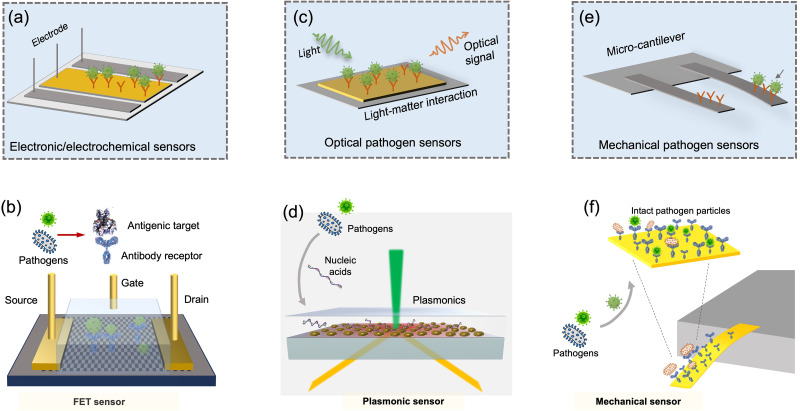
Airborne pathogens can be rapidly and sensitively detected by electronic/electrochemical, optical/photonic, and mechanical biosensors. (a) The electronic/electrochemical pathogen sensing system use the conductive electrodes to probe the electrical alterations caused by capturing pathogen target. (b) Schematic demonstration of FET-based electronic/electrochemical viral sensor. 2D materials such as graphene can be utilized as a transducing electrode material. Antibody receptors can be conjugated onto the substrate for detecting different pathogens or their antigenic epitopes. (c) The photonic or optical pathogen sensors transduce the optical signal alterations based on light matter interaction such as absorption, scattering, reflection, and non-linear emissions. (d) Schematic illustration of LSPR-based optical viral sensor. Gold nanoisland (AuNI), as a plasmonic enhanced light–matter interaction matrix, was utilized to manipulate and transduce biomolecules. The complementary DNA probes are conjugated onto the AuNI matrix to detect the nucleic acid materials of targeted pathogens. (e) The mechanical sensors use the micromechanical unit to probe the binding events. (f) Schematic illustration of miniaturized micromechanical sensors for detecting nanoscale airborne pathogen targets. This portable cantilever-based airborne nanoparticle detector (CANTOR) is a combination of a miniaturized sampler made of aluminum material and a cantilever resonator. Schematic ideas were followed as represented in previously published article ref. 44, 232 and 295.

In electronic/electrochemical biosensing systems (Fig. 14a), conductive electrodes are generally used in conjunction with electronic devices to probe the electrical alterations caused by capturing target pathogens or its fragments.^259^ Based on the transducing signals, electrochemical biosensing approaches can be divided into categories of amperometry (monitoring current over time by keeping the potential constant), conductometry (measuring conductance with a constant AC potential), voltammetry (gauging current under varied potentials), and potentiometry (measuring real-time potential under a constant electric current).^260,261^

Based on the fast development of low-cost microelectronic circuits and nanofabrication technologies, electrochemical-based biosensors demonstrate advantages of high robustness, low-cost, easy miniaturization, and low system complexity.^262^ Additionally, electrochemical biosensors can be easily integrated with existing sensor networks due to their easy interface with conventional electronic read-out.^263^ Field-effect-transistors (FETs) and miniaturized potentiometric systems are the prominent instances for airborne pathogen detection.^232,264^ Seo *et al.* proposed a graphene-based FET biosensor for detecting the SARS-CoV-2 virus in different liquid media (*e.g.*, buffer solvent, biological fluid, and culture medium), as shown in Fig. 14b.^232^ According to the preliminary biosensing results, the FET-based biosensor possessed a detection limit of 242 copies per mL through the direct recognition of the antigenic epitopes. In addition to this superior sensitivity, electrochemical biosensors also possess the advantages of easy-to-manufacture and integration. A split aptamer (SA)-based electrochemical sensor chip was recently developed for the rapid detection of adenosine triphosphate in bioaerosols with a detection limit of 10 pM, which can be used for the ubiquitous detection of total bioaerosols.^265^

Additionally, the transducing electrodes of electrochemical biosensors can be easily integrated onto different flexible or low-cost substrates (*e.g.*, paper, polymers, and clothes).^266–269^ For instance, the screen-printing method could enable the mass manufacturing of electronic pathogen sensing devices.^270,271^ Recently, a paper-based voltametric electrochemical biosensor was developed for rapid and on-site airborne influenza virus quantification.^17^ This voltametric biosensor had a fair detection limit of 2.13 PFU per mL. Due to the integrability of electrochemical systems, this low-cost and paper-based biosensor has been directly incorporated with a portable electrostatic aerosol concentrator for the continuous monitoring of virus-laden aerosols. In another recent work, Xue *et al.* integrated the conductive nanowire-based immunosensors, a miniaturized flexible impedance circuit and a wireless communication unit into an intelligent face mask for personal infection detection.^247^ Through the enrichment of bioaerosols by the facemask, this electrochemical biosensor could directly detect airborne coronavirus at a concentration of 0.35 PFU per L in air. By integration with aforementioned airborne pathogen collection, enrichment, and pretreatment units, as shown in Fig. 10, the electrochemical bioaerosol sensing systems have the potential to be employed for on-site airborne pathogen detection.

Although electrochemical biosensors offer a promising avenue for on-site airborne pathogen detection, their capability to detect trace amounts of specific target in complex aerosol matrix remains a major concern.^272^ The innumerable organic molecules, redox-active compounds, and biological fragments present in the aerosol samples may severely obstruct electrochemical biosensing performance by interfering with receptor activities, increasing background noise, passivating biosensing electrodes, and blocking biorecognition sites.^228,273^ Therefore, it is necessary to use surface antifouling and novel biosensing structure designs to further improve the biosensing robustness. General antifouling strategies have been systematically discussed in recently published articles.^272–275^ In addition, physicochemical interception in the sampling and pretreatment microfluidics could be another potential strategy to further purify and enrich the airborne pathogens or fragments *in situ*. For instance, the multi-layered filters can effectively remove insoluble particles before bioaerosols transduction.^17^ In addition, the conductive nanofibers (*e.g.*, CNTs) in a microfluidic system can be further developed into a multi-functional electrochemical system, in which the airborne pathogens can be simultaneously enriched (with physical or chemical interception) and *in situ* detected.^218^

#### 5.2.2 Photonic pathogen sensing methods

Photonic pathogen sensors present another promising approach for highly sensitive coronavirus detection (Fig. 14c).^276,277^ On the basis of optical modulating and transducing configurations, photonic biosensors can be divided into numerous classes, including optical resonance, dispersion, reflection, refraction, absorption, Raman scattering, and fluorescence. Utilizing nanomaterial-enhanced light-matter interactions, nanophotonic devices have demonstrated superior biochemical analysis performance for transducing trace amount of analytes.^276^ Among them, plasmonic enhanced light–matter interaction has proven to be an invaluable technology for the highly sensitive detection of pathogens such as bacterial and viral particles.^44,278^

Localized surface plasmon resonance (LSPR) indicated that photon-induced collective electrons oscillation resulted from the nanomaterial-enhanced light–matter interaction.^279^ Numerous LSPR-based biosensing systems, modulated *via* reflection, absorption, or phase, have been employed for detecting trace amounts of airborne pathogens.^18,44,280–282^ Moitra *et al.* proposed to use colloidal plasmonic nanoparticles for colorimetric viral gene quantification through rapid “naked-eye” detection.^282^ The same research group further integrated this plasmonic enhanced light–matter interaction technique into a hyperspectral sensor, which exhibited the hallmark of ultrahigh sensitivity at the yoctomole level and rapid turnaround time of a few seconds.^281^

In addition to improving the operability and sensitivity, smart optical sensors designs may significantly improve the biosensing reliability and robustness.^283^ A prominent example is using thermoplasmonics, an enhanced photothermal effect to regulate the thermodynamic interactions between target genes and oligonucleotide receptors, as shown in Fig. 14d.^44^ The proposed thermoplasmonic heating was able to increase the local temperature for nucleic acids recognition and facilitate the elimination of interference from nonspecific objects and spurious pathogenic genes.^44^ The same research group also developed a plasmonic biosensor for total bioaerosol detection.^284^ By leveraging the succinimidyl-ester surface functionalization, total bioaerosols can be quantitative detected with high sensitivity down to 0.5119 cells per mL.

Nanomaterial-based surface-enhanced Raman spectroscopy (SERS) is an alternative optical route to achieve highly selective and sensitive airborne species detection.^285^ Raman spectroscopic strategies are able to provide a unique spectral fingerprint generated by the interaction of light with the molecular vibrations of the target airborne pathogens.^286^ One research employed angiotensin converting enzyme 2 receptor-functionalized SERS biosensors to quickly detect viral spike proteins with a turnaround time of less than 5 min and a low LoD of 80 copies per mL.^287^ Additionally, the machine-learning algorithm was employed to improve the discrimination capability for identifying the pathogen-related SERS fingerprint in the complex liquid system. Choi *et al.* integrated an optofluidic SERS platform with a microfluidic-based aerosol-to-hydrosol impingement aerosol sampler and demonstrated the capability of continuous bioaerosols monitoring.^286^

Recently, optical biosensing assays based on photon emission have also been utilized for airborne virus quantification and risk assessment.^14,16,255^ Lee *et al.* described an integrated sampling-to-monitoring system for the on-site and real-time detection of airborne pathogens.^14^ The antibody-conjugated up-conversion nanoprobes (UCNPs) were able to specifically bind to the collected airborne pathogens in a paper-based system, and the stimulated UCNPs photoluminescent emission could be used for on-site and rapid virus quantification with LoD down to 10^4.294^ EID_50_ (50% egg infective dose)/m^3^. In addition, spark-induced plasma spectroscopy was also recently reported to achieve on-site and real-time detection of airborne viruses.^16^

There are also commercially available optical instruments for the real-time and on-site detection of airborne pathogens.^288^ One of these products utilized a cell-based and bioluminescent-mediated biosensing technology with membrane-bound pathogen antibodies and expressed bioluminescent protein collaborated to accomplish cellular analysis and notification of antigen risks and yields (CANARY).^255^ In addition to these established optical biosensing approaches, advanced photonic techniques such as chiral plasmonics, dielectric nano-resonators, and surface enhanced infrared absorption spectroscopy (SEIRA) could also be potentially utilized for highly sensitive on-site airborne pathogens monitoring.^289^

Optical and nanophotonic biosensors have offered high sensitivity, accuracy, and operational robustness for bioaerosols detection. To meet the “*SARSVAC”* criteria and achieve on-site risk assessment, the integrability and compatibility of optical biosensing devices should be further improved. For instance, 3D printed micro-photonic units could facilitate the miniaturization of bulky optical sensing systems. Moreover, optofluidics can be integrated with the micro-sampler units (Fig. 8) for continuous and uninterrupted pathogen-laden aerosols collection, pretreatment, manipulation, and detection.^290,291^ The integrated microfluidic controlling systems and optoelectronic elements could further enhance the automation and compatibility of optical pathogen-sensing systems.^220,292,293^

#### 5.2.3 Acoustic and mechanical pathogen sensing methods

The third major class of biosensors for airborne pathogen detection is micromechanical transducer, as shown in Fig. 14e, in which acoustic alterations in resonance frequency, acoustic velocity, and dissipation can be modulated for quantification.^294,295^ The miniaturized bio-microelectromechanical systems (bio-MEMS) with surface-functionalized bio-recognizing receptors (*e.g.*, aptamers, antibodies, and nucleic acid probes) exemplify a revolutionary technology for rapid and sensitive biochemical analysis.^296^

Recently, Agarwal *et al.* proposed a microcantilever-based bio-MEMS system for the rapid detection (<5 min) of viral spike and nucleocapsid proteins (Fig. 14f). By monitoring the nanomechanical deflections, it exhibited a high sensitivity of 100 copies per mL.^295^ In addition, the novel microelectronic readout units such as metal–oxide semiconductor FET (MOSFET) can be integrated into the micromechanical system to further improve the biosensing performance.^297^ In this novel mechanical bioaerosol sensing system, a microelectronic FET unit was embedded as the base of the micromechanical cantilever to directly read the deflections caused by the biomolecular binding.

To achieve on-site aerosol detection, a sampling unit, such as an electrostatic precipitator, has been further integrated with the micromechanical sensing system.^298^ In this miniaturized system, the resonating Si cantilever was parallelly employed as the electrostatic sampling electrode and the mechanical sensing matrix. By validating with 100 nm airborne particles, the sensor exhibited a mass sensitivity of 36.5 Hz ng^−1^ after 15 min aerosol sampling. By functionalizing specific protein receptors or complementary nucleic acid sequences on the micromechanical sensing substrate, the micromechanical system may be used as an integrated, rapid, and cost-effective system for on-site pathogen aerosol detection. Moreover, the miniaturized sensing volume and novel functionalization strategies also enabled multiplexed analysis with microelectronic biosensing system.^299^ Based on multichannel detection on the same platform, different pathogen units can be detected in parallel so as to eliminate the impact of non-specific binding events and provide more accurate bioaerosols analysis results. By supplying at least one reference channel, it is also possible to eliminate the background noise induced by the complex airborne components.^300^

Notably, despite the demonstrated ultrahigh sensitivity and versatile multiplexing applications, the robustness of bio-MEMS systems must be enhanced prior to their use for on-site airborne coronavirus detection.^300^ In addition, mechanical biosensing devices typically require additional signal reading units, which may have an effect on the system integration and compatibility.

### 5.3 Effective strategies to minimize non-specific aerosol interference

Aerosols represent complex chemical components in the air, mainly including metal ions, inorganic particles, organic carbon, and bioaerosols.^301^ The highly sensitive and selective detection of a trace amount of airborne pathogens in this diverse aerosol background remains challenging. In addition to improving the sensitivity, on-site bioaerosols detection demands effective strategies to minimize non-specific interference. In this section, we consider the main aerosol interference factors, including physical disruption from airborne particles, chemical inhibition of redox-active species, and biological interferences.

Sample pretreatment and direct size-dependent separations may be the simplest method for eliminating the impact caused by insoluble dispersed particles. Jang and co-workers proposed an on-site airborne pathogen biosensing system that integrated multilayer filtration pads with different pore sizes to purify the collected airborne pathogens and eliminate the interference from dust particles.^17,302^ Hong and co-workers developed a microfluidic inertial separator to simultaneously separate airborne pathogens by size.^205^ Airborne viruses, with dimensions less than 500 nm, can be purified and subsequently measured using a real-time analyzer. For size-based purification, active separation techniques employing acoustophoretic and dielectrophoretic techniques in a microfluidic system can also be utilized.^303^

**Fig. 15:**
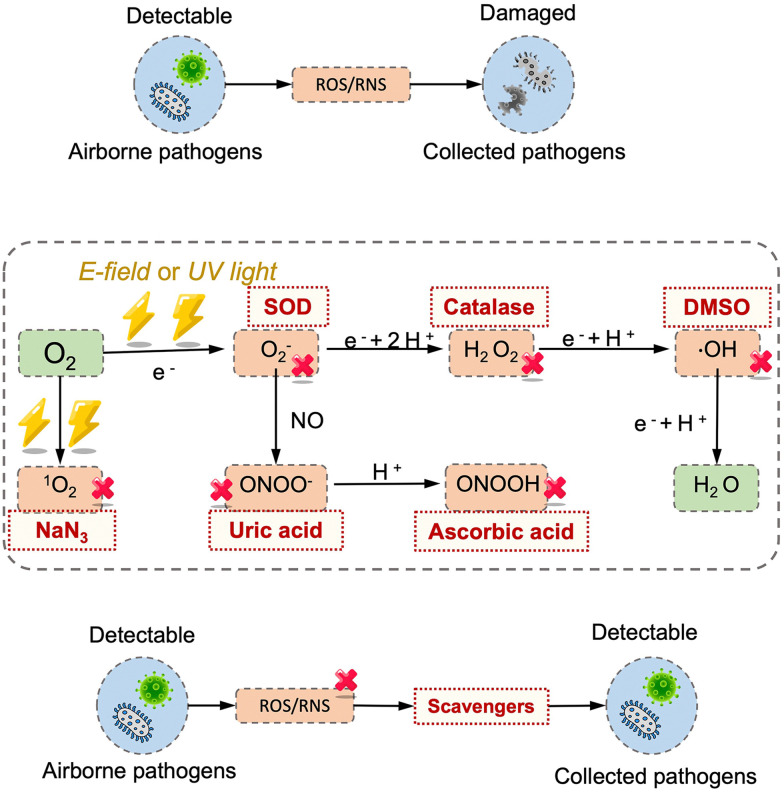
Scavengers with the potential to clean up redox-active species can minimize damage toward collected airborne pathogens and make them detectable in downstream-analysis. Sodium azide (NaN3), superoxide dismutase (SOD), catalase, dimethyl sulfoxide (DMSO), uric acid, and ascorbic acid can be used to reduce the negative impact on collected airborne pathogens.

Redox-active species, such as ROS, RNS, and transition metal ions, may inhibit the effective recognition of airborne pathogens by damaging the activity and functionality of recognition molecules.^173,174,304^ To this end, scavengers for redox-active species and radicals may be potentially used to reduce the oxidation of biomolecules and biosensing recognition sites.^181^ As shown in Fig. 15, Yamashiro and co-workers investigated the radical scavengers for ˙OH, ˙O_2_^−^, H_2_O_2_, ^1^O_2_, ONOO^−^, and ONOOH and found that NaN_3_ (scavenger for ^1^O_2_), uric acid (for ONOO^−^), and ascorbic acid (for ONOOH) could be good candidates to preserve the viability of collected airborne pathogens such as viruses.^173^ Using appropriate chemical scavengers in the aerosol-to-hydrosol sampling and bioaerosol elution process may preserve the pathogens as well as their protein structures and nucleic acid sequences, thereby enhancing the biosensing accuracy and preventing the underestimation of airborne concentrations.

Antifouling coatings represent one of the most useful chemical approaches. Readers could refer to these recently published articles, which have systematically summarized the antifouling technologies for biosensing applications.^229,272–274^ Intelligent biosensor design could be another significant strategy to tackle non-specific molecular interference. For instance, signal or molecular switch system and self-referenced transduction can effectively improve the bioaerosol sensing accuracy in a complex liquid matrix.^228^ The signal/biomolecular switch biosensing system is composed of functional proteins or nucleic acids that undergo conformational or functional changes upon interaction with the target pathogens. The distinct bioactivities of the biochemical switches, such as the cleavage activity of an enzyme or the photon emission of a bioluminescent protein, can be engaged when the biosensing targets contact the designated functional units.^305,306^ Based on the switches, non-specific molecules, even if bonded onto the biosensor surface, cannot activate the specific biofunctions, thereby avoiding the interference of complex non-specific background aerosols. As a type of robust functional biomolecules, CRISPR/Cas proteins can be used as enzymatic switches for detecting different pathogens. Additionally, site-specific restriction enzymes, such as nicking endonucleases, could also be employed to design biomolecular switch biosensors. For instance, endonuclease IV, which cleaves the apurinic/apyrimidinic (AP) sites in double-stranded nucleic acids, can be used as a biomolecular switch for airborne virus detection.^18,20^ This cleavage function is only activated when the pathogenic sequences are present and bind to the oligonucleotide probes, thereby avoiding false signals caused by non-specific binding events. Another prominent example of novel biomolecular switches is the *de novo* designed bioactive protein biosensors, as shown in Fig. 13, in which the target protein can switch the “closed” state of the protein cage to an “open” state to achieve highly-selective pathogen detection.^252,253^

While there are a growing number of promising developments of effective strategies for tackling non-specific aerosol interference with sample pretreatment, chemical scavengers, and intelligent biosensor designs, on-site biosensing implementation and robustness validation are still scarce. To further optimize the antifouling strategies and enhance the bioaerosol sensing robustness, more real-world implementation works including bioaerosol sample collection and on-site quantification should be conducted.

### 5.4. Integrated on-site airborne pathogen biosensing systems

**Fig. 16:**
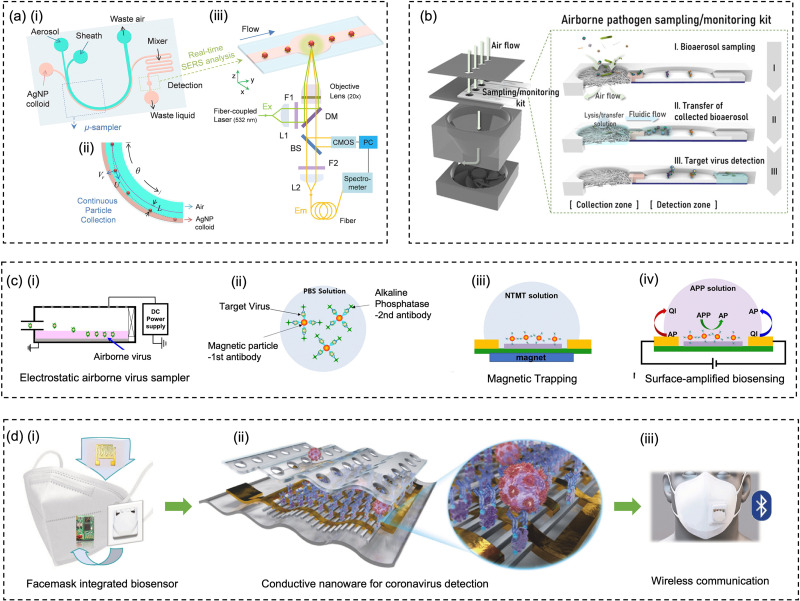
The collection of the fully integrated and rapid on-site airborne pathogens detection systems. (a) Microfluidic sampling system based on the inertia liquid impingement was used to collect the airborne pathogens and label with Ag nanoparticles. In a continuous manner, the AgNP-labelled pathogens can be rapidly detected by the SERS system. Reprinted with permission from ref. 286. Copyright (2020) Elsevier B.V. (b) Fully integrated sampling-to-biosensing platform for on-site and rapid airborne pathogen detection. The filtration collected bacteria or viruses were delivered to the NIR sensing zone through capillary motion. Reprinted with permission from ref. 14. Copyright (2020) American Chemical Society. (c) Airborne pathogens collected by an electrostatic sampler can be sensitively transduced by a reusable and surface-amplified electrochemical biosensor. Reprinted with permission from ref. 310. Copyright (2021) Nature Group. (d) An intelligent face mask integrated with high density conductive nanowire array and electrochemical biosensors for airborne pathogen detection. Reprinted with permission from ref. 247. Copyright (2021) Elsevier B.V.

From bioaerosols collection to the interpretation of the sensing results, airborne pathogens detection is generally carried out following specific steps in practical on-site and rapid infection risk assessment. This process mainly includes four parts: (1) aerosol sampling and enrichment, (2) pretreatment and delivery, (3) quantitative biochemical analysis, and (4) interpretation of bioaerosol sensing results. Some pioneering and recent research works reported the integration of different functional units for realizing on-site airborne pathogen detection in a continuous and real-time manner (Table S3, ESI†).^14,17,19,307–310^ Choi and co-workers developed a fully integrated optofluidic SERS biosensing platform for the simultaneous detection of airborne pathogens, as shown in Fig. 16a.^286^ The microfluidic-based liquid impingement sampler with the two-phase flow (*i.e.*, a sampled air flow and a collection liquid medium flow) was employed to collect the airborne bacteria. Colloidal silver nanoparticles (AgNPs) in the collection liquid could be directly adsorbed onto the target biomolecules in the microfluidic mixer and then enhanced the Raman spectroscopic signal in a continuous-flow manner. The sensitivity of this real-time and continuous bioaerosol sensing system was reported to be relatively low at 100 CFU per mL.

The combination of a filtration-based airborne pathogen sampling system and a lateral flow sample delivery system represented another instance of compatible and field-deployable airborne pathogen sensing system.^14^ This miniaturized sampling-to-monitoring device (Fig. 16b), proposed by Lee and co-workers, integrated three interconnected blocks: a filtration pad for aerosol particle filtration, a paper-based strip for sample delivery, and an optical sensing zone for up-conversion near-infrared (NIR) detection. Within 20 min, the collected airborne pathogens and antibody-conjugated nanoprobes can be efficiently enriched and detected by this miniaturized system. Using the avian influenza H1N1 virus as the virus-laden aerosol model, this platform achieved a detection limit at 10^4.294^ EID_50_ (50% egg infectious dose)/m^3^.

Another on-site airborne pathogen biosensing example, as shown in Fig. 16c, integrated an electrostatic precipitator and a surface-amplified electrochemical biosensing device.^310^ This electrochemical biosensing system utilized the magnetic nanoparticles and functionalized antibodies to capture the collected airborne viruses. By leveraging the external magnetic fields, these magnetic nanoparticles not only facilitated the virus enrichment on the electrochemical sensing surface but also erased all the biomolecules and pathogens after detection, thus achieving the reuse of the electrochemical sensor.

More recently, a condensation (hygroscopic growth)-assisted bioaerosol collection and plasmonic photothermal sensing (CAPS) system was developed for the on-site quantitative risk analysis of pathogen-laden aerosols.^20^ In addition to direct measurement of the airborne pathogen exposures with high spatiotemporal resolution, the biosensing signal can be translated to probabilities of infection risk and estimate maximum exposure durations to an acceptable risk threshold in different environmental settings.

Notably, it is found that the sensitivity of current on-site airborne pathogen biosensing platforms still needs to be further improved. Although detection sensitivity can be further improved through bioaerosol enrichment by extending the aerosol sampling duration, the potential physiochemical impact during the long sampling-to-biosensing process may also lead to a non-negligible loss of pathogen integrity and functionality. In addition, long sampling duration would deteriorate the time resolution of the biosensing system and jeopardize the capability for time-sensitive risk assessment. Some of the proposed pathogen aerosol sensing systems still require manual operations such as bioaerosol elution, pathogen lysis, and sample delivery.^310,311^ Therefore, in the follow-up work, the automation of the systems also needs to be further improvement. Regarding the capability of telecommunication and connectivity, Xue *et al.* proposed an integrated Bluetooth unit to directly upload the viral sensing results to the end-users (Fig. 16d).^247^ By further integrating the result interpretation unit and artificial intelligence (AI), the connectable on-site airborne virus sensing systems are poised to enable rapid infection risk assessment and evidence-based public health intervention. Another potential issue is that the majority of existing airborne pathogen biosensing systems cannot differentiate their viability and infectivity. Currently, there are several studies that utilized off-site and culture-based methods to investigate the viability of airborne viruses from different environments scenarios including patient wards and private car.^51,211^ There is still a high demand for the development of on-site biosensing technologies that can directly quantify viable airborne pathogens.

## 6 Amplification strategies for the detection of trace amount of airborne pathogens

Improving the sensitivity of detecting airborne pathogens can significantly enhance the reliability of transmission risk assessment and evidence-based public interventions.^18,41,310^ Therefore, there is a high demand for incorporating sensitivity improvement strategies into airborne pathogen sensing systems. One of the most effective ways is to amplify the biosensing response triggered by the presence of pathogen targets.^312,313^ In addition, the selective amplification of biosensing targets or signals could also diminish the interference caused by complex and diverse background components.^20^

Numerous amplification strategies have been proposed and implemented to enhance the sensitivity.^312,314–316^ This section focuses primarily on two essential physiochemical approaches, namely, (1) enzyme catalysis-based amplification approaches and (2) nanomaterial-based amplification approaches. These amplification techniques could be further developed into “plug-and-play” modules for numerous on-site airborne pathogens and bioaerosols analyses with enhanced sensitivity and robustness.

### 6.1 Enzyme-based amplification

Enzymatic and catalysis-based amplification strategies represent a fundamental concept for sensitivity enhancement, which leverages the chemical reaction at the biosensing step to amplify the transducing signal or the detection target. Enzyme-linked immunosorbent assay (ELISA) and polymerase chain reaction (PCR) are two examples that amplify the sensing signal and detection target, respectively, using enzymatic chemical reactions. Antibody-conjugated enzymes can be specifically immobilized onto the ELISA wells by recognizing epitopes and triggering a chemical reaction that converts the color of appropriate probing substances, such as 3,3′,5,5′-tetramethylbenzidine (TMB) or 2,2′-azino-bis(3-ethylbenzothiazole-6-sulfonic acid) (ABTS).^317^ While in a PCR system, the short complementary oligonucleotides primers can be elongated by the polymerase enzymes when they specifically bind to the target sequences. By repeating the hybridization–polymerization–dehybridization process, the biosensing targets can be rapidly replicated and thereby realize highly sensitive pathogen detection.^239^ Inspired by these two conventional amplification concepts, enzymatic and catalysis-based amplification strategies can be adapted for on-site airborne pathogen detection.^310,318–321^

#### 6.1.1 Amplification strategies of the pathogenic target

As highly specific biomarkers, nucleic acids of airborne pathogens are ideal replication targets for enzymatic amplification and achieving enhanced sensitivity and selectivity.^315,322^ PCR, as the ‘gold standard’ of detecting airborne pathogens, has demonstrated superior sensing reliability.^239^ However, the thermal cycling process with bench-top PCR devices limits its applicability in the field of rapid on-site coronavirus quantification. In contrast, isothermal amplification methods such as recombinase polymerase amplification (RPA), rolling circle amplification (RCA), and loop-mediated isothermal amplification (LAMP) can be alternative candidates for rapid and on-site airborne pathogen sensing applications.^315,323–327^ Ganguli and co-workers demonstrated an isothermal LAMP-based biosensing system for highly sensitive pathogens detection.^328^ Using a microfluidic-based isothermal amplification cartridge and a smartphone-based fluorescence readout system, this amplification-based system achieved a superior detection limit of 50 viral RNA copies per μL within 30 min. Jiang and co-workers integrated the LAMP isothermal amplification system into a hand-held device for rapid and on-site airborne virus detection.^41^ The pathogen-laden aerosols, collected by an liquid impactor, can be directly pre-treated (*e.g.*, collection, lysis, and RNA extraction) and isothermally amplified on a paper-based analytical system. The whole on-site sampling-to-biosensing process took only about 1 h and provided a detection limit of 1 TCID_50_ for the H1N1 influenza virus model.

**Fig. 17:**
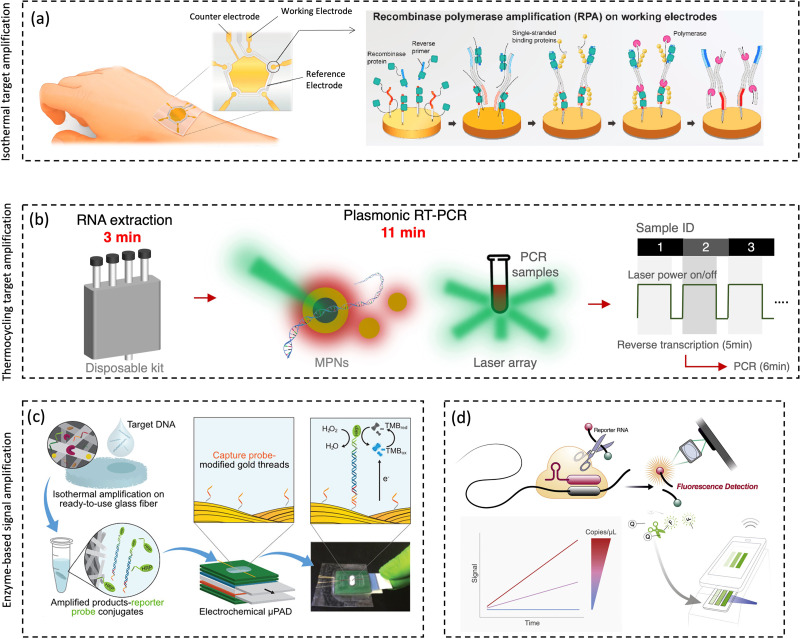
Enzyme-based amplification for sensitivity improvement. (a). Isothermal recombinase polymerase amplification (RPA) on electrochemical biosensor for detecting pathogenic sequences. The isothermal heating can be achieved using human body temperature. Reprinted with permission from ref. 319. Copyright (2021) Elsevier B.V. (b) Plasmonic Photothermal heating effect for rapid (∼11 min) thermocycling pathogenic sequence amplification and PCR-based fluorescence detection. Schematic ideas were followed as represented in previous published article ref. 331. (c) Enzyme-based signal amplification using nucleic acid linked horseradish peroxidase (HRP) and paper-based electrochemical biosensors. Reprinted with permission from ref. 341. Copyright (2021) American Chemical Society. (d) CRISPR/Cas13-based biosensing system for generating amplified fluorescent signal through the indiscriminative cleavage. The cellphone can be used as convenient and connectable device for signal reading. Reprinted with permission from ref. 225. Copyright (2021) Elsevier B.V.

By further incorporating isothermal amplification strategies and novel transducing techniques, the integrated airborne pathogen sensing system could further improve the biosensing performance and the integration level of the whole system. Kim *et al.* harnessed the voltametric electrochemical biosensor and isothermal RPA approach to rapidly detect the amplified viral targets with a detection limit of 1000 viral copies per μL.^319^ Moreover, taking advantages of the miniaturized microelectronic components, the on-chip RPA system can be operated using the human body temperature heating, as shown in Fig. 17a. This proposed biosensing system illustrated a feasible route for measuring personal airborne pathogen exposure.

In addition, the development of novel nanophotonic technologies also offers a convenient avenue for amplification-based bioaerosols detection. Plasmonic-enhanced photothermal effect can be utilized as a precise and efficient heating source to promote the enzymatic amplification in polymerase chain reaction.^329,330^ Instead of using conventional heating components such as Peltier-block heater, photonic PCR devices employed plasmonic photothermal effect to achieve ultrafast thermocycling and efficient nucleic acid amplification.^329^ As shown in Fig. 17b, Cheong and co-workers developed a portable nanophotonic-PCR device for detecting pathogens (*i.e.*, SARS-CoV-2) using plasmonic nanoparticles as an ultrafast heating source.^331^ This prototype could simultaneously measure three samples within 17 min and achieve a detection limit of 3.2 viral copies per μL. In 2021, Kang and coworkers further integrated the nanophotonic PCR system onto a microfluidic on-chip PCR platform.^332^ This miniaturized plasmofluidic PCR system was able to complete 40 thermocycles of amplification within 5 min. Therefore, by further integrating the microfluidic-based aerosol sampling and pretreatment system, this ultrafast and sensitive amplification strategies can be potentially used for on-site airborne pathogen detection and infection risk assessment.

However, the integration of enzymatic amplification strategies for airborne pathogen detection also faces challenges such as the interference from complicated aerosol background.^333^ The airborne redox-active species (*e.g.*, humic-like substance and polyphenol), metal ions (*e.g.*, calcium ions), and biological compounds (*e.g.*, protease) might inhibit the functionality of the enzyme and interfere with the quantification of the airborne viruses.^333,334^ Therefore, it is also important to incorporate effective strategies to further purify the pathogenic targets and remove the amplification inhibitors.^335–338^ For instance, the microfluidic-based bioaerosol purification strategies (Section 4.5) and chemical scavengers for removing airborne redox-active compounds (Section 5.3) can be utilized to ensure a stable and effective enzymatic amplification.

#### 6.1.2 Amplification strategies of readout signal

Beyond target-amplification options, a further interesting approach to increase the sensitivity of airborne pathogen detection is to amplify the biosensing readout signal through enzymatic and catalytic activities. Instead of using conventional benchtop readout systems, the signal amplification concept in ELISA can be further integrated with microfluidics and novel biosensors to further improve the sensing swiftness, sensitivity, and robustness.^310,339,340^ In addition, the highly specific nucleic acid probes could also be used to immobilize the oligonucleotide-linked enzymes for conducting ELISA-like signal amplification, as shown in Fig. 17c.^341^

CRISPR/Cas-based technologies represent another type of promising strategies for biosensing signal amplification.^342^ Hybridization between the pathogen sequence to Cas-crRNA strand ensures a high recognition specificity and subsequently activates the cleavage function of the Cas protein to amplify the biosensing signal by indiscriminately cleaving surrounding sequences (*e.g.*, RNA linked fluorophore-quencher pair).^343^ For instance, Fozouni and co-workers proposed a portable and smartphone-based CRISPR/Cas13 biosensing system for highly sensitive viral RNA detection (Fig. 17d).^225^ This CIRSPR biosensor achieved 100 viral copies per μL sensitivity with approximately 30 min turnaround time. Additionally, by harnessing a specific allosteric probe and CRISPR Cas13a component, an APC-Cas detection system was also developed to detect very low numbers of bacterial pathogens without additional isolation.^344^ It can selectively and sensitively quantify *Salmonella enteritidis* (from 1 to 10^5^ CFU) in various complex real-world samples.

Site-specific nucleic acid cleavage enzymes such as nicking enzymes and restriction endonucleases have also been utilized for biosensing signal amplification. These site-specific enzymes could selectively cleave designed probes *via* cyclic cleavage in order to accomplish “switch-on” or “switch-off”-based signal amplification.^18,345^ For instance, in a “switch-on”-based amplification system, the fluorophore-quencher pairs can be continuously processed by restriction endonuclease after hybridization with the virus sequences.^18^ The localized nanophotonic heating could facilitate the entire isothermal process for highly efficient signal amplification, including the dehybridization of cleaved short oligonucleotide and promotion of enzymatic reactivity. Compared to the non-amplification results with the same biosensing platform, this signal amplification strategies significantly improved the sensitivity by up to two orders of magnitude for airborne pathogen detection.

Target-triggered and polymerization-based signal amplification performed *in situ* on the transducing surface is considered as an alternative approach to magnify the readout signal and improve the bioanalytical sensitivity.^346,347^ Generally, the initiating molecules are conjugated to protein or nucleic acids probes, which can specifically recognize the pathogen targets and allow localized chemical reactions to subsequently proceed. Polymerization-based signal amplification can be achieved by harnessing a variety of different chemical reactions, including atom transfer radical polymerization (ATRP), fragmentation chain transfer polymerization (RAFT), ring-open metathesis polymerization (ROMP), and enzyme-mediated redox polymerization.^348,349^ Recently, Kim and co-workers reported a novel exponential signal amplification strategy based on the photo-initiated redox autocatalysis, wherein the photocatalyst (Eosin Y) amplified the biosensing signal by activating a nonfluorescent Eosin Y derivative (EYH^3−^) under green light illumination.^347^ Additionally, Eosin Y amplification was coupled with another photo-sensitive reaction, *i.e.*, the oxidative polymerization of 3,3′-diaminobenzidine (DAB), so as to yield different forms of signals and benefit the sensitive detection of antigenic epitopes of pathogens. Gurnani and colleagues have described an oxygen-tolerant RAFT polymerization platform based on a modified Fenton reaction that is initiated by the iron-reducing bacterial species *Cupriavidus metallidurans*.^350^ Although this RAFT method was proposed for producing well-defined polymeric materials, this concept also demonstrated high potential for highly sensitive viable pathogens detection.^351^

### 6.2 Nanomaterial-based sensing amplification

**Fig. 18:**
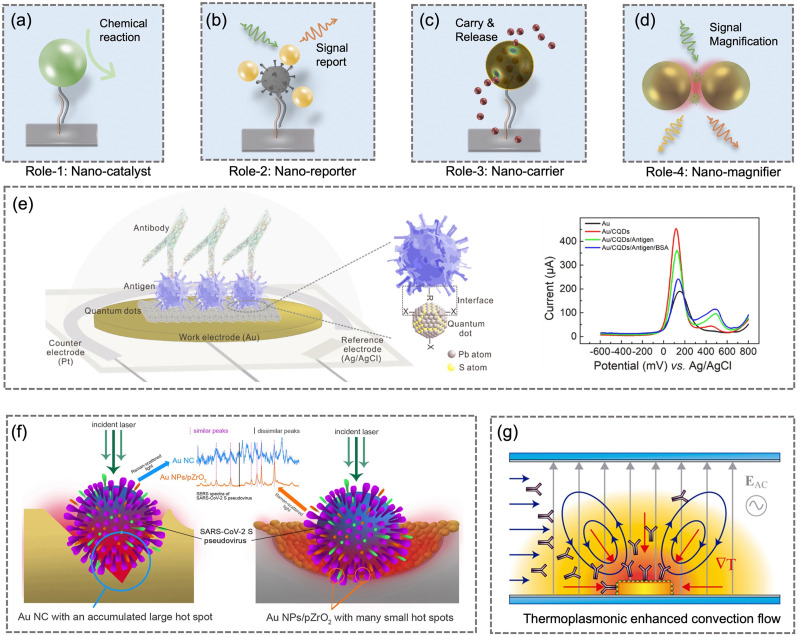
Nanomaterials are capable of amplifying the biosensing signal as (a) nano-catalyst, (b) nano-reporter, (c) nano-carrier, and (d) nano-magnifier. (e) Quantum dot, as a functional nanomaterial, was used to modify the work electrode and amplify the airborne pathogen transducing signal based on the quantum-enhanced carrier transportation. Reprinted with permission from ref. 361. Copyright (2022) Elsevier B.V. (f) AuNPs, as effective SERS nano-reporter and nano-magnifier, can effectively enhance the biosensing sensitivity based on the nanoplasmonic coupling effect. Reprinted with permission from ref. 365. Copyright (2022) Elsevier B.V. (g) Electrothermoplasmonic (ETP) effect based on plasmonic nanomaterials was used to overcome the diffusion limit through fluid convective flow generation and enhanced thermo-viscous particle motion. Reprinted with permission from ref. 366. Copyright (2018) American Chemical Society.

Nanomaterials are attractive candidates for signal amplification and sensitivity enhancement in novel pathogen biosensors due to their unique optical, electrical, and magnetic properties as well as enhanced reactivities. On the basis of amplification principles, nanomaterials for bioaerosol sensing amplification have been divided into four distinct classes: nano-catalysts (to initiate chemical reaction, Fig. 18a), nano-reporters (to generate secondary or enhanced signals, Fig. 18b), nano-carriers (to delivery probes and signals, Fig. 18c), and nano-magnifier (to enhance physical and chemical interactions, Fig. 18d).

Nano-catalyst, as a fruitful biosensing enhancement tool, is able to amplify the transducing signals and improve the sensitivity by introducing localized chemical reactions or initiating enzyme-mimicking activities (known as nanozyme).^352–354^ For instance, nanomaterials such as porous Au@Pt NPs, Pd@Pt NPs, Mn_2_O_3_ NPs, and Fe_3_O_4_ NPs have been used as effective peroxidases for catalyzing a chromogenic reaction, while some of them have been deployed for the amplification-based detection of airborne pathogens.^355–359^ Compared with biological enzymes, nanozyme demonstrated higher catalytic stability, better resistance to interference, ease of modification, and lower manufacturing cost.^354^ As a promising candidate, biosensing characteristics such as robustness, stability, and biocompatibility for the amplification-based detection of airborne pathogen should be further investigated.

Additionally, functional nanomaterials such as quantum dots (QDs) and upconverting nanoparticles (UCNPs) can be used as effective nano-reporters and nano-magnifiers to enhance the transducing signal or individually provide a secondary readout.^360,361^ For instance, based on the Förster resonance energy transfer (FRET) effect, the designed conjugation of functionalized QDs and AuNPs can be used to monitor the viral spike-ACE2 bindings.^362^ Apart from the direct observation of the fluorescence reaction generated by FRET, the amplified energy transfer signal can also be quantified by highly sensitive approaches such as LSPR and electrochemical sensors (Fig. 18e).^18,361,363^

Nanomaterials, which may possess unique optical or electronic properties, can be directly used as nano-magnifiers to amplify the biosensing signal. In particular, by enhancing light-matter interactions, nanoparticles can achieve enhanced Raman scattering (SERS) and fluorescence emission (SEF) at the biosensing surface (Fig. 18f).^364,365^ For example, colloidal silver nanoparticles can be utilized to capture airborne pathogens in a microfluidic system and subsequently employed as nano-magnifiers to amplify the Raman signal for the real-time quantification of bacteria such as *S. epidermidis*, *M. luteus*, *E. hirae*, *B. subtilis*, and *E. coli*.^286^ Meanwhile, the photothermal effect with plasmonic nanomaterials can also improve the biosensing performance by tackling the diffusion limit in localized biosensing surface.^283,366,367^ As shown in Fig. 18g, the immobilized nanomaterials were able to create a nanoscale temperature gradient and generate a thermo-viscous motion of particles. This physical process can effectively improve the mass transfer efficiency of nanoscale analytes such as the pathogens or their fragments, thereby enabling indirect signal amplification.

The in-depth investigation and discussions of how to use biological enzymes or nanoparticles to improve the biosensing performance have been presented in numerous research works recently.^314,323,328,347,352,354,368–370^ These solutions have the potential to be further developed as “plug-and-play” components for adaptable biosensing systems, thereby achieving higher sensitivity, selectivity, accuracy, and swiftness for bioaerosol sensing applications. Meanwhile, these integrated biosensing system could be employed as direct exposure measurement devices for the highly reliable risk assessment of airborne pathogen transmission. Compared with indirect modelling approaches, novel biosensors will play a crucial and complementary role in the research of bioaerosol transmission, public health intervention, and disease prevention.

## 7 Conclusions and perspectives

Bioaerosol researches have been conducted for decades. However, COVID-19 outbreak has once again brought to light the fact that bioaerosols and airborne pathogens play a critical role in the transmission of specific diseases. A majority of environmentalists, clinicians, and epidemiologists underestimated the potential of bioaerosol transmission in the early stages of the COVID-19 outbreak. This has forced us to reflect again on whether common bioaerosols also significantly impact our well-being and health condition more universally in daily lives, *e.g.*, by causing respiratory infection or sick building syndrome. This does require more systematic research works by researchers from different communities and aspects.

**Fig. 19:**
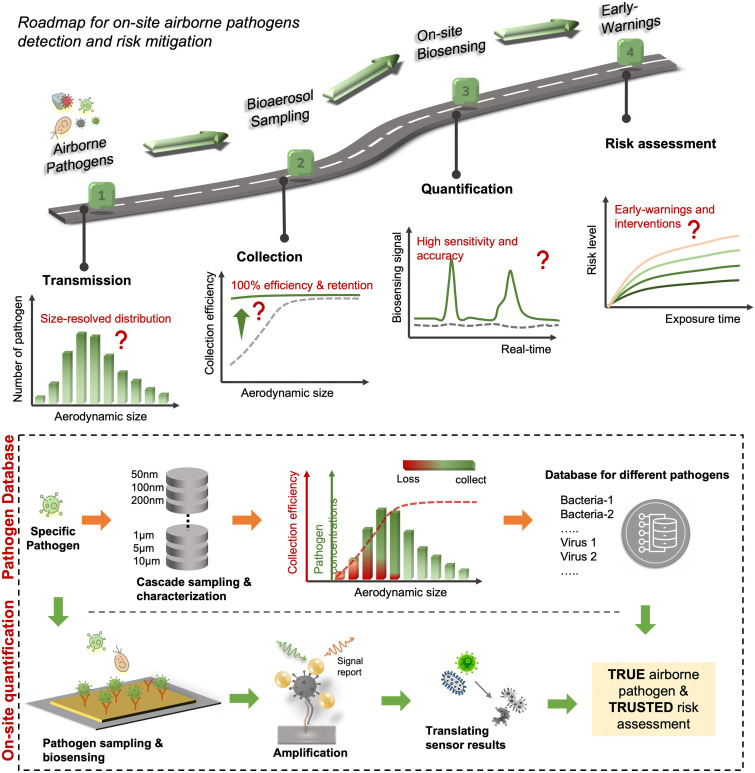
The schematic illustration of roadmap for identifying the main research priorities in the field of airborne pathogen transmission, bioaerosol sampling, on-site detection, early-warning, and risk assessments. Size-resolved transmission characterization, true concentration of airborne pathogens, highly sensitive detection and trusted risk assessment still remain as challenges. Establishing a pathogens database of dose response model and physiochemical characterizations for various airborne pathogens, as well as enhancing the efficiency of bioaerosol sampling and sensing, will be the key to resolving the current issues and challenges.

On-site airborne pathogen detection with novel bioaerosol sampling and quantitative analysis systems could provide a straightforward approach to achieve swift airborne pathogen exposure and transmission risk assessment. In this article, we summarized the crucial physicochemical properties that should be considered in on-site airborne pathogen detection. We also provided a thorough analysis of recent developments in the on-site pathogen-laden aerosol sampling and biosensing strategies with the consideration of these features. For instance, a miniaturized aerosol-to-hydrosol sampling device with a high ESEC performance can be employed for fast bioaerosol sampling. Subsequently, the downstream biosensing system could provide sensitive and reliable quantification results for estimating the airborne pathogen exposure and probability of infection risk. More importantly, by considering the requirements for on-site airborne pathogen detection and risk assessment, we also establish the “*SARSVAC”* criteria for evaluating the biosensing system, which means that the biosensing system should not only be Sensitive, Accurate, Robust, and Swift but also have the potential to be Versatile, Automated, and Connectable for implementation in distributed biosensor networks for the large-scale risk assessment of bioaerosols. Notably, the present review attempts to sketch the early stage of what is expected to become a long journey, as shown in Fig. 19. Specifically, realizing reliable on-site airborne pathogen detection and risk assessment requires concerted efforts from epidemiologists, bioanalytical chemists, air quality experts, material and data scientists. In the following two subsections, some perspectives and future opportunities are presented based on the current development status and challenges encountered in on-site airborne pathogen detection and early-stage risk mitigation.

### 7.1 Future challenges and opportunities in the on-site quantitative chemical analysis of pathogen-laden aerosols

To genuinely achieve the on-site quantification of trace quantities of contagious airborne pathogens, additional development under the “*SARSVAC*” criteria is required.

**Fig. 20:**
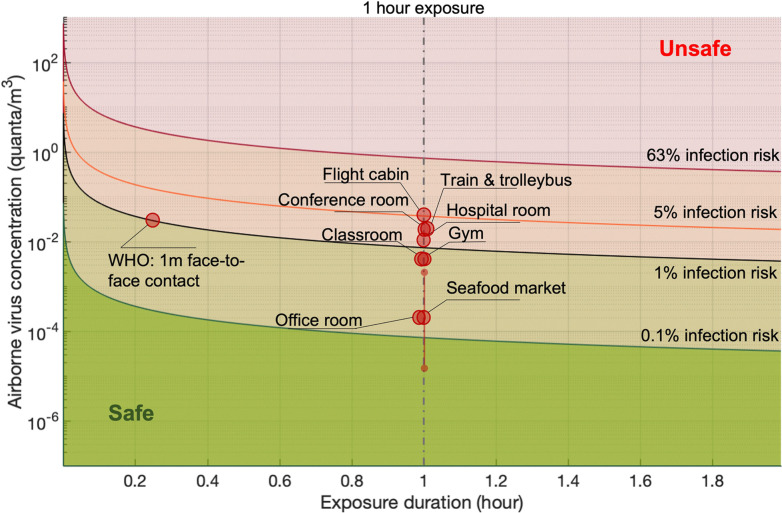
Risk diagram for estimating the infection probability based on the on-site biosensing results and calculated airborne SARS-CoV-2 concentrations. The different curves represent different infection risks, namely, 0.1%, 1%, 5%, and 63%. A higher infection risk (*i.e.*, higher virus concentrations and long exposure periods) implies an “unsafe” condition (red area), while a lower risk of infection (green zone) implies a “safe” state. Depending on the transmission scenario and population density, different thresholds (defining safe and unsafe exposures) can be set to determine the maximum exposure time at a specific pathogen concentration or the highest acceptable pathogen concentration for a specific period of exposure time. The red dots at 15 min exposure duration in the figure indicate the suggested reference scenario of 1 m face-to-face contact and its infection risk at 1%, while the other dots at 1 h exposure duration demonstrated the reported airborne SARS-CoV-2 concentrations estimated by simulation in different scenarios in the existing literature. The infection risk curve was calculated based on IR = 1.38 m^3^ h^−1^.

(1) Choosing an appropriate bioaerosol sampling strategy for subsequent detection. The possible loss and inactivation of the airborne pathogens may lead to severe underestimation in quantitative chemical analysis. Particularly, some bioaerosol sampling techniques, such as electrostatic and filtration-based samplers, may damage the functionality of the pathogen recognition sites, such as the nucleic acids or proteins (Fig. 15). These damages may cause a failure or underestimation in the downstream biosensing tests. Therefore, the potential damage to the airborne pathogens should be minimized using established or emerging biochemical strategies, thereby approaching an accurate and trusted quantitative bioanalytical result. In addition, given that pathogens may be further diluted to extremely low concentrations during airborne transmission, it may require extremely long air sampling times to accrue sufficient pathogens using low-flowrate bioaerosol collection systems, *e.g.*, microfluidic bioaerosol samplers. This indicates aerosol sampling techniques that are incapable of handling pathogen-containing air samples of several liters per min have a significantly reduced utility in many practical scenarios.

(2) Thoroughly considering the environmental impacts on the pathogen-laden aerosols during the transmission, sampling, and biosensing processes. The ideal “sampling-to-biosensing” procedure can be quick and conducted on-site. However, the airborne pathogens may still be significantly impacted by external environmental conditions such as RH, temperature, irradiation, aerosol pH, and airflow, leading to an underestimation of the transmission hazards. Accordingly, real-time environmental sensors can be deployed for monitoring ambient conditions such as temperature, irradiations, and RH (see Section 2.4) throughout the on-site bioaerosol sampling and transducing procedures. This allows for an estimation of pathogen degradation and inactivation.

(3) Improving the physical collection efficiency for nanoscale bioaerosols and considering the potential “penetration/escape window” in the pathogen-laden aerosol sampling. Pathogen-laden aerosols may be distributed in a wide aerodynamic size range from 20 nm to 0.2 mm (Fig. 5). Therefore, bioaerosol samplers with a large cut-off diameter may lead to significant pathogen loss. Meanwhile, some bioaerosol samplers may also demonstrate a U-shaped “escape window”, as shown in Fig. 7i. The intermediate airborne particles (with a diameter between 20 and 200 nm) have neither sufficient inertia to be efficiently collected by interception, gravitational, inertial impact mechanisms, nor intense Brownian motion to be collected by diffusion. Therefore, it is necessary to fully consider the loss in this “penetration/escape window” and further characterize or optimize the bioaerosol sampler.

(4) Considering the viability and infectiousness of airborne pathogens and developing on-site biosensing technology for effective bioaerosol risk management. Current biosensing techniques that rely on recognizing nucleic acids and protein molecules primarily quantify all airborne pathogens, including inactivated pathogens and their fragments. However, these detection results do not inherently correlate with the true risk of infection transmission because only viable pathogens can induce infection. Few biosensing technologies currently exist that can swiftly distinguish viable pathogens and characterize their infectiousness. Using cutting-edge biosensing technologies, quantitative viability and infectiousness results should be determined on-site as a crucial foundation for precise risk assessment.

(5) Investigating the concentration of pathogens in airborne particles as a function of aerosol size and their ability to initiate infection (Fig. 19). This size-resolved pathogen dose is a crucial metric for estimating the sampling loss, as discussed in item (3). To investigate the size-resolved distribution of specific airborne pathogens and the characteristics of long-distance airborne transmission, it is advantageous to develop size-selective bioaerosol samplers and highly-sensitive biosensors. In addition, bioaerosol risks correlated with the particle size and number concentration should be thoroughly investigated *via* direct biosensing measurements.

(6) Considering the ESEC of a chosen bioaerosol sampler. A high aerosol enrichment coefficient (ESEC) can enhance the biosensing swiftness and sensitivity for on-site airborne pathogens detection. Generally, ESEC can be improved by integrating high flowrate sampling units and miniaturized microfluidics. Additionally, a multifunctional microfluidic system can also be utilized as an effective method for rapidly pretreating and transporting the collected bioaerosols for quantitative chemical analysis.

(7) Improving the sensitivity and reliability of the biosensors by leveraging the amplification strategies. Highly sensitive airborne pathogen detection can be achieved by improving the intrinsic biosensor designs (Table S3, ESI†). This relies on highly specific biorecognition receptors, coupled with fast and intelligent transducing strategies. If a detection limit is higher than the quantity of pathogen in the collected sample volume, the selected biosensing technology is unsuitable for use in risk assessment. Using preanalytical microfluidic systems to enrich pathogenetic targets may be a time-consuming but still feasible routine. Meanwhile, nanozymatic and enzymatic chemical reactions can be incorporated into the novel biosensing systems to further enhance the airborne pathogen transducing sensitivity, as discussed in Section 6. The amplification process should be fast, efficient, reliable, and robust in magnifying the biosensing signal and quantifying the concentration. However, in practical applications, achieving efficient and stable signal amplification through chemical reactions still remain challenging because of the complex interference from airborne biochemical components. For instance, the dissolved ions may significantly impact the reactivity of enzymes, while the airborne redox compounds may inhibit the catalytic activity of the nanozyme. Consequently, the robustness of different amplification strategies should be further investigated and validated.

(8) Long-term utility and regeneration of biosensor systems for multiple measurements. Although many different biosensors have demonstrated excellent sensing performance for airborne pathogen quantification and risk assessment, many are still stuck with disposable cartridges for a single or few times of use. In consideration of the practical monitoring applications and sustainability of biosensors over extended periods of time, this could become a key barrier to widespread adoption. One way to improve the long-term utility that is suitable for certain airborne pathogen biosensors is to enable sensor-regeneration. Multiple approaches are potentially available for biosensor regeneration. In the literature, pH control is the most commonly used approach for the regeneration of antigenic epitopes-based biosensors. Altering the temperature and ionic strength as well as using strong detergents have been demonstrated to regenerate nucleic acid-based biosensors. However, one current challenge pertains to the absence of a clearly defined criteria for successful regeneration. Hence, it is difficult to assess the effectiveness of different regeneration procedures for detecting airborne pathogens. In addition, affinity-based bioreceptors possessing excellent binding affinity offer low detection limits for sensitive airborne pathogen quantification, which implies that the regeneration of the biosensors will be extremely difficult. Therefore, how to achieve high binding affinity to the pathogenetic analyte in a reversible fashion represents another critical challenge in the field.

(9) Developing a cost-effective and user-friendly on-site sensing system for connectable sensor networks. With the aid of wireless communication and IOT technologies, low-cost and user-friendly biosensors could be deployed in numerous locations, covering potential hotspots and forming high-density networks to provide the real-time mapping of the airborne pathogen distribution. The extreme version would consist of personal bioaerosol sensors that individuals can carry and use to monitor the bioaerosol exposure in their immediate vicinity, allowing for personal risk mitigation. IoT bioaerosol sensors have the potential to revolutionize the monitoring and management of bioaerosol exposures. As technology continues to evolve, these sensors should be made more intelligent and affordable, making them available to a wider range of individuals.

(10) Translating the biosensing results to potential infection risks and health impact. The concentration of airborne pathogens or bioaerosols detected by an on-site biosensing system can be used to estimate the probability of individual risk. Fig. 20 illustrated translated infection risk of COVID-19 infection based on different concentrations of airborne virus. By measuring the exposure level of individuals to airborne pathogens in different environmental settings, the probability of infection risk (*e.g.*, 0.1%, 1%, 5%, and 63%) can be estimated. The World Health Organization (WHO) suggested that face-to-face contact with a case within 1 m and for >15 min can be identified as a close contact and having a potential risk of infection.^371^ Based on the metanalysis results, the infection risk (probability) for this reference scenario can be estimated to be 1%.^136^ In addition, the airborne virus concentration based on the indirect modeling approaches (Table 1) were also plotted in Fig. 20. It is worth noting that the concentrations obtained by modeling-based approaches usually have a large uncertainty of 2–3 orders of magnitude. Therefore, utilizing on-site biosensing techniques or combining both modeling (indirect risk assessment) and measurement (direct risk assessment) techniques can result in a more precise risk management.

(11) Estimating the uncertainties and errors in on-site airborne pathogen detection. The majority of current biosensing methods for measuring ambient pathogen concentration were based on recognizing nucleic acid sequences or antigenic proteins, without considering pathogenic infectivity and viability. Risk assessment based on these results may lead to the overestimation of airborne pathogens concentration and infection risk level. Therefore, future research should incorporate biosensors that can differentiate the infectivity and viability of airborne pathogens in order to accurately assess the amount of viable pathogens as opposed to the total number of nucleic acid copies or protein concentrations. These uncertainties caused by the diversity of pathogen infectivity and viability should be fully considered in subsequent risk assessment.

### 7.2 Future challenges and opportunities in risk assessments and transmission risk mitigation

On-site exposure measurement and evidence-based infection risk assessment are growing to be an important field for investigating the health impact of airborne pathogens. An accurate on-site bioaerosols detection system not only bridges the gap in the conventional understanding of airborne pathogen spreading but also provides a promising avenue for swift public health intervention and decision-making. Despite their availability, on-site airborne pathogen detection and quantification methods have not been widely deployed in field applications due to technical or expense considerations. Herein, we further discuss the future development opportunities and challenges for evidence-based risk assessment.

**Fig. 21:**
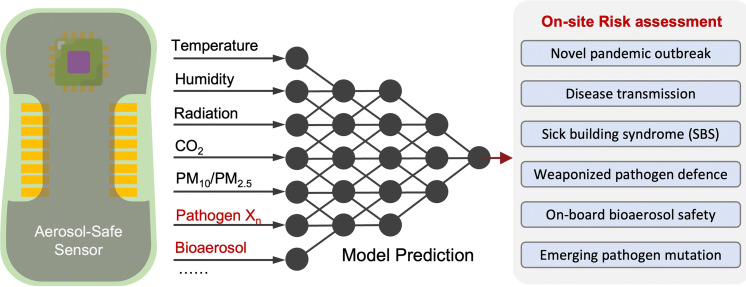
Schematic illustration of multiplexing biosensing frameworks with AI-assisted decision-making unit for the on-site transmission risk assessment of airborne pathogens.

(1) Optimizing the emission and exposure model through direct on-site measurement. Currently, epidemiological information from previous infection cases is generally utilized for estimating model-based bioaerosol emissions and individual exposure. The individual differences, such as pathogen shedding and the bioaerosols emission, are generally overlooked in the model-based methods and therefore cause high uncertainties. This explains why the simulated pathogen exposure and the actual measured values can vary by one to two orders of magnitude. The on-site airborne pathogen detection can be used as a direct verification technique to further optimize the models and the calculation parameters (*e.g.*, pathogen inactivation rate, aerosol deposition rate, and quanta conversion factors), thereby improving the accuracy of the model-based exposure assessment.^20^

(2) Optimizing the dose–response model through direct on-site airborne pathogen measurement. On-site airborne pathogen detection with biosensors can provide accurate exposure information with high spatiotemporal resolution for a specific infection case. By calculating and fitting the infection correlation, a pathogen-specific dose–response model can be effectively retrieved. With further validation, this on-site airborne pathogen measurement approach may become a viable alternative to the human-challenge (deliberate human infection) approach. In addition, establishing a reliable and accessible database for dose–response models of numerous airborne infectious diseases is also essential. The ID_50_ values of a number of pathogens have been collated and reported in this review (Fig. 1 and Table S1, ESI†), but further systematic and thorough validation is still urgently needed.

(3) Employing a biosensing system with a high spatiotemporal resolution to investigate the aerodynamic nature of airborne pathogens. Currently, the majority of cases of airborne pathogen transmission have been discovered through an indirect method by ruling out other fomite- or contact-based infections. The absence of direct on-site biosensing instruments and straightforward aerodynamic observations for airborne pathogens is one of the most importance factors. By deploying on-site biosensors for swift and real-time bioaerosol detection, the aerodynamic properties of pathogen-laden aerosols can be more accurately characterized. To accomplish this goal, however, further development in improving the sensing performance and integration of bioaerosol sensing systems is still highly demanded.

(4) Directly calculating the infection risk or bioaerosols impact using the biosensing results and advising the maximum exposure times for particular situations with a potential airborne pathogen presence. Though many integrated “sampling-to-biosensing” systems have succeeded in on-site bioaerosols and airborne pathogens quantification, the biosensing results have not been translated into an interpretable risk level and utilized for epidemic management. For instance, the high spatiotemporal airborne pathogen concentration measured by biosensors can be potentially used to estimate maximum tolerable exposure durations in different risk scenarios. Based on the bioaerosol sensing results, a recent work reported that the maximum exposure time in a high-risk COVID-19 patient ward was estimated to be about 46 min for a susceptible individual wearing an FFP2 facemask and engaging in low-intensity activity (inhalation rate at 1.38 m^3^ h^−1^).^20^ In future work, the integrated on-site bioaerosol sensing system and networks can be used to estimate personal infection probabilities, occupancy limit, and maximum exposure period in an indoor environment.

(5) Combining the direct on-site airborne pathogen measurement method and the indirect exposure modelling approach for smart decision making. A highly accurate on-site biosensing system could provide a straightforward pathogen exposure information. However, it may still be unaffordable or impractical for large-scale biosensing network. Indirect modelling approach could be more cost-effective and affordable. However, inter-individual differences on pathogen emission are generally not considered. Therefore, to carry out an effective risk assessment for transmission mitigation, it is essential to investigate how to incorporate both direct measurement and indirect modelling methodologies in an integrated smart sensing system. The optimization of risk assessment should consider more significant factors, such as vaccination and health status.

(6) Predicting infection, disease severity, and morbidity using a distributed on-site airborne pathogen sensing network. As a crucial spreading route for many infectious diseases, the concentration or emergence of airborne pathogen can be used as an effective factor to predict infection, disease severity, morbidity, and hospitalization rates. Direct measurement findings and a bigdata-based forecasting system could assist in medical facilities match potential patients with the proper degree of care capacity in order to more effectively manage their limited medical resources amid a spike in infection cases. As a result, the research priority may be used to further interpret the results of on-site and real-time airborne pathogen sensing from various locations, which may call for the combined efforts of epidemiologists, analytical chemists, data scientists, health care providers, and experts on air quality.

(7) Developing a decision support system by integrating artificial intelligence (AI) and an on-site pathogen sensing system (Fig. 21). AI decision-making system refers to the use of AI algorithms to analyze the data obtained from bioaerosol sensors and make decisions for risk mitigation. Biosensors can be used to monitor airborne pathogen concentrations, personal breath conditions, and other physiological parameters during exposure. Based on the epidemiological data, an AI-mediated system can analyze the data to provide insights into the transmission risks and suggest an adjustment to public health intervention. In recent years, integrating risk assessment with optical biosensors to facilitate risk management and information sharing has been implemented in healthcare settings.^20^ If AI-based decision-making systems can be incorporated into networked bioaerosol sensors for intelligent and automated risk assessment, a more credible and trustworthy early-warning system will be realized.

In conclusion, we strive with this review not only to provide comprehensive knowledge and insights about the recent advancements in the on-site airborne pathogen biosensing but also to further stimulate more in-depth reflection and research works in related fields. In recent years, significant progress has been made in understanding airborne transmission routes and developing biosensing technology, particularly in the areas of point-of-need detection and environmental monitoring. However, there is still considerable room for improvement in integrating rapid bioaerosol samplers and conducting on-site risk assessments for different types of airborne pathogens or bioaerosols. As mentioned, this review article only provides a snapshot for an early stage of what is expected to become a long scientific journey in on-site bioaerosol assessment. We look forward to the advancements in on-site airborne pathogen sensing and their applications in early-stage risk assessment. We intend for this review article to provide a roadmap for identifying the main research priorities and purposefully advancing on-site biosensing technologies. More significantly, the reviewed pathogen-laden aerosol sampling and biosensing technologies could also provide a promising avenue for conducting critical bioaerosol risk assessment and protecting the public health especially in on-site bioaerosols measurement scenarios such as the monitoring of high-risk pathogens at the check-in point of airplanes or cruise ships, defending weaponized pathogen aerosols, screening influenza variants at hospitals or other healthcare settings, and monitoring sick building syndromes at different workplaces.

## Conflicts of interest

There are no conflicts of interest to declare.

## Supplementary Material

CS-052-D3CS00417A-s001
