# Supplementary material for: On-site airborne pathogen detection for infection risk mitigation
Source: Chem Soc Rev. 2023 Oct 26;52(24):8531–79. doi: 10.1039/d3cs00417a (PMC10712221; doi:10.1039/d3cs00417a)
Supplement: CS-052-D3CS00417A-s001 [file CS-052-D3CS00417A-s001.pdf]

Electronic Supplementary Material (ESI) for Chemical Society Reviews.
This journal is © The Royal Society of Chemistry 2023

Supporting Information

On-site Airborne Pathogen Detection for Infection Risk Mitigation

Guangyu Qiu[a,b,c*], Xiaole Zhang[b,c], Andrew J. DeMello[d*], Maosheng Yao[e], Junji Cao[f], and Jing Wang [b,c*]

[a.] *Institute of Medical Robotics, School of Biomedical Engineering, Shanghai Jiao Tong University, Shanghai 200240, China*

[b.] *Institute of Environmental Engineering, ETH Zürich, Zürich 8093, Switzerland.*

[c.] *Laboratory for Advanced Analytical Technologies, Empa, Swiss Federal Laboratories for Materials Science and Technology, Dübendorf 8600, Switzerland.*

[d.] *Institute for Chemical and Bioengineering, Department of Chemistry and Applied Biosciences, ETH Zürich, Vladimir-Prelog-Weg1, Zürich, Switzerland.*

[e.] *State Key Joint Laboratory of Environmental Simulation and Pollution Control, College of Environmental Sciences and Engineering, Peking University, China.*

[f.] *Institute of Atmospheric Physics, Chinese Academy of Science, China.*

Table S1. Airborne pathogens that cause human infection. The microorganism feature and dose response models of airborne pathogens were collected for infection risk assessment.

Pathogens	Type	Microbiology	Hosts	ID50 or LD50	Dose response model	Bioaerosols transmission
Influenza (multi-subtypes)	Virus	80-120nm in diameter, Enveloped ssRNA virus	Human and animal	ID_{50} = 9.45E+05 (Viral particles)	Beta-Poisson a = 5.81E-01; N_{50}=9.45E+05	Respiratory activity
SARS-CoV	Virus	80-120nm in diameter, Enveloped +ssRNA virus	Human and animal	ID_{50} = 2.82E+02 (PFU)	Exponential, k = 2.46E-03	Respiratory activity, fecal-aerosol
SARS-CoV-2	Virus	80-120nm in diameter, Enveloped +ssRNA virus	Human and animal	ID_{50} = 2.27E+02 (PFU)	Exponential, k = 3.05E-03	Respiratory activity, fecal-aerosol, aerosolized wastewater
Adenovirus	Virus	70-100nm, nonenveloped dsDNA virus	Human and animal	ID_{50} = 1.14E+00 (viral particles)	Exponential, k = 6.07E-01	Fecal-aerosol, aerosolized wastewater
Norovirus	Virus	27-30nm, nonenveloped +ssRNA virus	Human and animal	ID_{50}=1.32E+03 (copies)	Exponential, k = 5.25E-04	Aerosolized wastewater and vomit
Rotavirus	Virus	70nm， nonenveloped dsRNA virus	Human and animal	ID_{50}=6.17E+00 (Viral particles)	Beta-Poisson a = 2.53E-02; N_{50}=6.17E+00	Fecal-aerosol, aerosolized wastewater
Rhinovirus	Virus	30 nm, nonenveloped ssRNA virus	Human	ID_{50}=1.81E+00 (Viral particles)	Beta-Poisson a = 2.21E-01; N_{50}=1.81E+00	Respiratory activity
Salmonella Derby	Bacteria	Rod-like, 2-5 µm long	Human and animal	10 CFU	Exponential, k = 6.93E-02	Fecal-aerosol, aerosolized wastewater,
Streptococcus pneumoniae	Bacteria	Spherical-shaped bacterium, 0.5 to 1.25 µm in diameter	Human and animal	10^4 CFU	Exponential, k = 6.93E-05	Respiratory activity, inhalation of aerosolized wastewater
Bacillus anthracis	Bacteria	rod-shaped, 1 to 1.2 µm in width and 3 to 5 µm in length	Human and animal	LD50=4.2E+04 (colony-forming units, CFU)	Exponential, k = 1.65E-05	Inhalation of airborne spores
Escherichia coli	Bacteria	rod-shaped, 2.0 µm in length and 0.5 µm in diameter	Human and animal	ID_{50}=2.11E+06 CFU	Beta-Poisson a = 1.55E-01; N_{50}=2.11E+06	Fecal-aerosol, aerosolized wastewater
Legionella pneumophila	Bacteria	rod-shaped, 0.3 -0.9 µm in width and 2 -20 µm in length	Human and animal	1.16E+01 CFU	Exponential, k = 5.99E-02	Inhalation aerosolized water
Neisseria meningitidis	Bacteria	oval-shaped, 0.6-1.0 µm in diameter and 1.5-2.5 µm in length	Human	10 CFU	Exponential, k = 6.93E-02	Respiratory activity and respiratory secretions; aerosolized water droplet.
Mycobacterium avium	Bacteria	Rod-shaped, 1-4 µm long and 0.2-0.5 µm in width	Human and animal	1000 CFU	Exponential, k = 6.93E-04	Aerosolized water droplet and dust particles.
Mycobacterium tuberculosis	Bacteria	Rod-shaped, 2-4 µm long and 0.2-0.5 µm in width	Human	10 CFU	Exponential, k = 6.93E-02	Exhalation aerosols from infected persons
Aspergillus versicolor	Fungi	flask-shaped, 150-300 µm in length and 4-6 µm in width	Human	N.A.	N.A.	Emission of spores from the fungus, aerosolized dusts, air conditioning systems
Aspergillus niger	Fungi	spherical-shaped bacterium, 2-4 µm in diameter	Human and animal	N.A.	N.A.	Aerosolized particles from soil or decaying vegetation; bronchoscopy or surgery in healthcare settings, air conditioning systems
Penicillium citrinum	Fungi	lemon-shaped, 2-3 µm wide and 4-6 µm long	Human	N.A.	N.A.	Emission of spores from the fungus, aerosolized dusts, air conditioning systems
Penicillium spinulosum	Fungi	globose or ellipsoidal in shape, 2-4 µm in diameter	Human and animal	N.A.	N.A.	Aerosolized particles from soil, dust and decaying vegetation

Approximate Beta-Poisson dose response model is specified as:

$$P_I(d) = 1 - (1 + \frac{d}{\beta})^{-a} = 1 - (1 + d\frac{2^{\frac{1}{a}} - 1}{N_{50}})^{-a}$$

where d is the infection dose, N_{50} is the median effective (mean) dose level. a is the optimized parameter for calculating beta-Poisson infection probabilities.

Approximate Exponential dose response model is specified as:

$$P_I(d) = 1 - e^{(-k \times d)}$$

where d is the infection dose, k is the optimized parameter for calculating exponential infection probabilities.

Table S2. Representative high exposure environments and reported airborne pathogen transmission cases.

Potential sites for on-site detection	Emission activities	Pathogens	Exposure scenario and periods	Reported infection cases	Airborne virus concentration or quanta emission rate	Ref.
Hospital	Breathing and brief/short communication at light activity	Virus (SARS-CoV-2)	10~15 minutes (with face masks) in a typical medical room (15m^2)	A cluster outbreak in a children's medical center. 9 individuals (6 HCWs, two children and 1 mother) were infected	No measurement results available. Other studies suggested a concentration range 1600~5700 copies/m^3.	1
	Breathing, talking	Virus (SARS-CoV-2)	>15 min exposure for HCWs in the patient ward	A cluster outbreak from one patient to 38 staffs and 14 patients	No measurement results available.	2
	Airborne no clear source (humidity issue found)	Fungus (Aspergillus fumigatus)	Patient long-term exposure in hospital ICU,	Patient infection in hospital ICU	Airborne pathogen concentration reported to be 16-640 CFU/m^3	3
	Ventilating air	Bacteria (L. pneumophila)	Exposure lasts for hours and days	Outbreak with 44 infectee including patients, employees, visitors, or passers-by	No measurement results available.	4
Hotel	Breathing	Virus (SARS-CoV-2)	Airborne transmission across the corridor of two hotel room	A fully vaccinated traveller staying in a hotel room across the corridor was infected from the index patient	No measurement results available.	5
Restaurant	Breathing, speaking at a light activity	Virus (SARS-CoV-2)	Restaurant room of 431 m^3 in volume and 145 m^2 in area. Air exchange rate 0.67 h^{-1}.	A cluster infection occurred at adjacent tables (with 1 m distance). Exposure time: 53~73 min	With a low ventilation rate, the quanta emission rate is estimated to be 79.3 quanta/h.	6
Public transportation: bus	Breathing (no speaking)	Virus (SARS-CoV-2)	60.42 m^3 in Bus-1 with 200min exposure, and 21.69 m^3 in Bus-2 with 60 min exposure	Ten passengers were infected on two buses by an index case. 7/46 and 2/17 infection reported respectively.	Simulated concentration with tracer gas: 603.2 ppm and 251.7 ppm in bus-1 and bus-2 respectively.	7
Public transportation: aircraft	Breathing and coughing (other routes could not be ruled out)	Virus (SARS-CoV-2)	Passengers in adjacent seats (business class). Total flight period was 10 hours.	A cluster of 16 infection cases by a symptomatic passenger.	No measurement results available.	8
School	Breathing, speaking and activates	Virus (SARS-CoV-2)	Class aera is 39-49 m^2, and 1.1-1.3 m^2 for each student	Two potential "index cases" caused 153 student and 25 staff members infected	No measurement results available.	9
Office and workplace	Speaking	Virus (SARS-CoV-2)	6-8 hours in a 19-story floor	94 cases (out of 216) reported in the call center	No measurement results available	10
	Ventilating air	Bacteria (L. pneumophila)	Not available	6 infection cases at a police headquarters building	No measurement results available	11
Choir	Singing and deeply breathing	Virus (SARS-CoV-2)	Rehearsal hall with 810 m^3 in volume, and the duration is 2.5 hours. Air exchange rate 0.5 h^{-1}.	One person with cold-like symptom caused a cluster outbreak, 53(out of 61) were confirmed to have contracted COVID-19	Emission rate of 970 ± 390 quanta/h	12

Table S3. Overview of sampling and biosensing techniques for on-site airborne pathogen detection.

Diseases	Pathogens	Types	Sampling techniques	Flowrate & Efficiency	Biosensing techniques	Limit of detection (LOD)	Highlights	Ref.
Novel coronavirus disease COVID-19	SARS-CoV-2	Virus	Integrated **condensational** particle growth and swirling **impingement** techniques	12.5 L/min, collection efficiency >90% for viral particles	Localized surface plasmon resonance (LSPR) biosensors with thermoplasmonic amplification	0.25 copies/µL in liquid and 6.7 copies/L in air	The airborne virus biosensing results were translated to probabilities of SARS-CoV-2 infection risk and to estimate maximum exposure durations to an acceptable risk threshold.	13
Novel coronavirus disease COVID-19	SARS-CoV-2	Virus	**Condensation** growth-based wet-wall **cyclone** sampler	1000 L/min	Nanobody-based ultrasensitive micro-immunoelectrode biosensor	7-35 viral RNA copies/m^3 in air	>95% collection efficiency for particles >1 µm; a cut-off diameter of 0.4 µm; 5 min detection time resolution.	14
Novel coronavirus disease COVID-19	SARS-CoV-2	Virus	**Filtration** based aerosol sampler	60 L/min	Continuous-Flow Electrochemical Bioassay	0.1 copies/µL in liquid	Quantitation of real-world HCoV-229E and SARS-CoV-2 in airborne particulate matters; Off-site biosensing.	15
Novel coronavirus disease COVID-19	SARS-CoV-2	Virus	PTF filter (**filtration**)	50–250 L/min	Microfluidic fluorescence system	10 copies/µL in liquid	Good precision (CV % ≤ 5.0%) in rapid detection of SARS-CoV-2.	16
Novel coronavirus disease COVID-19	SARS-CoV-2	Virus	Passive sampling setting	N.A.	Spark-induced plasma spectroscopy	1000 to 3000 PFU/mL	Real-time detection of virus propagation; instantaneous monitoring of high concentration coronavirus;	17
Novel coronavirus disease COVID-19	SARS-CoV-2	Virus	Impactor (**impaction**)	Breath flow (0.6L/s)	RT-qPCR on the sampling silicon chip	10-100 copies/ µL in liquid	Comparable clinical sensitivity and specificity to swab-based testing; potential for early diagnostics and transmission control.	18
Novel coronavirus disease COVID-19	SARS-CoV-2	Virus	**Filtration** collector integrated onto facemask	Human breath flow	qualitative colorimetric LAMP and antibody-based dot blot assays	Qualitative assay	Combining nucleic acid test and immunoassay test;	19
Respiratory disease	Human coronavirus-229E	Virus	**Electrostatic** air sampler	4–10 L/min; the ATH EC up to 67,000	qRT-PCR	0.4 PFU/mL in liquid	Enrich the aerosolized viral particles more than 10^6-fold; 10 min enrichment time.	20
Respiratory disease	Human coronavirus - 229E	Virus	**Electrostatic** air sampler	100 L/min (collection efficiency of 71 ± 7%)	qRT-PCR	10^3 copies/mL in liquid	Airborne viruses enriched by 3.5×10^5	21
Respiratory disease	Human coronaviruses-OC43	Virus	Condensation-based air sampler (condensation growth and **impaction**)	6 L/min	Electrochemical biosensor with carbon nanotube-coated porous paper	65.7 PFU/mL in liquid;	The TAT is 2 min for liquid-borne viruses; 10 min TAT for airborne viruses.	22

Diseases	Pathogens	Types	Sampling techniques	Flowrate & Efficiency	Biosensing techniques	Limit of detection (LOD)	Highlights	Ref.
Influenza	AIV/H1N1	Virus	**Impaction** on sampling pad	100 L/min (81.7% transfer efficiency to the detection zone)	Up-conversion nanoparticle labeling and NIR emission	$10^{4.294}$ EID50/m^3 in air	Integrated sampling/monitoring platform for on-site detection of airborne viruses; 20min TAT.	[23]
Influenza	AIV/H1N1	Virus	**Electrostatic** air sampler	4–10 L/min; ATH EC up to 70,400	qRT-PCR (offline analysis)	1.21 PFU/mL in liquid	Enrich the aerosolized viral particles more than 10^6-fold.	[20]
Influenza	AIV/H1N1	Virus	**Electrostatic** particle concentrator (EPC)	1.2 L/min	Electrochemical paper immunosensors	2.13 PFU/mL in liquid and 21.1 TCID50/m^3 in air	Comparable to qPCR but much quicker and more cost-effective; antigenicity loss upon exposure of electrostatic field.	[24]
Influenza	AIV/H1N1	Virus	Viable virus aerosol sampler (VIVAS) based on the **condensational** particle growth	6 L/min	Integrated RT-LAMP (integrated sample-preparation/amplification device (SPAD))	1 TCID50 H1N1 in 140 µL liquid; 1 TCID50/L in air	1 hour TAT; sensitivity can be improved by increasing the aerosol collection time.	[25]
Influenza	AIV/H1N1	Virus	Lab-made **electrostatic** air sampler	100 L/min, collection efficiency 0.7 ± 0.04;	Antibody functionalized electrochemical biosensor (with alkaline phosphatases)	0.01 PFU/mL in liquid	Sensitivity enhancement with electrochemical marker, alkaline phosphatases.	[26]
Influenza	H3N2	Virus	**Electrostatic** air sampler	4–10 L/min; ATH EC up to 69,000	qRT-PCR (offline analysis)	3.81 PFU/mL in liquid	Enrich the aerosolized viral particles more than 10^6-fold.	[20]
Influenza	H3N2	Virus	Disposable integrated **impaction** sampler	Collection efficiency >97% (300nm viral particles)	Fluorescent biosensors	8.3×10^3 viral particles/L	Labeling the virus with fluorescent dyes by the antigen–antibody reaction; TAT is 10 min.	[27]
Influenza	H3N2	Virus	**Electrostatic** air sampler	5 L/min	Electronical silicon nanowire (SiNW) sensor	10^4 viruses/L in air	Using target-specific antibodies for pathogens detection; fast quantification in a real-time manner.	[28]
Influenza	Influenza A virus	Virus	NIOSH 2-stage **cyclone** aerosol sampler	3.5 L/min	qRT-PCR (offline analysis)	10-100 copies/ µL in liquid	53% of detectable influenza virus particles were within the respirable aerosol fraction	[29]
Gastrointest-inal disease	Cryptospori-dium oocysts, and Giardia cysts	Bacteria	Microfilters (**Filtration**)	2 mL/min, with recovery rate of 90%	loop-mediated isothermal amplification (LAMP)	Not provided	Integration of the microfilter and a LAMP chip on a portable devic for airborne pathogens analysis.	[30]

Diseases	Pathogens	Types	Sampling techniques	Flowrate & Efficiency	Biosensing techniques	Limit of detection (LOD)	Highlights	Ref.
Tuberculosis	Mycobacterium tuberculosis	Bacteria	Microfluidic enrichment system	Serving for enrichment only, with 100% collection efficiency	Fluorescence imaging technique	10^3 cells/mL in liquid	Microfluidic system capable of airbornebacteria capture, enrichment, and rapid immunoassay; works in a sample-in-result-out manner.	31
Skin and soft tissue infections	Staphylococcus aureus	Bacteria	**Filtration** with copper mesh electrodes	1.5 L/min, 99.9% collection efficiency	EIS electrochemical sensors and SERS	1.26×10^3 CFU/m^3 in air	SERS for distinguish S. aureus ATCC 6538, E. coli JM109 and Candida albicans	32
Skin and soft tissue infections	Staphylococcus aureus	Bacteria	Electrostatic sampler	100 L/min with 71 ± 7% collection efficiency	Colorimetric analysis with CRISPR Cas12a	5 pM bacteria DNA in liquid	Multiplexing by changing the crRNA sequence; smartphone-based readout system for qualitative detection.	33
Respiratory tract infections	Bacillus atrophaeus	Bacteria	**Electrostatic** precipitator with electrowetting-on-dielectric concentrator	5 mL/min, 80% collection efficiency for 4 um particles and 50% for 1.5 um particles	HRP-immunoassay	4×10^4 cfu/ml	Assay times between six to ten minutes; detection of airborne pathogens for concerning chemical and biological warfare (CBW).	34
Plant diseases	Alternaria alternata	Fungi	BioSampler (**impingement**, BAMI)	12.5 L/min	Two-channel carbon nanotube field-effect transistors	10 pg/mL	BAMI enable the air-to-liquid sampling, solution transport, sensor regeneration, and detection of fungal particles.	35
Plant disease	Phakopsora pachyrhizi	Fungi	Microfiber for **filtration** sampling	30–100 L/min	Aptamer-based electrochemical sensor	100–200 spores per cm^2 of electrode area	Fiber electrode employed for both filtration sampling and electrochemical detection	36
Human asthma	Aspergillus niger	Fungi	Microfluidic enrichment system (inertial **impaction**)	Collection efficiency of 97.4% at 20 min and 82.5% at 60 min;	Immune-fluorescence imaging method	20 spores, equivalent to 300 spores/m in air	Whole analysis completed in 2-3 hours including 1 hour enrichment and 1 hour detection.	37
UTIs, diarrheal diseases, and pneumonia	Escherichia coli	Bacteria	Eight-stage Andersen sampler (inertial **impaction**)	28.3 L min/L	Surface enhanced Raman spectroscopy	N.A. utilized for estimating the viability of bacteria	living E. coli generate strong SERS signals; detection in a qualitative manner.	38
Emetic and diarrheal illnesses	Bacillus cereus; Micrococcus luteus	Bacteria	**Electrostatic** sampler	100 L/min with 71 ± 7% collection efficiency	Colorimetric analysis with CRISPR Cas12a	5 pM bacteria DNA in liquid	Multiplexing by changing the crRNA sequence; smartphone-based readout system for qualitative detection.	33

Abbreviation in the table: AIV, avian influenza virus; PCR, polymerase chain reaction; LAMP, loop-mediated isothermal amplification; PTFE, polytetrafluoroethylene; TAT, turnaround time, EID50, 50% egg infectious dose; TCID50, the 50% tissue culture infectious dose; CV, coefficient of variation, ATH, aerosol-to-hydrosol; EC, enrichment capacity; NIOSH, National Institute for Occupational Safety and Health; UTIs, urinary tract infections.

Reference:

1. L. Goldberg, Y. Levinsky, N. Marcus, V. Hoffer, M. Gafner, S. Hadas, S. Kraus, M. Mor and O. Scheuerman, *Open Forum Infectious Diseases*, 2021, **8**, ofab036.
2. M. Klompas, M. A. Baker, C. Rhee, R. Tucker, K. Fiumara, D. Griesbach, C. Bennett-Rizzo, H. Salmasian, R. Wang and N. Wheeler, *Ann. Intern. Med.*, 2021, **174**, 794-802.
3. T. Demuyser, E. De Cock and E. Sermijn, *Journal of Infection and Public Health*, 2019, **12**, 904-906.
4. R. Alon, M. Sportiello, S. Kozlovski, A. Kumar, E. C. Reilly, A. Zarbock, N. Garbi and D. J. Topham, *Nature Reviews Immunology*, 2021, **21**, 49-64.
5. H. Gu, P. Krishnan, D. Y. Ng, L. D. Chang, G. Y. Liu, S. S. Cheng, M. M. Hui, M. C. Fan, J. H. Wan and L. H. Lau, *Emerging infectious diseases*, 2022, **28**, 460.
6. Y. G. Li, H. Qian, J. Hang, X. G. Chen, P. Cheng, H. Ling, S. Q. Wang, P. Liang, J. S. Li, S. L. Xiao, J. J. Wei, L. Liu, B. J. Cowling and M. Kang, *Building and Environment*, 2021, **196**.
7. C. Ou, S. Hu, K. Luo, H. Yang, J. Hang, P. Cheng, Z. Hai, S. Xiao, H. Qian and S. Xiao, *Building and environment*, 2022, **207**, 108414.
8. N. C. Khanh, P. Q. Thai, H. L. Quach, N. A. H. Thi, P. C. Dinh, T. N. Duong, L. Q. T. Mai, N. D. Nghia, T. A. Tu, L. N. Quang, T. D. Quang, T. T. Nguyen, F. Vogt and D. D. Anh, *Emerging Infectious Diseases*, 2020, **26**, 2617-2624.
9. C. Stein-Zamir, N. Abramson, H. Shoob, E. Libal, M. Bitan, T. Cardash, R. Cayam and I. Miskin, *Eurosurveillance*, 2020, **25**, 2001352.
10. S. Y. Park, Y. M. Kim, S. Yi, S. Lee, B. J. Na, C. B. Kim, J. I. Kim, H. S. Kim, Y. B. Kim, Y. Park, I. S. Huh, H. K. Kim, H. J. Yoon, H. Jang, K. Kim, Y. Chang, I. Kim, H. Lee, J. Gwack, S. S. Kim, M. Kim, S. Kweon, Y. J. Choe, O. Park, Y. J. Park and E. K. Jeong, *Emerging Infectious Diseases*, 2020, **26**, 1666-1670.
11. M. Omahony, A. Lakhani, A. Stephens, J. G. Wallace, E. R. Youngs and D. Harper, *Epidemiology and Infection*, 1989, **103**, 285-292.
12. S. L. Miller, W. W. Nazaroff, J. L. Jimenez, A. Boerstra, G. Buonanno, S. J. Dancer, J. Kurnitski, L. C. Marr, L. Morawska and C. Noakes, *Indoor Air*, 2021, **31**, 314-323.
13. G. Qiu, M. Spillmann, J. Tang, Y.-B. Zhao, Y. Tao, X. Zhang, H. Geschwindner, L. Saleh, W. Zingg and J. Wang, *Advanced Science*, 2022, **9**.
14. J. V. V. Puthussery, D. P. P. Ghumra, K. R. R. McBrearty, B. M. M. Doherty, B. J. J. Sumlin, A. Sarabandi, A. G. Mandal, N. J. J. Shetty, W. D. D. Gardiner, J. P. P. Magrecki, D. L. L. Brody, T. J. J. Esparza, T. L. L. Bricker, A. C. M. Boon, C. M. M. Yuede, J. R. R. Cirrito and R. K. K. Chakrabarty, *Nature Communications*, 2023, **14**.
15. F. Jiang, B. Liu, Y. Yue, Y. Tao, Z. Xiao, M. Li, Z. Ji, J. Tang, G. Qiu, M. Spillmann, J. Cao, L. Zhang and J. Wang, *Advanced Science*, 2023, **10**.
16. H. Xiong, X. Ye, Y. Li, J. Qi, X. Fang and J. Kong, *Anal. Chem.*, 2021, **93**, 4270-4276.
17. J. H. Yang, J. Jung, S. Kim, Y. Cho and J. J. Yoh, *Sci. Total Environ.*, 2021, **770**.
18. T. Stakenborg, J. Raymenants, A. Taher, E. Marchal, B. Verbruggen, S. Roth, B. Jones, A. Yurt, W. Duthoo, K. Bombeke, M. Fauvart, J. Verplanken, R. S. Wiederkehr, A. Humbert, C. Dang, E. Vlassaks, A. L. J. Uribe, Z. Luo, C. Liu, K. Zinoviev, R. Labie, A. D. Frederiks, J. Saldien, K. Covens, P. Berden, B. Schreurs, J. Van Duppen, R. Hanifa, M. Beuscart, V. Pham, E. Emmen, A. Dewagtere, Z. Lin, M. Peca, Y. El Jerrari, C. Nawghane, C. Arnett, A. Lambrechts, P. Deshpande, K. Lagrou, P. De Munter, E. Andre, N. Van den Wijngaert and P. Peumans, *Biosens. Bioelectron.*, 2022, **217**.

19. F. Soto, M. O. Ozen, C. F. Guimaraes, J. Wang, K. Hokanson, R. Ahmed, R. L. Reis, R. Paulmurugan and U. Demirci, *Acs Applied Materials & Interfaces*, 2021, **13**, 41445-41453.
20. H. R. Kim, S. An and J. Hwang, *Biosens. Bioelectron.*, 2020, **170**.
21. H. R. Kim, S. An and J. Hwang, *J. Hazard. Mater.*, 2021, **412**.
22. D. Lee, J. Jang and J. Jang, *J. Hazard. Mater.*, 2023, **458**.
23. I. Lee, Y. Seok, H. J. Jung, B. Yang, J. Lee, J. Kim, H. Pyo, C. S. Song, W. Choi, M. G. Kim and J. Lee, *Acs Sensors*, 2020, **5**, 3915-3922.
24. J. Bhardwaj, M. W. Kim and J. Jang, *Environ. Sci. Technol.*, 2020, **54**, 10690-10702.
25. X. Jiang, J. C. Loeb, M. H. Pan, T. B. Tilly, A. Eiguren-Fernandez, J. A. Lednicky, C. Y. Wu and Z. H. Fan, *Anal. Chim. Acta*, 2021, **1165**.
26. J. Shin, H. R. Kim, P. K. Bae, H. Yoo, J. Kim, Y. Choi, Y. B. Shin, J. Hwang and S. Hong, *Sci. Rep.*, 2021, **11**.
27. K. Takenaka, S. Togashi, R. Miyake, T. Sakaguchi and M. Hide, *Journal of Breath Research*, 2016, **10**.
28. F. Shen, M. Tan, Z. Wang, M. Yao, Z. Xu, Y. Wu, J. Wang, X. Guo and T. Zhu, *Environ. Sci. Technol.*, 2011, **45**, 7473-7480.
29. F. M. Blachere, W. G. Lindsley, T. A. Pearce, S. E. Anderson, M. Fisher, R. Khakoo, B. J. Meade, O. Lander, S. Davis, R. E. Thewlis, I. Celik, B. T. Chen and D. H. Beezhold, *Clin. Infect. Dis.*, 2009, **48**, 438-440.
30. Q. Liu, X. Zhang, Y. Yao, W. Jing, S. Liu and G. Sui, *Sensors and Actuators B-Chemical*, 2018, **258**, 1138-1145.
31. W. Jing, X. Jiang, W. Zhao, S. Liu, X. Cheng and G. Sui, *Anal. Chem.*, 2014, **86**, 5815-5821.
32. X. Su, R. Ren, Y. Wu, S. F. Li, C. Ge, L. L. Liu and Y. Xu, *Biosens. Bioelectron.*, 2021, **176**.
33. J. Ki, I. H. Kwon, J. Lee, J. Lim, S. Jang, S. U. Son, S. B. Seo, S. Y. Oh, T. Kang, J. Jung, K. G. Lee, J. Hwang and E.-K. Lim, *J. Hazard. Mater.*, 2023, **460**, 132398.
34. L. Coudron, M. B. McDonnell, I. Munro, D. K. McCluskey, I. D. Johnston, C. K. L. Tan and M. C. Tracey, *Biosens. Bioelectron.*, 2019, **128**, 52-60.
35. J. Kim, J.-H. Jin, H. S. Kim, W. Song, S.-K. Shin, H. Yi, D.-H. Jang, S. Shin and B. Y. Lee, *Environ. Sci. Technol.*, 2016, **50**, 5163-5171.
36. V. Krivitsky, E. Granot, Y. Avidor, E. Borberg, R. T. Voegele and F. Patolsky, *Acs Sensors*, 2021, **6**, 1187-1198.
37. X. Li, X. Zhang, Q. Liu, W. Zhao, S. Liu and G. Sui, *Acs Sensors*, 2018, **3**, 2095-2103.
38. M. A. Tahir, X. Zhang, H. Cheng, D. Xu, Y. Feng, G. Sui, H. Fu, V. K. Valev, L. Zhang and J. Chen, *Analyst*, 2020, **145**, 277-285.
